# Taxonomic Synopsis of the Ponto-Mediterranean Ants of *Temnothorax nylanderi* Species-Group

**DOI:** 10.1371/journal.pone.0140000

**Published:** 2015-11-04

**Authors:** Sándor Csősz, Jürgen Heinze, István Mikó

**Affiliations:** 1 Entomology, California Academy of Sciences, San Francisco, California, United States of America; 2 Zoology / Evolutionary Biology, University of Regensburg, Regensburg, Germany; 3 Frost Entomological Museum, Pennsylvania State University, University Park, Pennsylvania, United States of America; Arizona State University, UNITED STATES

## Abstract

In the current revisionary work, the *Temnothorax nylanderi* species-group of myrmicine ants is characterized. Eighteen species belonging to this group in the Ponto-Mediterranean region are described or redefined based on an integrative approach that combines exploratory analyses of morphometric data and of a 658bp fragment of the mitochondrial gene for the cytochrome c oxidase subunit I (CO I). The species group is subdivided into five species complexes: *T*. *angustifrons* complex, *T*. *lichtensteini* complex, *T*. *nylanderi* complex, *T*. *parvulus* complex, *T*. *sordidulus* complex, and two species, *T*. *angulinodis* **sp. n.** and *T*. *flavicornis* (Emery, 1870) form their own lineages. We describe seven new species (*T*. *angulinodis* **sp. n.**, *T*. *angustifrons* **sp. n.**, *T*. *ariadnae* **sp. n.**, *T*. *helenae* **sp. n.**, *T*. *lucidus* **sp. n.**, *T*. *similis* **sp. n.**, *T*. *subtilis* **sp. n.)**, raise *T*. *tergestinus* (FINZI, 1928) **stat.n.** to species level, and propose a new junior synonymy for *T*. *saxonicus* (SEIFERT, 1995) **syn.n.** (junior synonym of *T*. *tergestinus*). We describe the worker caste and provide high quality images and distributional maps for all eighteen species. Furthermore, we provide a decision tree as an alternative identification key that visually gives an overview of this species-group. We make the first application to Formicidae of the Semantic Phenotype approach that has been used in previous taxonomic revisions.

## Introduction

The myrmicine genus *Temnothorax* (formerly synonymized with *Leptothorax*) is one of the most speciose ant genera on earth, with 380 valid species and 47 subspecies worldwide (http://www.antcat.org). It is especially well represented in the European fauna. Members of the genus occur in a great variety of habitats and display a considerable range of social behavior [1] and associations with other organisms [2]. Because of their small colony size and easy maintenance in the lab *Temnothorax* species establish model systems in insect behavioral ecology and sociobiology, including studies on self-organized decision making [3], [4], [5], [6], nest construction [7], [8], nest moving [9], [10], division of labor [11], the regulation of reproduction [12], [13], [14], [15], [16] [17], [18], and host-parasite coevolution [19], [20], [21].

Thanks to intensive studies during recent years [22], [23], [24], [25] the European ant fauna is relatively well explored. *Temnothorax* has long been the focus of ant taxonomy, and earlier taxonomic studies are available for particular geographic areas [26], [27], [28], [29]. The *Temnothorax nylanderi* species-group is particularly diverse in the Ponto-Mediterranean region (i.e., the basin of the Mediterranean Sea and Black Sea) and its systematics is still very challenging. Those few species that range as far northwest as England and Central Europe are well understood and adequately described by modern taxonomic methodology [30], [31], [32], [33]. In North Africa and in part the Iberian Peninsula, the *T*. *nylanderi* species-group is replaced by the *Temnothorax laurae* species-group, its vicariant sister group [34], [35].

The high diversity of the *T*. *nylanderi* species-group in the Ponto-Mediterranean region is coupled with a large number of cryptic species [36], [33], whose broadly overlapping morphological characters hamper the definition of species boundaries based on conventional techniques. Therefore, in the present study we delineate species boundaries in an integrative fashion [37], using multiple lines of evidence, including Numeric Morphology Based Alpha Taxonomy (NUMOBAT) and molecular analyses.

In our study we initially recognized morphological patterns using the exploratory data analysis tool NC-clustering [38], which allows inference of the boundaries of morphological entities without prior hypotheses about the number of clusters or the classification of a particular sample. The performance of such exploratory data analysis tools allowed formulation of sound hypotheses about formerly unrecognized morphologically cryptic taxa in ants [39], [33]. The initially inferred patterns are subsequently tested by confirmatory data analyses. To strengthen the consistency of conclusions by the complex morphometric analyses, we in parallel analyzed a fragment of the mitochondrial gene for the cytochrome c oxidase subunit I (CO I) in specimens from all identified morphological clusters.

Our data reveal cryptic diversity in this species-group of the genus *Temnothorax* and help to better understand the biogeographic patterns of this genus in the Ponto-Mediterranean region.

## Materials and Methods

In the present study, we recorded 22 continuous morphometric traits in 1693 worker individuals belonging to 526 nest samples. Specimens for the present study were borrowed from public and private collections, see the list of institutions below. Our study did not involve endangered or protected species. Label information of type material is given for each taxon in the section *type material investigated*. Non-type material that was morphometrically examined in this revision is listed in S1 Table. Samples displayed in the dendrogram are characterized by sample-specific abbreviations (e.g., **GRE:Levidi-10S-20000427-123**) generated from the original label information as follows:

A three-letter country code (in capital letters separated from the following part by a colon), the nearest settlement given in the label (separated from the following parts by hyphens), the distance and direction from it, the sampling date in alpha-numeric format and a final unique field sample identifier.

All images presented are available online on AntWeb (http://www.antweb.org) and can be uniquely identified by their specimen-level codes affixed to each pin. Information about the taxonomic history of the taxa redefined below is based on B. Bolton in AntCat (http://www.antcat.org). Distribution maps were generated by QGIS 2.4.0 software [40]. For statistical comparisons of morphological data we used R [41].

Due to the limited number of queens and males available in the present study, our revision is based on the worker caste. A worker-based revision is further facilitated by the fact that the type specimens of most of the existing taxa are workers.

### Nomenclature Acts

The electronic edition of this article conforms to the requirements of the amended International Code of Zoological Nomenclature, and hence the new names contained herein are available under that Code from the electronic edition of this article. This published work and the nomenclatural acts it contains have been registered in ZooBank, the online registration system for the ICZN. The ZooBank LSIDs (Life Science Identifiers) can be resolved and the associated information viewed through any standard web browser by appending the LSID to the prefix “http://zoobank.org/”. The LSID for this publication is: urn:lsid:zoobank.org:pub:06017DCE-7B39-4F89-9EB5-742609AEDCBA. The electronic edition of this work was published in a journal with an ISSN, and has been archived and is available from the following digital repositories: PubMed Central, LOCKSS.

### Abbreviations of depositories

We investigated specimens from the following institutions (abbreviations after [42]; curators in parentheses after institutions):

ASPC Private collection of Andreas Schulz, Leverkusen, Germany;

CASC California Academy of Sciences, San Francisco, California, U.S.A. (B. L. Fisher);

HNHM Hungarian Natural History Museum, Budapest, Hungary (Z. Vas);

IRSNB Institut Royal des Sciences Naturelles de Belgique, Brussels, Belgium (W. Deckonick);

MCZ Museum of Comparative Zoology, Cambridge, Massachusetts, U.S.A. (S. Cover);

MSNG Museo Civico di Storia Naturale “Giacomo Doria”, Genova, Italy (M. Tavano);

NHMB Naturhistorisches Museum, Basel, Switzerland (D. Burckhard);

NHMK Landesmuseum für Kärnten, Klagenfurt, Austria (C. Wieser);

SIZK Schmalhausen Institute of Zoology, Kiev, Ukraine (A. G. Radchenko);

SMF Forschungsinstitut und Naturmuseum Senckenberg, Frankfurt am Main, Germany (J. P. KOPELKE);

SMNG Staatliches Museum für Naturkunde, Görlitz, Germany (B. Seifert);

SMNK Staatliches Museum für Naturkunde Karlsruhe, Germany (M. Verhaagh);

### Molecular analysis

Partial sequences of the mitochondrial gene CO I were used to corroborate the morphology-based definition of new taxa. We managed to amplify DNA of most of the taxa inspected by morphometry, but amplification failed for *T*. *sordidulus* and *T*. *saxonicus* and no material was available of *T*. *carinthiacus*, *T*. *angulinodis* sp.n. and *T*. *angustifrons* sp. n. DNA was isolated using the CTAB method including proteinase K digestion [43]. We amplified a 658 bp fragment of the CO I gene using the primers LCO1490 and HCO2198 [44]. For PCR we used the GO-Taq Hot Start Master Mix from Promega (Madison, WI) with a final primer concentration of 0.7 μM and 1 μl DNA. PCR conditions were as described previously [33], i.e., an initial denaturation step at 94°C for 240 s, 38 cycles of denaturation at 94°C, 45 s; annealing at 50°C, 45 s; elongation at 72°C, 1 min; and a final step of 72°C, 300 s. DNA was sequenced in an ABI 3100 capillary sequencer (Applied Biosystems, Foster City, CA) and sequences were aligned using Bioedit [45]. Ambiguous parts at the fragment ends were removed, resulting in a fragment length of 652 bp for further analysis.

MrModeltest GUI (available under http://genedrift.org) in conjuncture with PAUP 4.0b10 [46] estimated HKY + I + G as best model for the Bayesian analysis, which was performed with MrBayes 3.2.1 [47] with three heated and one cold Markov chains. Default heating parameters were set at 0.2 with an MCMC length of 3,000,000 generations. The first 500 generations (burn-in) were discarded and every 100th generation was sampled. The phylogenetic tree was drawn using FigTree version 1.2.2 (available at http://tree.bio.ed.ac.uk/software). GenBank accession numbers are given in S2 Table.

### Protocol of morphometric character recording

**Fig 1:**
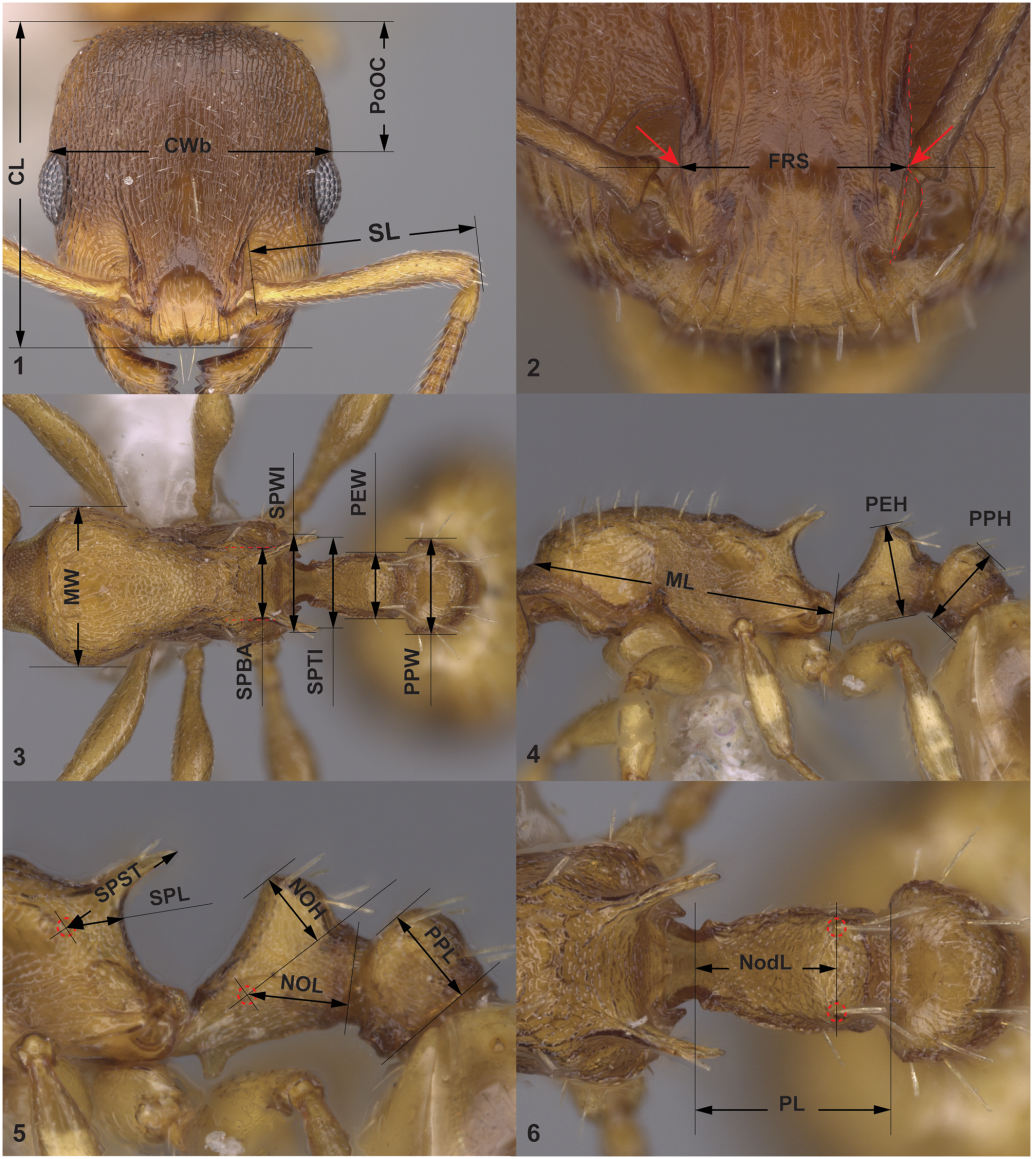
Definition of morphological characters of workers of *Temnothorax nylanderi species*-complex. Head in dorsal view (A) with measurement lines for CL, CWb, PoOC and SL; frontal region of the head dorsum (B) with measurement lines for FRS; dorsal view of mesosoma (C) with measurement lines for MW, PEW, PPW, SPBA, SPTI and SPWI; lateral view of mesosoma (D) with measurement lines for ML, PEH and PPH; lateral view of propodeum, petiole and postpetiole (E) with measurement lines for HOH, NOL, PPL, SPL and SPST; lateral view of propodeum, petiole and postpetiole (F) with measurement lines for NoDL and PPL.

Morphometric characters are defined as in [48]. All measurements were made in μm using a pin-holding stage, permitting rotations around X, Y, and Z axes. An Olympus SZX9 stereomicroscope was used at a magnification of x150 for each character, allowing an estimated precision of ± 2 μm. Morphometric data (in μm) are provided in S3 Table. All measurements were made by the first author. Measured characters are defined and abbreviated as follows:

**CL**: maximum cephalic length in median line; the head must be carefully tilted to the position with the true maximum. Excavations of hind vertex and/or clypeus reduce CL (Fig 1A).

**CS**: cephalic size; the arithmetic mean of CL and CWb.

**CWb**: maximum width of head capsule, measured just posterior to the eyes (Fig 1A).

**EL**: maximum diameter of the eye.

**FRS**: distance of the frontal carinae immediately caudal of the posterior intersection points between frontal carinae and the lamellae dorsal to the torulus. If these dorsal lamellae do not laterally surpass the frontal carinae, the deepest point of scape corner pits may be taken as reference line. These pits take up the inner corner of scape base when the scape is fully switched caudad and produce a dark triangular shadow in the lateral frontal lobes immediately posterior to the dorsal lamellae of scape joint capsule (Fig 1B).

**ML**: mesosoma length from caudalmost point of propodeal lobe to transition point between anterior pronotal slope and anterior propodeal shield (preferentially measured in lateral view; if the transition point is not well defined, use dorsal view and take the center of the dark-shaded borderline between pronotal slope and pronotal shield as anterior reference point) (Fig 1D).

**MW**: maximum mesosoma width; this is in workers pronotal width (Fig 1C).

**NOdL**: anterior length of petiole measured in dorsal view. Distance from the centre of anteriormost seta pit on the petiolar node to the level of the constriction of articulation condyle with propodeum. Measuring requires a change of focus from above (seta pit) to below (constriction). Dorsal view is achieved when the dorsalmost point of anterior petiolar peduncle at the level of its strongest constriction and the dorsalmost point of caudal petiolar margin are in the same focal level (Fig 1F).

**NOH**: maximum height of the petiolar node, measured in lateral view from the uppermost point of the petiolar node perpendicular to a reference line set from the petiolar spiracle to the imaginary midpoint of the transition between dorso-caudal slope and dorsal profile of caudal cylinder of the petiole (Fig 1E).

**NOL**: length of the petiolar node, measured in lateral view from petiolar spiracle to dorso-caudal corner of caudal cylinder. Do not erroneously take as reference point the dorso-caudal corner of the helcium, which is sometimes visible (Fig 1E).

**PEH**: maximum petiole height. The chord of ventral petiolar profile at node level is the reference line perpendicular to which the maximum height of petiole is measured (Fig 1D).

**PEW**: maximum width of petiole (Fig 1C).

**PL**: total petiole length measured in dorsal view; distance between the dorsalmost point of caudal petiolar margin and the dorsalmost point of anterior petiolar peduncle at the transversal level of its strongest constriction. Positioning of petiole as in NOdL (Fig 1F).

**PoOC**: postocular distance. Use a cross-scaled ocular micrometer and adjust the head to the measuring position of CL. Caudal measuring point: median occipital margin; frontal measuring point: median head at the level of the posterior eye margin (Fig 1A).

**PPH**: maximum height of the postpetiole in lateral view measured perpendicularly to a line defined by the linear section of the segment border between dorsal and ventral petiolar sclerite (Fig 1D).

**PPL**: maximum length of the postpetiole measured in lateral view perpendicular to the straight section of lateral postpetiolar margin (Fig 1E).

**PPW**: maximum width of postpetiole (Fig 1C).

**SL**: maximum straight line scape length excluding the articular condyle (Fig 1A).

**SPL**: minimum distance between the center of propodeal spiracle and the subspinal excavation measured in lateral view (i.e. the same view that is applied to measure ML). Note: in lateral view propodeal spiracle and the caudal margin of propodeal declivity might not be in the same focal level, hence slight adjust might be necessary while measuring SPL between the two endpoints (Fig 1E).

**SPBA**: the smallest distance of the lateral margins of the spines at their base. This should be measured in dorsofrontal view, since the wider parts of the ventral propodeum do not interfere with the measurement in this position. If the lateral margins of spines diverge continuously from the tip to the base, a smallest distance at base is not defined. In this case, SPBA is measured at the level of the bottom of the interspinal meniscus (Fig 1C).

**SPST**: distance between the center of propodeal stigma and spine tip. The stigma centre refers to the midpoint defined by the outer cuticular ring but not to the centre of real stigma opening that may be positioned eccentrically (Fig 1E).

**SPTI**: the distance of spine tips in dorsal view; if spine tips are rounded or truncated, the centers of spine tips are taken as reference points (Fig 1C).

**SPWI**: maximum distance between outer margins of spines; measured in same position as SPBA (Fig 1C).

The **deviation of propodeal spines from longitudinal mesosomal axis** (i.e. angle between the spines and the mesosomal axis) is often of high diagnostic value within the *Temnothorax nylanderi* species-group. Longitudinal mesosomal axis is defined by Seifert et al. [39] as follows: *in lateral view as straight line from the center of propodeal lobe to the border point between anterior pronotal shield and propleuron*.

### Semantic phenotype approach

We represent natural language phenotypes in an EQ format: Entity attribute: value (S6 Table). Semantic statements of natural language phenotypes (S6 Table) were composed in Protégé 5.0 (http://protege.stanford.edu/) using the OWL Manchester syntax (http://www.w3.org/TR/owl2-manchester-syntax/) following [49] and [50]. The full data set, represented in OWL (Web Ontology Language; http://www.w3.org/TR/owl2-overview/ last accessed February 4, 2014), was deposited as a Resource Description Framework (RDF)-XML file (http://www.w3.org/TR/REC-rdf-syntax/ in the Dryad repository (http://dx.doi.org/10.5061/dryad.xxxx).

Hereby we propose two new template EQ expression as an expansion of the semantic statement types described by Balhoff et al. [49] and Mikó et al. [50]:

Angle between anatomical lines

Angles between anatomical lines are often used in taxonomic treatments of Formicidae:

NL: Dorsal profile of petiolar node contour line angle value to frontal profile of petiole contour line in lateral view: 72–82°”

We expressed angle value between anatomical lines using PATO’s angle and UO’s degree with the combination of our previously proposed relative and absolute measurement phenotypes:

SP: ‘has part' some ('dorsal petiolar profile contour line' and (bearer_of some (angle and ('is quality measured as' some (('has measurement unit label' value degree) and ('has measurement value' some (float[> = 72.0f] and float[< = 82.0f])))) and (towards some 'anterior profile of petiolar node contour line'))))

Coloration

We present color hue and intensity as separated entity attributes:

NL: Body color: brown

SP: 'has part' some (body and ('bearer of' some brown))

and

NL: Body color pattern: mesosoma, antenna and legs excluding femora, waist and anterior region of 1st gastral tergite lighter than head, femora and posterior region of gaster.

SP: ('has part' some (cranium and ('bearer of' some ('color brightness' and (similar_in_magnitude_relative_to some ('color brightness' and ('inheres in' some femur))))))) and ('has part' some (cranium and ('bearer of' some ('color brightness' and (similar_in_magnitude_relative_to some ('color brightness' and ('inheres in' some ('posterior region' and ('part of' some gaster))))))))) and ('has part' some (mesosoma and ('bearer of' some ('color brightness' and (increased_in_magnitude_relative_to some ('color brightness' and ('inheres in' some cranium))))))) and ('has part' some (mesosoma and ('bearer of' some ('color brightness' and (similar_in_magnitude_relative_to some ('color brightness' and ('inheres in' some 'abdominal segment 1'))))))) and ('has part' some (mesosoma and ('bearer of' some ('color brightness' and (similar_in_magnitude_relative_to some('color brightness' and ('inheres in' some antenna))))))) and ('has part' some (mesosoma and ('bearer of' some ('color brightness' and (similar_in_magnitude_relative_to some ('color brightness' and ('inheres in' some ('anterior region' and ('part of' some gaster))))))))) and ('has part' some ((not (femur)) and ('part of' some (leg and ('bearer of' some ('color brightness' and (similar_in_magnitude_relative_to some ('color brightness' and ('inheres in' some mesosoma)))))))))

### Morphometric hypothesis formation and testing

The complexity of the *Temnothorax nylanderi* species-group required a complex work flow for morphometric pattern recognition. Combined stepwise analyses were done to achieve the best results. Iterated confirmatory analyses (Steps 2 and 3) helped to support the position of various samples predicted by prior steps (Step 1).

#### Step 1: Exploratory analyses of morphometric data by NC clustering algorithms

We first used an exploratory data analysis of continuous morphometric traits to assess the occurrence of cryptic diversity within the East-Mediterranean *Temnothorax nylanderi* species-group. A hierarchical, agglomerative nesting method, Nest Centroid Clustering (NC clustering) was employed to reveal complex patterns in the complete dataset [38]. NC clustering allows assessing the number of morphologically meaningful clusters (i.e., taxa) and disclosing the structure of the whole species-group without prior hypotheses. Initial conclusions about morphological patterns were drawn from the output of NC-UPGMA clustering. The script for this analysis, written in R, is freely available at: http://sourceforge.net/projects/agnesclustering/.

#### Step 2: Deriving hypotheses from NC-clustering and checking these by a confirmatory Linear Discriminant Analysis (LDA)

To increase the reliability of the recognized patterns we applied parallel runs of NC-UPGMA and NC-Ward clustering. In a confirmative LDA, run on the level of individual workers, classification hypotheses were imposed for all samples congruently classified by both methods. Wild-card settings (i.e., no hypothesis imposed) were given to those samples whose classifications differed between the methods. The confirmative LDA was run as an iterative process and sample means of posterior probabilities formed the basis for the finally accepted classification. No samples with ambiguous position remained (for details see [38], and all taxa in this revision meet the criteria of the *pragmatic species hypothesis* [51]. The model is validated by Leave One Out Cross Validation.

#### Step 3: Application of confirmatory Linear Discriminant Analyses (LDA) on separate species complexes

After prior species hypotheses formulated by NC-clustering (Step 1) had been confirmed by cumulative LDA (Step 2), we applied an a posteriori step of Linear Discriminant Analysis on every species-complex with more than one species. Posterior analysis allows confirmation of a priori groups suggested by NC-clustering and replacing erroneously allocated samples. Taxa within species-complexes can thus be visualized in two dimensional scatterplots. The second purpose of this ultimate statistical step is to provide readers with the simplest numeric function for species determination with an acceptably low (< 5%) error rate. Researchers who determine ants on a daily basis particularly benefit from the reduction of the number of characters in numeric determination tools.

#### Cross Validation Decision Tree

The key to worker caste was generated by a Cross Validation Decision Tree algorithm through the package „rpart” [52] in R [41] based on nest sample means of body size ratios. Decision trees yield a dichotomous structure in which each node represents a test of continuous morphometric traits (decision taken after computing all attributes). The applicability of attributes for each node generated by the model was validated by Leave One Out Cross Validation. The reliability of characters for each node was also tested manually by t-tests.

## Results and Discussion

### Multivariate Analyses of Numeric Morphology—Hypothesis formation and Confirmatory analyses

**Fig 2:**
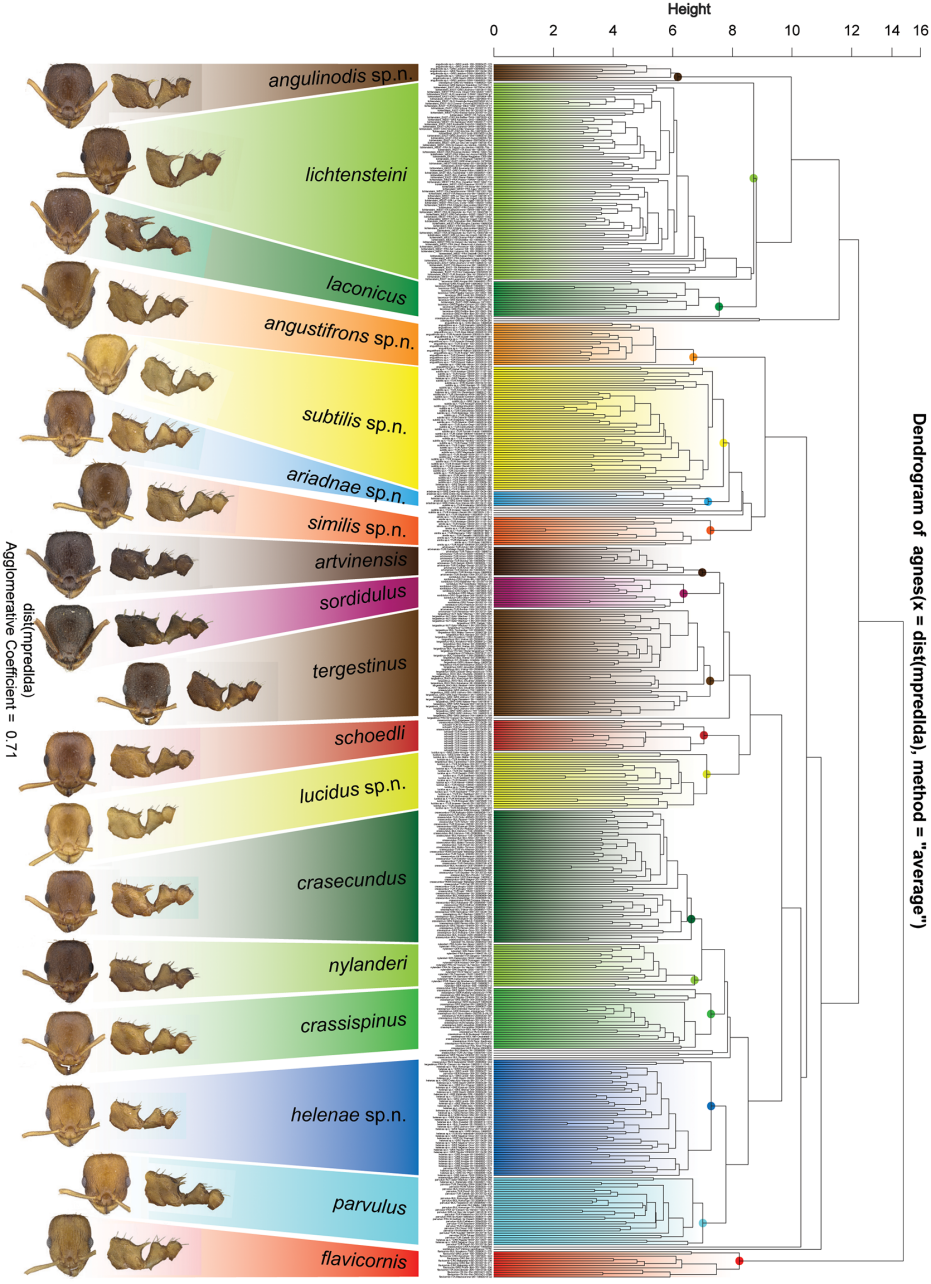
Dendrogram of NC-UPGMA clustering of all lineages of *Temnothorax nylanderi* species-group. Separate colors represent different species. Sequence of information in the string designating the samples: final species hypothesis confirmed by confirmatory LDA _ country—locality—date—sample number.

An exploratory NC-Clustering revealed the existence of eighteen clearly separated clusters within the available specimens of Ponto-Caspian populations of the *Temnothorax nylanderi* species-group (Fig 2). This pattern was confirmed by a cumulative Linear Discriminant Analysis. The confirmatory analyses correctly classified 94% of the 1693 individuals. Validation by Leave One Out Cross Validation yielded a 93% classification success rate, which can be considered acceptable in such a complex, multidimensional discriminant space. The vast majority of species showed a perfect or nearly perfect separation from other morphospecies treated in this revision: *T*. *angulinodis* sp.n. (100%), *T*. *angustifrons* sp.n. (98.5%), *T*. *ariadnae* sp.n. (100%), *T*. *artvinensis* (95.8%), *T*. *flavicornis* (100%), *T*. *laconicus* (94.9%), *T*. *lichtensteini* (97.3%), *T*. *lucidus* sp.n. (94.3%), *T*. *parvulus* (97.1%), *T*. *schoedli* (93.8%), *T*. *similis* sp.n. (94.7%), *T*. *sordidulus* (95.6%) and *T*. *subtilis* sp.n. (95.5%).

A few others yielded a lower classification success: *T*. *crasecundus* (89.6%), *T*. *crassispinus* (90.4%), *T*. *helenae* sp.n. (91.3%), *T*. *nylanderi* (90%), and *T*. *tergestinus* (89.9%). This is probably not surprising, given the the high morphological similarity among some of the closely related species. Some taxa could be separated convincingly only by confirmatory LDA of nest sample means: 98.5% of the 528 nest samples were correctly classified by this analysis. Again a few species, *T*. *crasecundus* (98.2%), *T*. *crassispinus* (97.47), *T*. *laconicus* (93.8%), *T*. *lichtensteini* (98.8%), *T*. *subtilis* sp.n. (98.2%) and *T*. *tergestinus* (94.0%) attained somewhat lower rate, but nest samples of all other species treated in this revision were correctly classified (100%).

The species hypothesis generated by the NC-clustering, with 18 existing morphospecies, was thereby confirmed by LDA and can be considered as the final species hypothesis in this revision.

### Molecular data

**Fig 3:**
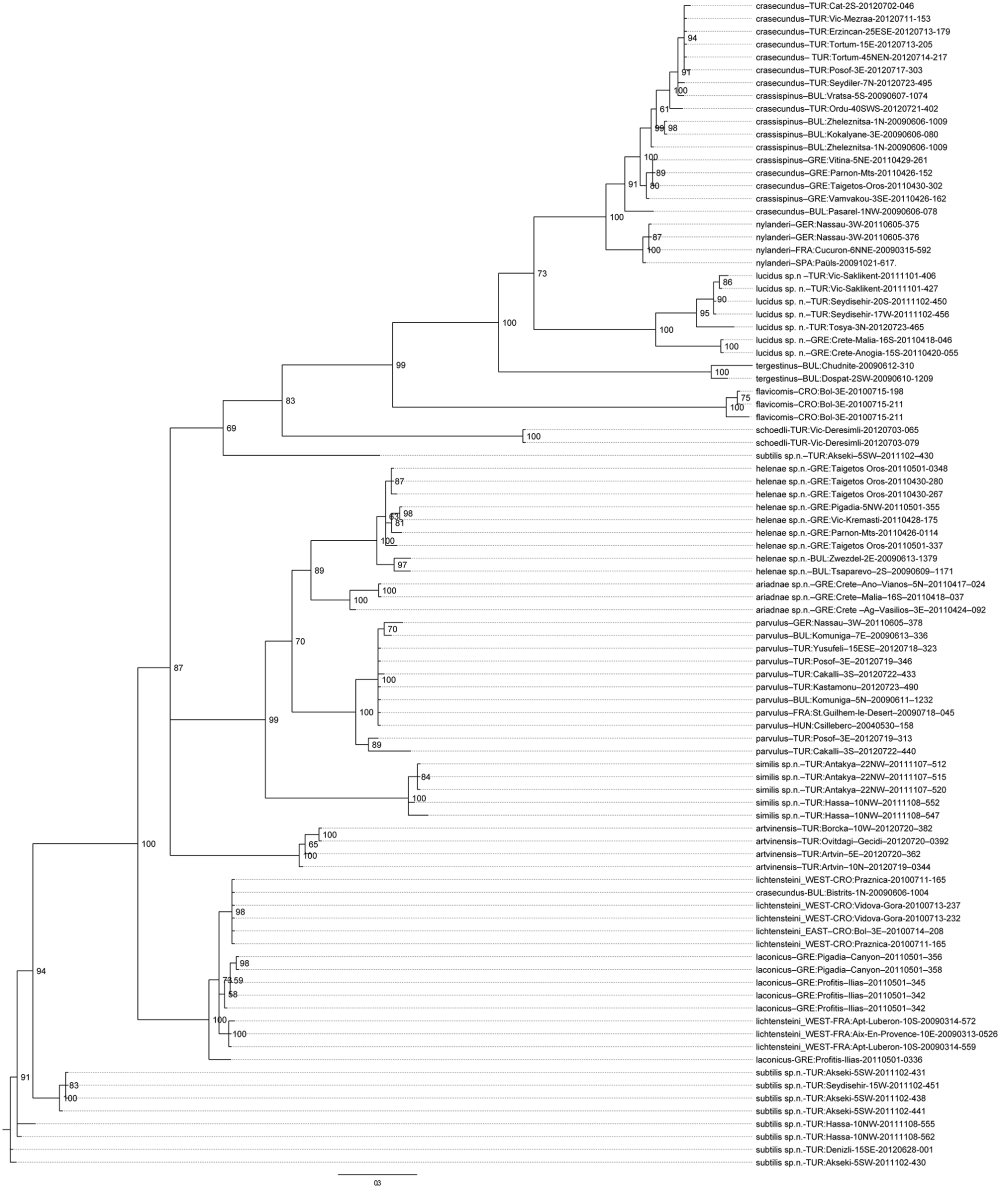
Bayesian consensus tree. Bayesian consensus tree from two independent analyses of a 658 bp fragment of the mitochondrial CO I gene of taxa in *Temnothorax nylanderi* species-group. Bayesian posterior probabilities (as percentages) are given at the nodes.

Partial sequences of the mitochondrial gene CO I were available for specimens from most of the clusters recognized by morphometry. A Bayesian tree (Fig 3) fully supported most of the morphospecies well classified by LDA of nest sample means with Bayesian posterior probabilities close to 1 (*T*. *ariadnae* sp.n., *T*. *artvinensis*, *T*. *flavicornis*, *T*. *helenae* sp.n., *T*. *lucidus* sp.n., *T*. *nylanderi*, *T*. *parvulus*, *T*. *schoedli*, *T*. *similis* sp.n,). In addition, the molecular data also substantiated *T*. *tergestinus*, less well supported by morphometry, as a separate lineage.

Molecular analysis reliably distinguished neither *T*. *laconicus* and *T*. *lichtensteini* nor *T*. *crasecundus* and *T*. *crassispinus*. This matches the slightly lower support of these taxa in the morphological analysis. The various specimens of *T*. *subtilis*, though with high classification rate by morphometry, did not form a distinct cluster in the molecular analysis. The placements of a worker from Bistrits, Bulgaria—morphologically identified as *T*. *crassispinus*–amidst specimens of *T*. *lichtensteini* and that of one worker of *T*. *subtilis* from Akseki, Turkey, near *T*. *schoedli* and *T*. *flavicornis* remains unexplained and might be due to an accidental exchange of specimens.

It was not aim of the study to provide a robust phylogeny of the here investigated taxa. The basal nodes of the tree show only very weak support. Nevertheless, our analysis supports the *T*. *nylanderi* complex, the *T*. *parvulus* complex, and the *T*. *lichtensteini* complex as monophyletic groups. As mentioned above, the two taxa of the *T*. *lichtensteini* complex clustered together but could not be distinguished reliably.

The *Temnothorax angustifrons* complex is not supported by the molecular data. Instead, *T*. *lucidus* sp.n. forms a weakly supported outgroup to the *T*. *nylanderi* complex, and *T*. *similis* sp.n. falls into the *T*. *parvulus* cluster. Similarly, the taxa of the *T*. *sordidulus* complex are scattered throughout the whole phylogenetic tree.

### Synopsis of East-Mediterranean representatives of *Temnothorax nylanderi* species-group

#### *Temnothorax angulinodis* complex

*Temnothorax angulinodis* **sp. n.**

#### *Temnothorax angustifrons* complex

*Temnothorax angustifrons* **sp. n.**

*Temnothorax lucidus* **sp. n.**

*Temnothorax similis* **sp. n.**

*Temnothorax subtilis* **sp. n.**

#### *Temnothorax flavicornis* complex

*Temnothorax flavicornis* (Emery, 1870)

#### *Temnothorax lichtensteini* complex

*Temnothorax lichtensteini* (Bondroit, 1918)

*Temnothorax laconicus* Csősz & al. 2014

#### *Temnothorax nylanderi* complex

*Temnothorax nylanderi* (Foerster, 1850)

*Temnothorax crassispinus* (Karavaiev, 1926)

*Temnothorax crasecundus* Seifert & Csősz, 2014

#### *Temnothorax parvulus* complex

*Temnothorax parvulus* (Schenck, 1852)

*Temnothorax ariadnae* **sp. n.**

*Temnothorax helenae* **sp. n.**

#### *Temnothorax sordidulus* complex

*Temnothorax artvinensis* Seifert, 2006

*Temnothorax schoedli* Seifert, 2006

*Temnothorax sordidulus* (Müller, 1923)

*Temnothorax carinthiacus* (Bernard, 1957)

*Temnothorax tergestinus* (Finzi, 1928) **stat.n.**

*Temnothorax saxonicus* (Seifert, 1995) **syn.n.**

### Key to worker caste

**Fig 4:**
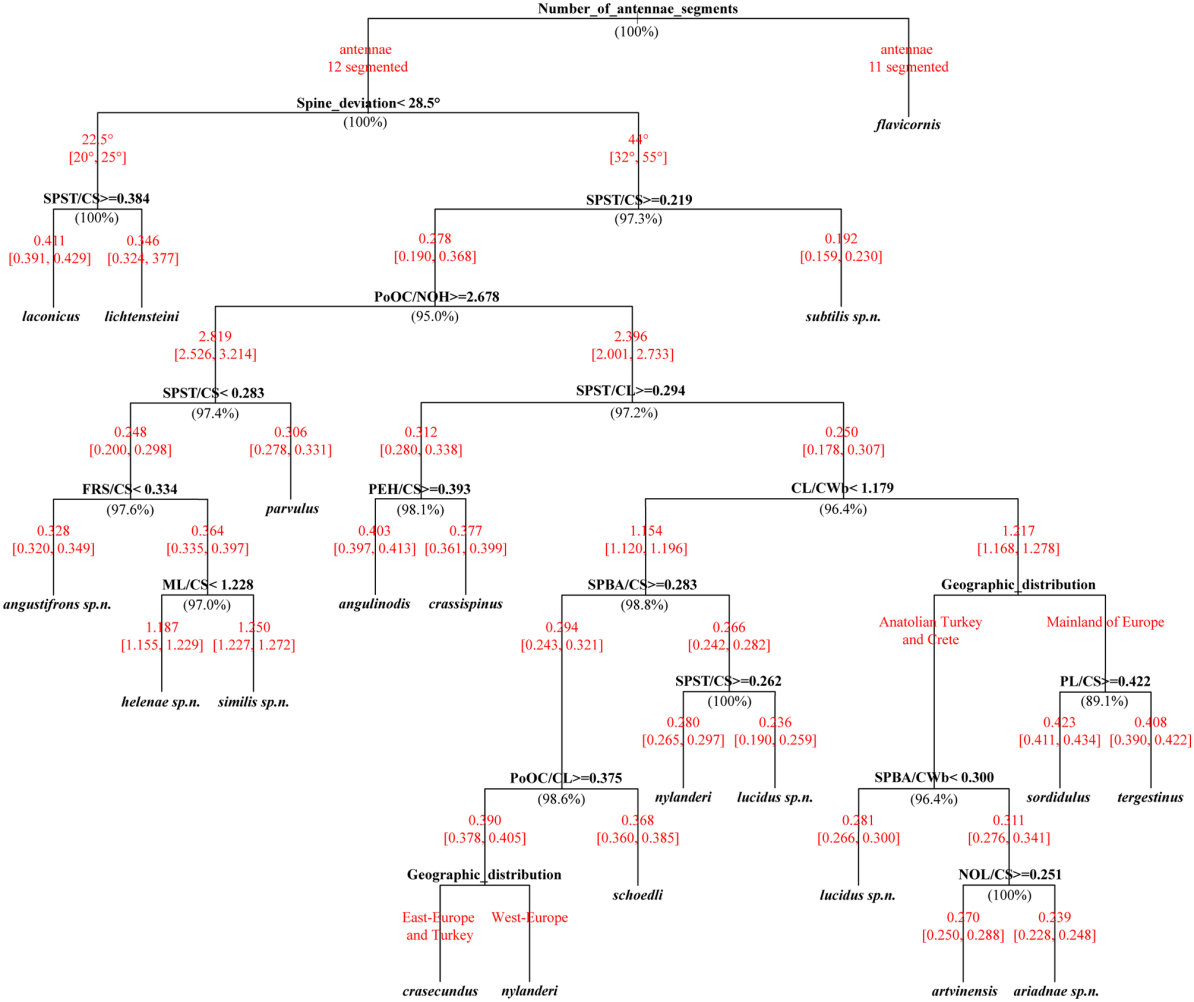
Decision Tree, an easy-to-use graphical display of a dichotomous identification key of workers of *Temnothorax nylanderi* species-group. The classification success in percentages is given in brackets under each particular node. Means and minimum, maximum values of morphometric ratios are given for each branch in red. At two positions (nodes) the originally suggested continuous morphometric traits were replaced by more reliable data about geographic distribution.

The following dichotomous identification key for the worker caste of the *Temnothorax nylanderi* species-group was generated by Decision Tree in R [41] based on continuous morphometric traits. It provides an easy-to-use identification guide, which is also displayed graphically in Fig 4. The reliability of characters has been tested and the percentages at each node indicate the classification success. The key is based on nest sample means (which require the investigation of 2 to 3 individuals from each nest). However, because the range of morphometric traits calculated from nest sample means is more or less identical with the 5–95% percentile range of the pool of single individuals, the key is also expected to work on single specimens with high probability (>95%). Additional information is available in differential diagnoses, which helps to identify the most problematic cases.

1. Antennomere count: 11. (Body color: yellow. South Europe, Central and East Mediterranean…*Temnothorax flavicornis* (Emery, 1870)

- Antennomere count: 12…2

2. Median anatomical line of propodeal spine angle value to Weber length in lateral view < 28.5 [20–25°] (Body color: brown. Longitudinal carinae on median region of frons count: present. Longitudinal carinae on medial region of frons shape: forked. Smooth median region on frons count: absent)…3

-Median anatomical line of propodeal spine angle value to Weber length in lateral view >30…4

3. Spine length vs. absolute cephalic size (SPST/CS) > 0.384 [0.391, 0.429]. (Peloponnese peninsula, Greece)…*Temnothorax laconicus* Csősz & al. 2014

- Spine length vs. absolute cephalic size (SPST/CS) < 0.384 [0.324, 0.377]. (South Europe and West Anatolia, Turkey)…*Temnothorax lichtensteini* (Bondroit, 1918)

4. Spine length vs. absolute cephalic size (SPST/CS) < 0.219 [0.159, 0.230]. (Body color: yellow. Smooth median region on frons count: present. Anatolia, Turkey)…*Temnothorax subtilis* sp. n.

- Spine length vs. absolute cephalic size (SPST/CS) > 0.219 [0.190, 0.368]…5

5. Postocular distance vs. Maximum height of the petiolar node (PoOC/NOH) > 2.678 [2.526, 3.214]…6

- Postocular distance vs. Maximum height of the petiolar node (PoOC/NOH) < 2.678 [2.001, 2.733]…9

6. Spine length vs. absolute cephalic size (SPST/CS) < 0.283 [0.278, 0.331]. (Yellow to brown species. Whole head dorsum homogenously areolate, dull. Europe and North Anatolia,

Turkey)…*Temnothorax parvulus* (Schenck, 1852)

- Spine length vs. absolute cephalic size (SPST/CS) > 0.283 [0.200, 0.298]. (Sculpture of head dorsum less homogenous: medially smooth to inconspicuously areolate ground sculpture often superimposed by costulae)…7

7. Frontal carina distance vs. absolute cephalic size (FRS/CS) < 0.334 [0.320, 0.349]. (Head dorsum smooth and shiny. Ground sculpture inconspicuously areolate, smooth and shiny superimposed by feeble costulae only. Anatolia, Turkey)…*Temnothorax angustifrons* sp. n.

- Frontal carina distance vs. absolute cephalic size (FRS/CS) < 0.334 [0.335, 0.397]…8

8. Mesosoma longer ML/CS > 1.228 [1.227, 1.272]. Brown species. Head dorsum smooth and shiny. Ground sculpture inconspicuously areolate, smooth and shiny superimposed by feeble costulae only South Anatolia, Turkey…*Temnothorax similis* sp. n.

- Mesosoma longer ML/CS < 1.228 [1.155, 1.229]. Yellow to light brown species. Whole head dorsum areolate superimposed be feeble costulae, occasionally narrow median strip inconspicuously areolate, shiny. Greek mainland, Crete and a single record known from North Anatolia, Turkey…*Temnothorax helenae* sp. n.

Note: Perfect separation of *T*. *helenae* sp. n. from *T*. *ariadnae* sp. n. may be difficult if only a single diagnostic feature is considered, more accurate option for separation is given under given in differential diagnosis under *T*. *ariadnae* sp. n.

9. Propodeal spines longer: SPST/CL > 0.294 [0.280, 0.338]…10

- Propodeal spines shorter: SPST/CL < 0.294 [0.178, 0.307]…11

10. Petiole higher: PEH/CS > 0.402 [0.397, 0.413]. In lateral view frontal profile of petiolar node meeting dorso-caudal plate in an acute angle (72–82°) with a sharp ridge. Endemic to Peloponnese peninsula, Greece…*Temnothorax angulinodis* sp. n.

- Petiole lower: PEH/CS < 0.380 [0.366, 0.407]. Petiolar node in lateral view with a concave frontal profile meeting truncate dorsum in an obtuse angle (100–115°) with a narrowly rounded transition, without a conspicuous sharp fronto-dorsal ridge on the petiolar node. Central-East Europe and the Balkans…*Temnothorax crassispinus* (Karavaiev, 1926).

Note: Separation of *T*. *crassispinus* from *T*. *crasecundus* may be difficult based on a single diagnostic feature, more accurate option for their separation is given in differential diagnosis of *T*. *crassispinus*.

11. Head considerably longer than broad: CL/CWb > 1.179 [1.168, 1.278] with straight sides…12

- Head shorter: CL/CWb < 1.179 [0.178, 0.307] sides remarkably convex…16

12. Known from mainland of Europe only…13

- Known from Anatolian Turkey and Crete Island…14

13. Petiole longer: PL/CS > 0.422 [0.411, 0.434]. Black species. Main sculpture on head dorsum coarse, longitudinally rugulose and/or carinulate, ground sculpture conspicuously areolate, always dull. Endemic to Dinaran Alps…*Temnothorax sordidulus* (Muüller, 1923) (see note under *T*. *tergestinus*)

- Petiole shorter: PL/CS < 0.422 [0.390, 0.422]. Dark brown to black species. Main sculpture on head dorsum coarse, longitudinally rugulose and/or carinulate, ground sculpture conspicuously areolate, always dull. Central and South Europe excluding Dinaran Alps…*Temnothorax tergestinus* (Finzi, 1928) stat.n.

Note: Perfect separation of this species from *T sordidulus* may be difficult if only a single diagnostic feature is considered. A more accurate way for separation is suggested in”differential diagnosis” under *T sordidulus*.

14. From dorsal view base of propodeal spines less distant: SPBA/CWb < 0.30 [0.266, 0.300].

Yellow species. Head dorsum smooth and shiny. Ground sculpture inconspicuously areolate, smooth and shiny superimposed by feeble costulae only. Anatolia, Turkey…*Temnothorax lucidus* sp. n.

- From dorsal view base of propodeal spines more distant: SPBA/CWb > 0.30 [0.276, 0.341]…15

15. Petiolar node longer: NOL/CS > 0.251 [0.250, 0.288]. Black species. Main sculpture on head dorsum coarse, longitudinally rugulose and/or carinulate, ground sculpture conspicuously areolate, always dull. North Anatolia, Turkey… *Temnothorax artvinensis* Seifert, 2006

-Petiolar node shorter: NOL/CS < 0.251 [0.228, 0.248]. Brown to dark brown species. Whole head dorsum uniformly areolate, narrow median strip occasionally inconspicuously areolate, shiny. Endemic to Crete…*Temnothorax ariadnae* sp. n. (see note under *T*. *helenae* sp. n.)

16. From dorsal view base of propodeal spines less distant: SPBA/CS < 0.283 [0.242, 0.282]…17

- From dorsal view base of propodeal spines more distant: SPBA/CS > 0.283 [0.243, 0.321]…18

17. Propodeal spines longer: SPST/CS > 0.262 [0.265, 0.297]. Yellow to brown species. Head dorsum with areolate ground sculpture always superimposed by parallel costulate main sculpture, dull. Central and West Europe: Italy, Austria, Germany and westward…*Temnothorax nylanderi* (Foerster, 1850)

- Propodeal spines shorter: SPST/CS < 0.262 [0.190, 0.259]. Yellow species. Head dorsum smooth and shiny. Ground sculpture inconspicuously areolate, smooth and shiny superimposed by feeble costulae only. Anatolia, Turkey…*Temnothorax lucidus* sp. n.

18. Postocular area shorter: PoOC/CL < 0.375 [0.360, 0.385]. Brown species. Main sculpture of head dorsum heterogeneous: on sides areolate ground sculpture always superimposed by parallel costulate main sculpture, dull, median part of head dorsum shiny. South-Central Anatolia, Turkey…*Temnothorax schoedli* Seifert, 2006

- Postocular area longer: PoOC/CL > 0.375 [0.378, 0.405] …19

19. Known from East Europe, the Balkans, Turkey and the Caucasus. Yellow to brown species. Whole head dorsum areolate superimposed by feeble costulae, occasionally narrow median strip inconspicuously areolate, shiny…*Temnothorax crasecundus* Seifert & Csősz, 2014

- Known from Central and West Europe: Italy, Austria, Germany and further west. Yellow to brown species. Head dorsum with areolate ground sculpture always superimposed by parallel costulate main sculpture, dull…*Temnothorax nylanderi* (Foerster, 1850)

### Descriptions and redefinitions of species and species-complexes

The description and redefinition of species belonging to the Ponto-Mediterranean *Temnothorax nylanderi* species-group can be found following the diagnoses of species complexes to which they belong. Species complexes are defined based on morphological similarities, but not fully supported by molecular (mtDNA) data. Patterns formerly recognized by NC-clustering were tested by confirmatory Linear Discriminant Analysis (Steps 2 and 3). The results of complex-wise analyses (Step 3) are displayed in scatterplots for each species-complex defined below.

Recent taxonomic papers on *Temnothorax* taxa [33], [48], [39] were considered in preparing descriptions and redefinitions of the species treated in this work. The varying degree of inclination of pilosity follows [53]. Definitions of surface sculpturing are linked to [54].

Definitions of species complexes as well as descriptions of species belonging to a certain complex are given in alphabetic order.

Body size is given in μm and morphometric ratios are given as means. In the differential diagnosis, we give the means of discriminant scores, minimum and maximum, and 5–95% percentiles to prevent that abnormal cases are misidentified. Nest sample means of ratios of morphometric variables are given in S4 Table for each species with standard deviation in italics followed by minimum and maximum in parentheses. In the diagnosis of species complexes, minimum and maximum values of body ratios are indicated.

### Diagnosis of *Temnothorax angulinodis* species-complex

**Fig 5:**
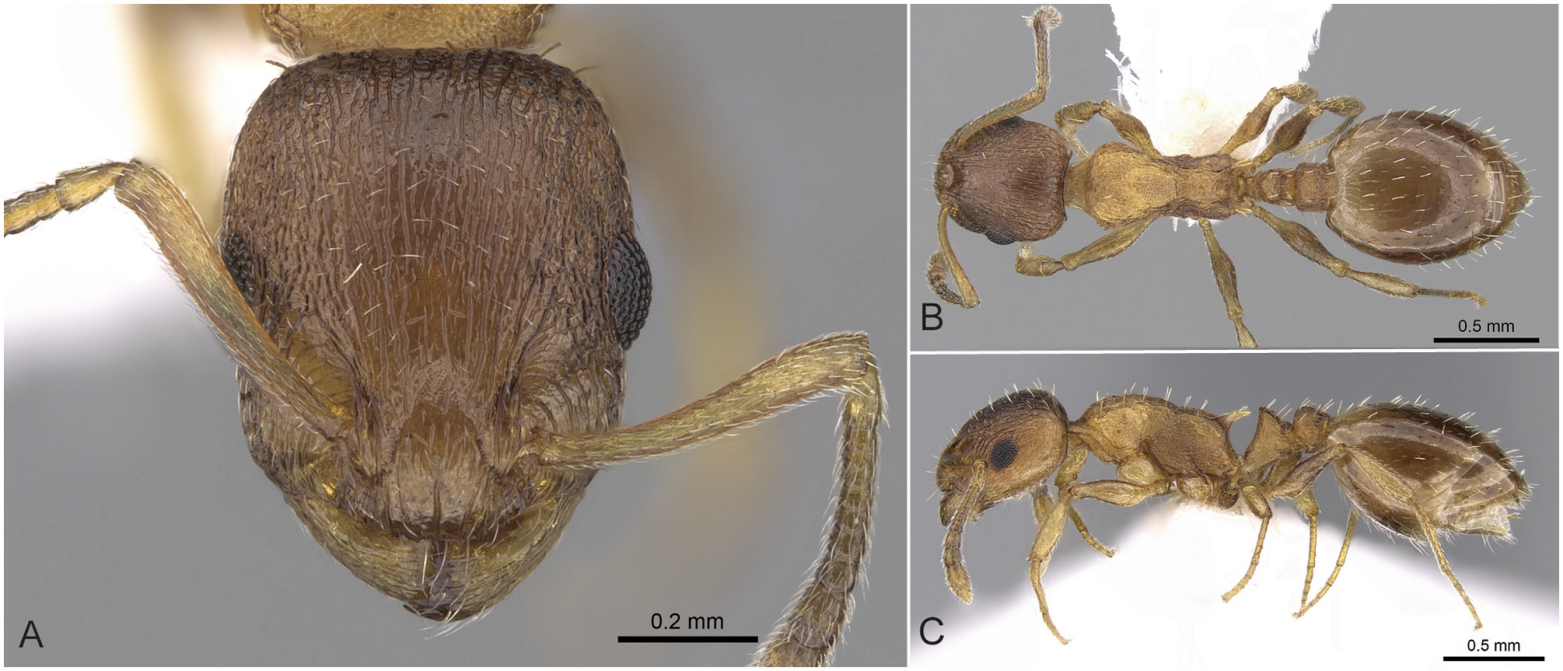
*Temnothorax angulinodis* sp.n. paratype worker. Head of paratype worker (CASENT0914688) in full face view (A), dorsal view of the body (B), lateral view of the body (C).

Workers of the *Temnothorax angulinodis* species-complex can be distinguished from those of other complexes treated in this revision by the combination of the following salient features: brown to black color; longer than broad head (CL/CWb [1.171, 1.222]), sculpture of head dorsum shiny: with inconspicuously areolate ground sculpture combined with parallel costulate main sculpture (Fig 5A); petiolar node in lateral view with a straight or weakly concave frontal profile meeting dorso-caudal plate in an acute angle (72–82°) with a sharp ridge, in dorsal view appearing as conspicuous semicircular anterior-lateral rim (Fig 5B); long propodeal spines (SPST/CS [0.332, 0.369]), deviating from longitudinal axis of mesosoma by 32–38° (Fig 5C).

This peculiar species-complex consists of a single species, *Temnothorax angulinodis* sp. n., which is only known from the Peloponnese peninsula. This species is clearly defined by NC-clustering and corroborated by LDA (Fig 2, S5 Table).

### *Temnothorax angulinodis* sp. n.

urn:lsid:zoobank.org:act:72F3B6B9-13DB-49D5-BBC8-CD2BA16E1CC9

#### Etymology

This name „*angulinodis*” refers to the sharply angulate profile of the petiolar node of this species.

#### Type material investigated

**Holotype worker labelled:** Greece_27 Peloponnesus, 22 km NNW Tripolis, Menalo Oros, 37.6539N, 22.2676E, 1500-1700mH, 29.IV.2011, leg. A. Schulz „256”, (HNHM), [**GRE:Tripolis-22NNW-20110429-256]**;

**Paratypes:** Greece_27, Peloponnesus, 22 km NNW Tripolis, Menalo Oros, 37.6539N, 22.2676E, 1500-1700mH, 29.IV.2011, leg. A. Schulz „256”, (2## HNHM, 1# CAS CASENT0906029; CASENT0914688), [**GRE:Tripolis-22NNW-20110429-256]**; Greece, Peloponnesus, Melanos Oros, 20 km NNW. Tripolis, 5 km SW. Levidion, 37.6607N, 22.2546E, 1700mH, 03.06.1994, leg. A. Schulz „1380”, (3## HNHM), [**GRE:Levidion-5SW-19940603-1380]**; Greece, Peloponnesus, Melanos Oros, 20 km NNW. Tripolis, 5 km SW. Levidion, 37.6607N, 22.2546E, 1700mH, 03.06.1994, leg. A. Schulz „1383” (2## HNHM), [**GRE:Levidion-5SW-19940603-1383]**;

The list of 15 non-type individuals belonging to 5 nest samples of other material investigated is given in S1 Table.

#### Worker (Fig 5A–5C, S1 Table, S4 Table)

Body color: brown. Body color pattern: mesosoma, antenna and legs excluding femora, waist and anterior region of 1st gastral tergite lighter than head, femora and posterior region of gaster. Absolute cephalic size: 594–657 μm (mean = 626, n = 8). Cephalic length vs. Maximum width of head capsule (CL/CWb): 1.171–1.222 (mean = 1.203). Postocular distance vs. cephalic length (PoOc/CL): 0.356–0.378 (mean = 0.368). Eye length vs. absolute cephalic size (EL/CS): 0.257–0.274 (mean = 0.265). Frontal carina distance vs. absolute cephalic size (FRS/CS): 0.366–0.388 (mean = 0.374). Median region of antennal rim vs. frontal carina in frontal view: not fully overlapped by frontal carina. Concentric carinae lateral to antennal foramen count: present. Carinae on medial region of frons shape: branched. Smooth median region on frons count: absent. Longitudinal carinae on median region of frons count: present. Median carina of clypeus count: present. Lateral carinae of clypeus count: present. Sculpture of submedian area of clypeus: smooth. Gena sculpture: rugoso-reticulate with feeble areolate ground sculpture. Gena frontal view shape: feebly convex. Genae contour from anterior view orientation: converging. Postocular sides of cranium contour frontal view orientation: converging posteriorly. Postocular side of cranium shape: feebly convex. Vertex sculpture: main sculpture homogenously forked costate, ground sculpture areolate; main sculpture dispersed forked costate sculpture, ground sculpture areolate. Posterior margin of vertex in frontal view shape: straight. Antennomere count: 12. Scape length vs. absolute cephalic size (SL/CS): 0.797–0.816 (mean = 0.808). Facial area of the scape absolute setal angle: 0–15°. External area of the scape absolute setal angle: 30°. Maximum mesosoma width vs. absolute cephalic size (MW/CS): 0.610–0.636 (mean = 0.621). Lateral region of pronotum sculpture: areolate ground sculpture, main sculpture forked costate. Dorsal region of mesosoma sculpture: rugulose with areolate ground sculpture. Metanotal depression count: present. Metanotal depression shape: shallow. Mesopleuron sculpture: areolate ground sculpture superimposed by dispersed rugulae. Metapleuron sculpture: areolate ground sculpture superimposed by dispersed rugulae. Spine length vs. absolute cephalic size (SPST/CS): 0.332–0.369 (mean = 355). Median anatomical line of propodeal spine vs. to Weber length angle value in lateral view: 32–38°. Apical spine distance vs. absolute cephalic size (SPTI/CS): 0.366–0.398 (mean = 0.386). Maximum spine distance vs. absolute cephalic size (SPWI/CS): 0.390–0.419 (mean = 0.409). Minimum spine distance vs. absolute cephalic size (SPBA/CS): 0.262–0.304 (mean = 0.285). Anterodorsal rim of petiole count: present. Anterodorsal edge of petiole dorsal view shape: semicircular. Truncate dorsum of petiolar node count: absent. Truncate dorsum dorsal side contour lateral view: absent. Frontal profile of petiolar node in lateral view shape: straight. Anterodorsal edge of petiole count: present. Anterodorsal edge of petiole angle value: 72–82°. Dorsal region of petiole sculpture: ground sculpture areolate, main sculpture dispersed costulate. Posterodorsal edge of petiole count: absent. Caudal petiolar profile shape: straight; convex. Caudal petiolar profile angle value to ventral margin of petiole: more than 80°. Dorsal region of postpetiole sculpture: ground sculpture areolate, main sculpture dispersed costulate.

#### Differential diagnosis

Due to the unique combination of the long spine, high petiole, and sharp transversal crest on the dorsum of the petiolar node this species is easily distinguishable from related taxa even by simple visual inspection.

#### Geographic distribution

This species is known only from the Peloponnese peninsula.

### Diagnosis of *Temnothorax angustifrons* species-complex

Workers of *the Temnothorax angustifrons* species-complex can be distinguished from those of other complexes by the combination of the following salient features: light yellow to light brown color; moderately longer than broad head (CL/CWb [1.135, 1.254]), sculpture of head dorsum shiny, with inconspicuously areolate ground sculpture combined with feeble costulate main sculpture; short to moderately long propodeal spines (SPST/CS [0.159, 0.267]), deviating from longitudinal axis of mesosoma by 47–52°; petiolar node in lateral view with a weakly concave frontal profile meeting dorso-caudal plate in an obtuse angle (95–105°) with a moderately sharp ridge, in dorsal view appearing as a visible (occasionally inconspicuous) anterior-lateral rim.

**Fig 6:**
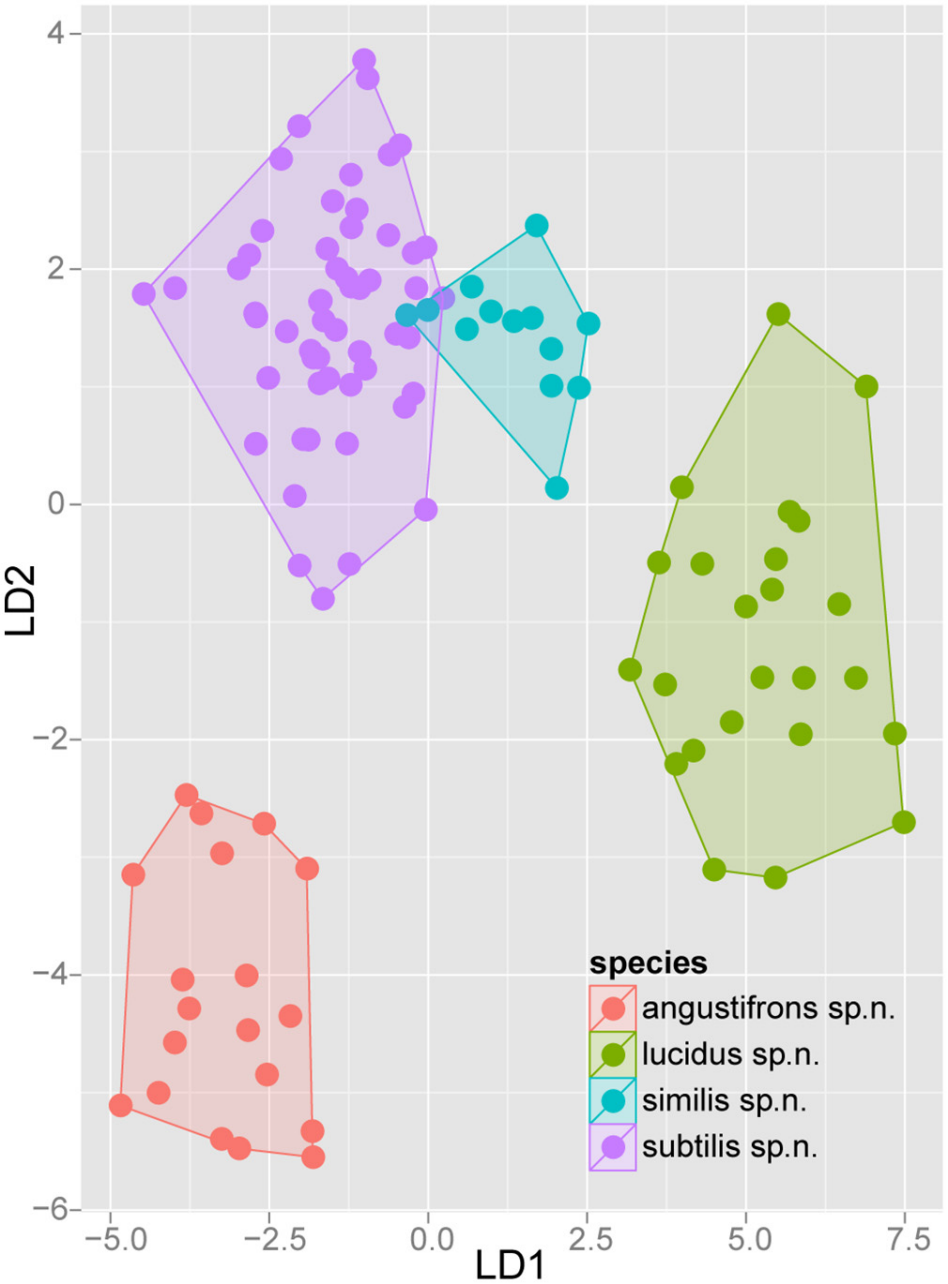
Scatterplot of discriminant scores for *Temnothorax angustifrons* species-complex is illustrated on the 1^st^ and 2^nd^ axis. Color codes: *T*. *angustifrons* sp.n. (red), *T*. *lucidus* sp.n. (green), *T*. *similis* sp.n. (blue) and *T*. *subtilis* sp.n. (lilla). Convex hulls visualize the range for each group.

**Fig 7:**
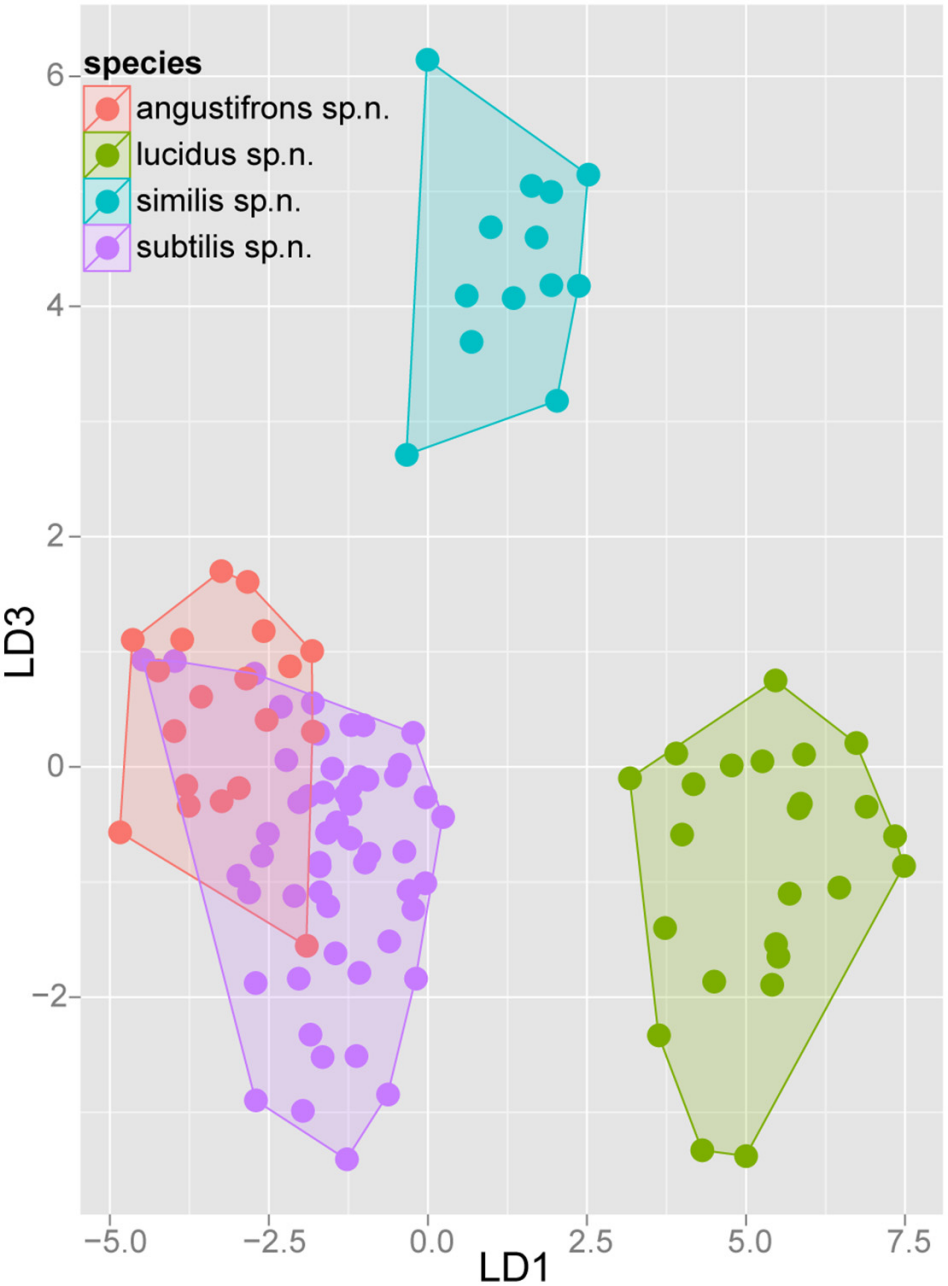
Scatterplot of discriminant scores for *Temnothorax angustifrons* species-complex is illustrated on the 1^st^ and 3^rd^ axis. Color codes: *T*. *angustifrons* sp.n. (red), *T*. *lucidus* sp.n. (green), *T*. *similis* sp.n. (blue) and *T*. *subtilis* sp.n. (lilla). Convex hulls visualize the range for each group.

**Fig 8:**
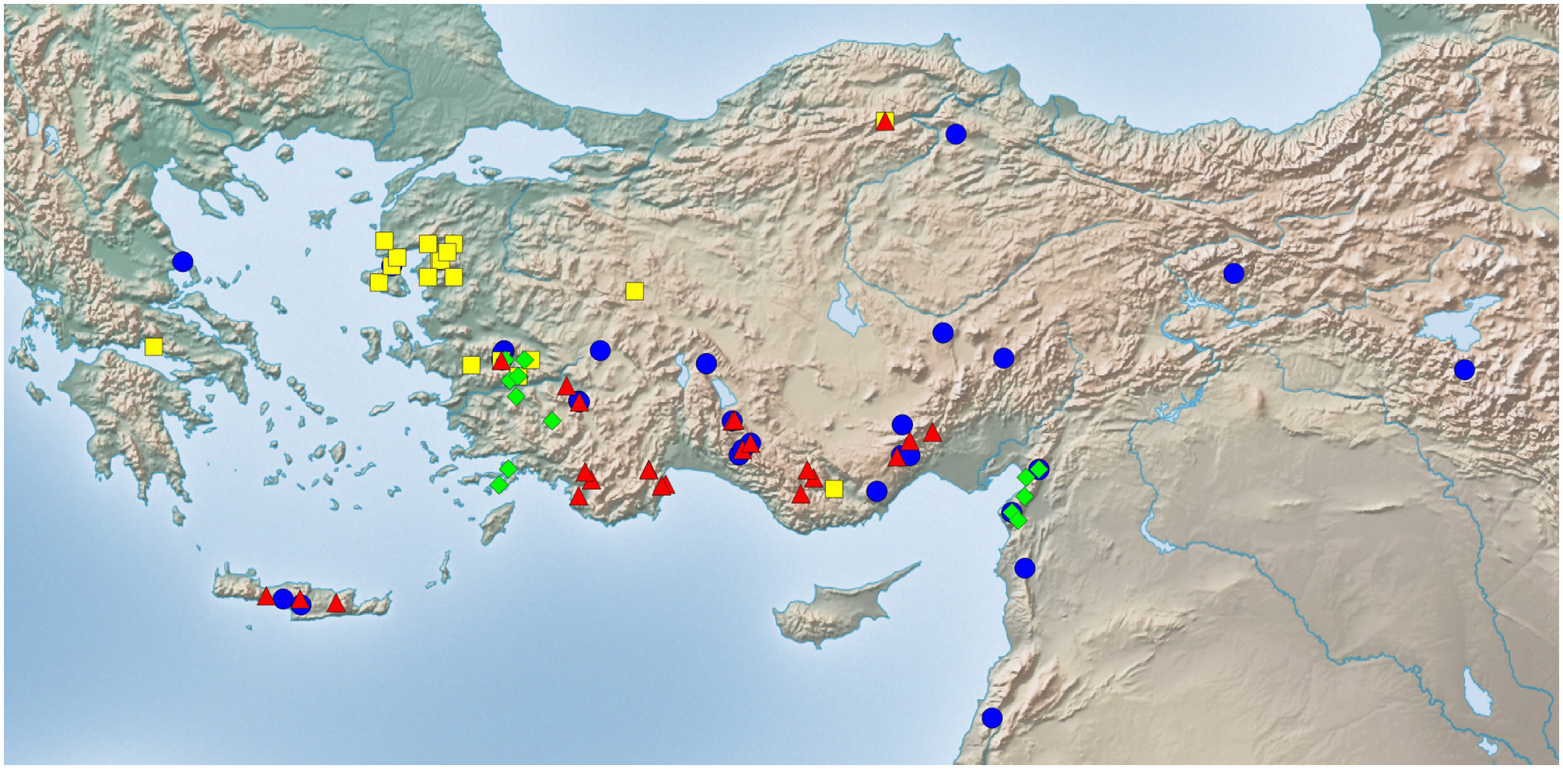
Sampling sites of *Temnothorax angustifrons* species-complex. Color codes: *T*. *angustifrons* sp.n. (yellow rectangles), *T*. *lucidus* sp.n. (red triangles), *T*. *similis* sp.n. (green diamonds) and *T*. *subtilis* sp.n. (blue circles).

Exploratory NC-clustering (Fig 2) revealed the existence of four species (*Temnothorax angustifrons* sp. n., *T*. *lucidus* sp. n., *T*. *similis* sp. n., *T*. *subtilis* sp. n.) within this complex, which was confirmed by LDA (S5 Table, Figs 6 and 7). Members of this species-complex are known to occur in Turkey and Crete. The occurrence of two samples found in Greece may be ascribed to anthropochory, i.e., dispersal by human activities (Fig 8).

### *Temnothorax angustifrons* sp. n.

urn:lsid:zoobank.org:act:CD811D21-6148-45DC-88A9-68AF8011CDC6

#### Etymology

The species name refers to the frons, which is narrow (Latin: angustus *m*) relative to other *Temnothorax* species treated in this revision.

#### Type material investigated

**Holotype worker labelled:** Turkey, Road Edremit to Kalkun, Kaz Daği Mountain, 39.411 N, 27.093 E, 752mH, 10.05.2003, leg. A. Schulz, „072” (HNHM CASENT0914693), [**TUR:Edremit-Kalkun-20030510-072**];

**Paratypes:** Turkey, Road Edremit to Kalkun, Kaz Daği Mountain, 39.411 N, 27.093 E, 752mH, 10.05.2003, leg. A. Schulz, „072” (1# HNHM), [**TUR:Edremit-Kalkun-20030510-072**]; Turkey, Road Edremit to Kalkun, Kaz Daği Mountain, 39.411 N, 27.093 E, 752mH, 10.05.2003, leg. A. Schulz, „076” (2## HNHM), [**TUR:Edremit-Kalkun-20030510-076**];

The list of 59 non-type individuals belonging to 17 nest samples of other material investigated is given in S1 Table.

#### Worker (Fig 9A–9C, S1 Table, S4 Table)

Body color: yellow. Body color pattern: mesosoma, antenna and legs, waist and anterior region of 1st gastral tergite lighter than head dorsum and posterior region of gaster. Antennomere count: 12. Antenna color pattern: clava concolorous funicle. Absolute cephalic size: 500–590 μm (mean = 546, n = 16). Cephalic length vs. Maximum width of head capsule (CL/CWb): 1.193–1.254 (mean = 1.224). Postocular distance vs. cephalic length (PoOc/CL): 0.377–0.401 (mean = 0.389). Postocular sides of cranium contour frontal view orientation: converging posteriorly. Postocular sides of cranium contour frontal view shape: convex. Vertex contour line in frontal view shape: straight. Vertex sculpture: main sculpture dispersed forked costate, ground sculpture inconspicuous areolate. Genae contour from anterior view orientation: converging. Gena contour line in frontal view shape: feebly convex. Gena sculpture: rugoso-reticulate with feeble areolate ground sculpture. Median region of antennal rim vs. frontal carina in frontal view structure: not fully overlapped by frontal carina. Concentric carinae laterally surrounding antennal foramen count: present. Eye length vs. absolute cephalic size (EL/CS): 0.247–0.263 (mean = 0.254). Frontal carina distance vs. absolute cephalic size (FRS/CS): 0.320–0.349 (mean = 0.328). Longitudinal carinae on median region of frons count: present; absent. Longitudinal carinae on medial region of frons shape: forked. Smooth median region on frons count: present. Scape length vs. absolute cephalic size (SL/CS): 0.807–0.832 (mean = 0.821). Facial area of the scape absolute setal angle: 0–15°. External area of the scape absolute setal angle: 30°. Ground sculpture of submedian area of clypeus: smooth. Median carina of clypeus count: present. Lateral carinae of clypeus count: present. Median anatomical line of propodeal spine angle value to Weber length in lateral view: 50–55°. Spine length vs. absolute cephalic size (SPST/CS): 0.200–0.264 (mean = 0.231). Minimum spine distance vs. absolute cephalic size (SPBA/CS): 0.242–0.275 (mean = 0.259). Maximum spine distance vs. absolute cephalic size (SPWI/CS): 0.282–0.319 (mean = 0.298). Apical spine distance vs. absolute cephalic size (SPTI/CS): 0.265–0.298 (mean = 0.284). Maximum mesosoma width vs. absolute cephalic size (MW/CS): 0.588–0.620 (mean = 0.601). Metanotal depression count: present. Metanotal depression shape: deep. Dorsal region of mesosoma sculpture: areolate ground sculpture, superimposed by dispersed rugae. Lateral region of pronotum sculpture: areolate ground sculpture, main sculpture dispersed costate. Mesopleuron sculpture: areolate ground sculpture superimposed by dispersed rugulae. Metapleuron sculpture: areolate ground sculpture superimposed by dispersed rugulae. Frontal profile of petiolar node contour line in lateral view shape: straight; concave. Dorsal profile of petiolar node contour line angle value to frontal profile of petiole contour line in lateral view: 90–100°. Anterodorsal rim of petiole count: absent medially. Dorsal region of petiole sculpture: ground sculpture areolate, main sculpture dispersed rugose; ground sculpture areolate, main sculpture absent. Dorso-caudal petiolar profile contour line in lateral view shape: straight; concave. Dorsal region of postpetiole sculpture: ground sculpture areolate, main sculpture dispersed rugose; ground sculpture areolate, main sculpture absent.

**Fig 9:**
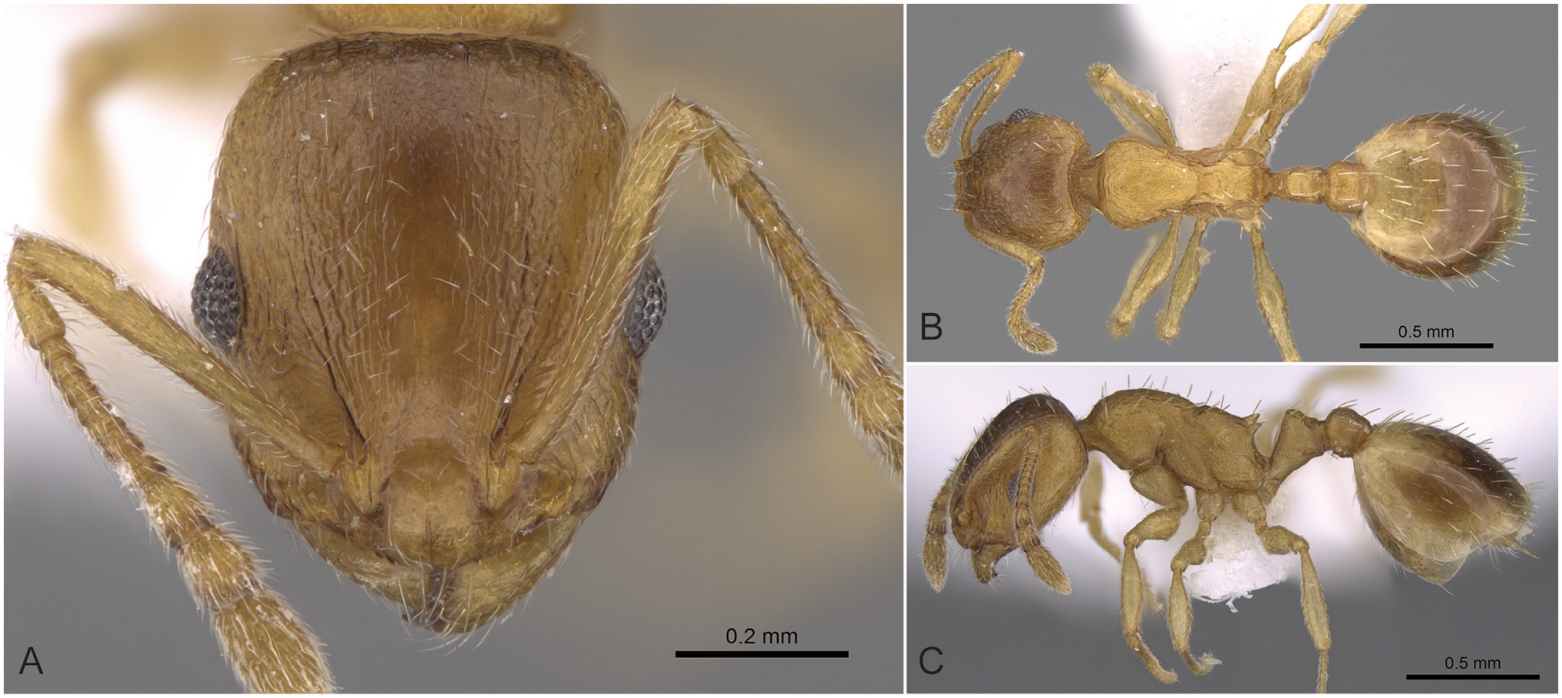
*Temnothorax angustifrons* sp.n. holotype worker. Head of the holotype worker (CASENT0914693) in full face view (A), dorsal view of the body (B), lateral view of the body (C).

#### Differential diagnosis

This species has the longest scape (SL/CS) and the narrowest frons (FRS/CS) of all species treated in this revision (S5 Table). *Temnothorax angustifrons* sp. n. strikingly resembles other members of this complex in Turkey. Therefore, the above specified characters may slightly overlap, but a simple ratio of their combination (FRS/SL) provides an excellent tool to separate this species from its relatives with an error rate of less than 5% for single individuals:

*T*. *angustifrons* sp.n. (n 67) = 0.398 [0.374, 0.439], [5–95% percentiles: 0.381, 0.422]

Combined pool of *T*. *lucidus* sp.n., *T*. *similis* sp.n. and *T*. *subtilis* sp.n. (n 284) = 0.461 [0.400, 0.512], [5–95% percentiles: 0.432, 0.489]

Simple ratios of the above-mentioned morphometric traits (SL/CS and FRS/CS) also separate *T*. *angustifrons* sp. n. from other species belonging to other species complexes in Turkey (S5 Table).

#### Geographic distribution

This species is known from Western Anatolia, Turkey (Fig 8). Its occurrence in Stenos, Greece, might be ascribed to anthropochory.

### *Temnothorax lucidus* sp. n.

urn:lsid:zoobank.org:act:3C96E879-7FB4-4D94-8AC7-805CE9EF187F

#### Etymology

The species epithet “lucidus” refers to the shiny light (Latin: lūcidus *m*) yellow surface sculpturing of the worker caste.

#### Type material investigated

**Holotype worker labelled:** TUR:492 Turkey, Taurus Mt., 3 km W. Arslanköy, 37.0024 N, 34.2151 E, 1900mH, 06.11.2011, leg. A. Schulz, (HNHM, CASENT0914692), [**TUR:Arslanköy-3W-20111106-492**];

**Paratypes:** TUR:492 Turkey, Taurus Mt., 3 km W. Arslanköy, 37.0024 N, 34.2151 E, 1900mH, 06.11.2011, leg. A. Schulz, (1# HNHM, 2## CAS), [**TUR:Arslanköy-3W-20111106-492**]; TUR:493 Turkey, Taurus Mt., 3 km W. Arslanköy, 37.0024 N, 34.2151 E, 1900mH, 06.11.2011, leg. A. Schulz, (3## HNHM, 2## CAS), [**TUR:Arslanköy-3W-20111106-493**];

The list of 65 non-type individuals belonging to 22 nest samples of other material investigated is given in S1 Table.

#### Worker (Fig 10A–10C, S1 Table, S4 Table)

Body color: yellow. Body color pattern: mesosoma, antenna and legs, waist and anterior region of 1st gastral tergite lighter than head dorsum and posterior region of gaster. Antenna color pattern: clava concolorous funicle. Absolute cephalic size: 560–670 μm (mean = 618, n = 24). Cephalic length vs. Maximum width of head capsule (CL/CWb): 1.139–1.221 (mean = 1.173). Postocular distance vs. cephalic length (PoOc/CL): 0.368–0.400 (mean = 0.380). Postocular sides of cranium contour frontal view orientation: converging posteriorly. Postocular sides of cranium contour frontal view shape: convex. Vertex contour line in frontal view shape: straight. Vertex sculpture: main sculpture dispersed forked costate, ground sculpture inconspicuous areolate. Genae contour from anterior view orientation: converging. Gena contour line in frontal view shape: feebly convex. Gena sculpture: rugoso-reticulate with feeble areolate ground sculpture. Median region of antennal rim vs. frontal carina in frontal view structure: not fully overlapped by frontal carina. Concentric carinae laterally surrounding antennal foramen count: present. Eye length vs. absolute cephalic size (EL/CS): 0.247–0.276 (mean = 0.262). Frontal carina distance vs. absolute cephalic size (FRS/CS): 0.356–0.389 (mean = 0.370). Longitudinal carinae on median region of frons count: present; absent. Longitudinal carinae on medial region of frons shape: forked. Smooth median region on frons count: present. Antennomere count: 12. Scape length vs. absolute cephalic size (SL/CS): 0.767–0.832 (mean = 0.796). Facial area of the scape absolute setal angle: 0–15°. External area of the scape absolute setal angle: 30°. Ground sculpture of submedian area of clypeus: smooth. Median carina of clypeus count: present. Lateral carinae of clypeus count: present. Median anatomical line of propodeal spine angle value to Weber length in lateral view: 45–50°. Spine length vs. absolute cephalic size (SPST/CS): 0.190–0.259 (mean = 0.236). Minimum spine distance vs. absolute cephalic size (SPBA/CS): 0.241–0.280 (mean = 0.258). Maximum spine distance vs. absolute cephalic size (SPWI/CS): 0.272–0.339 (mean = 0.304). Apical spine distance vs. absolute cephalic size (SPTI/CS): 0.261–0.318 (mean = 0.290). Maximum mesosoma width vs. absolute cephalic size (MW/CS): 0.599–0.636 (mean = 0.621). Metanotal depression count: present. Metanotal depression shape: deep. Dorsal region of mesosoma sculpture: fine areolate ground sculpture, superimposed by dispersed rugae. Lateral region of pronotum sculpture: inconspicuous areolate ground sculpture, main sculpture dispersed costate. Mesopleuron sculpture: fine areolate ground sculpture, superimposed by dispersed rugulae. Metapleuron sculpture: fine areolate ground sculpture, superimposed by dispersed rugulae. Frontal profile of petiolar node contour line in lateral view shape: straight; concave. Dorsal profile of petiolar node contour line angle value to frontal profile of petiole contour line in lateral view: 95–100°. Anterodorsal rim of petiole count: absent medially. Dorsal region of petiole sculpture: ground sculpture areolate, main sculpture dispersed rugose; ground sculpture areolate, main sculpture absent. Dorso-caudal petiolar profile contour line in lateral view shape: straight; concave. Dorsal region of postpetiole sculpture: ground sculpture areolate, main sculpture dispersed rugose; ground sculpture areolate, main sculpture absent.

**Fig 10:**
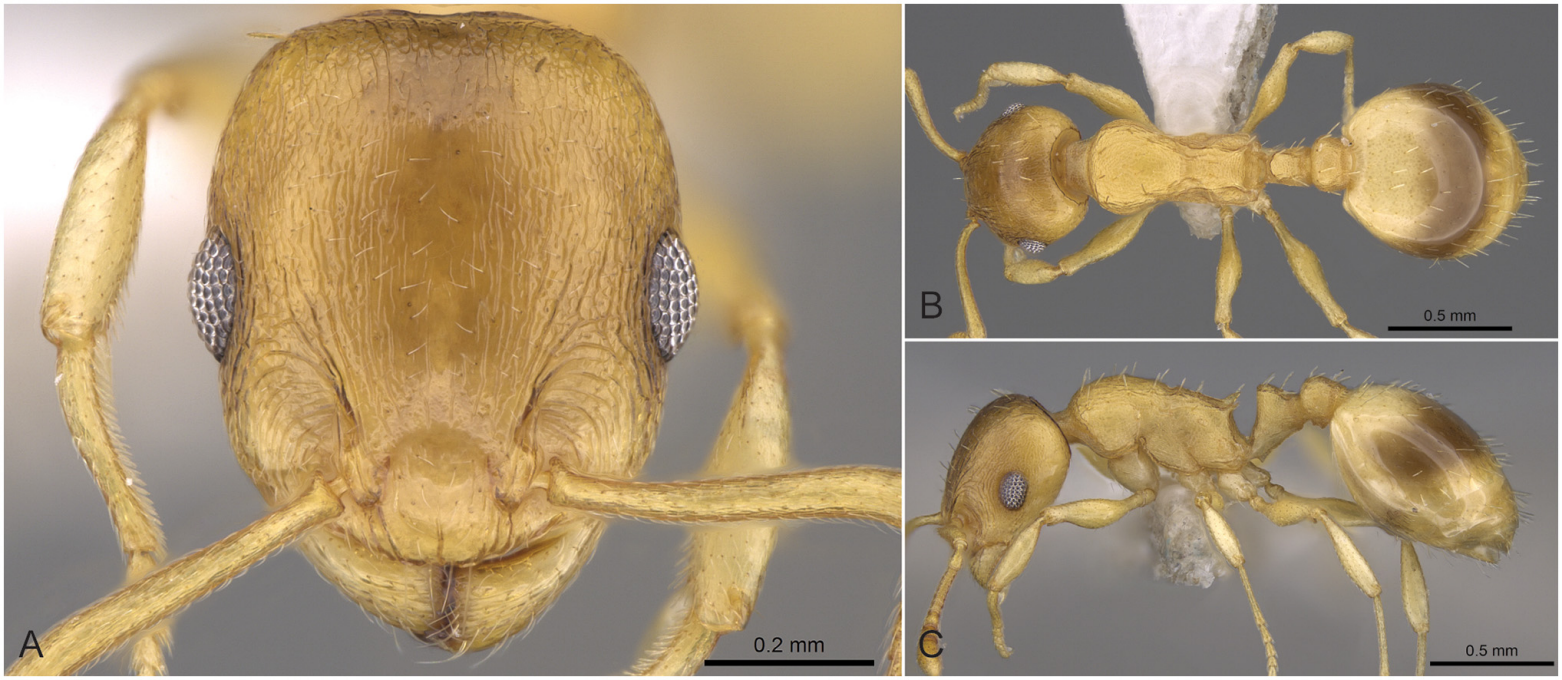
*Temnothorax lucidus* sp.n. holotype worker. Head of the holotype worker (CASENT0914692) in full face view (A), dorsal view of the body (B), lateral view of the body (C).

#### Differential diagnosis

This species differs from members of other species complexes treated in this revision by its smooth and shiny head dorsum and the relatively short propodeal spines. This character combination is shared with other species belonging to *T*. *angustifrons* complex: *T*. *angustifrons* sp. n., *T*. *subtilis* sp. n. and *T*. *similis* sp. n. Nest samples of *T*. *lucidus* sp. n. can be separated from those of *T*. *angustifrons* sp. n. by their shorter scape (SL/CS) and wider frons (FRS/CS). Their simple ratio (FRS/SL) provides an excellent tool to separate workers with less than 5% of error rate (see details under differential diagnosis under *T*. *angustifrons* sp. n.). Ratios of NOH/CS and PEH/CS help to distinguish this species from *T*. *similis* sp. n. on the level of nest samples. A discriminant (D4) function, including discriminant scores for separating single individuals with acceptably low error rate, is given in the differential diagnosis of the latter.

The spine length ratio (SPST/CS, see S4 Table) provides a good quick character to separate nest samples of *T*. *lucidus* sp. n. from those of *T*. *subtilis* sp. n., but in single workers this character may broadly overlap between the two species. A discriminant function with reduced character number (D4) yields 98.6% classification success rate (see details in differential diagnosis under *T*. *subtilis* sp. n.).

*Temnothorax lucidus* sp. n. can be easily separated from two additional species of the *T*. *parvulus* complex that occur in Crete, *T*. *ariadnae* sp. n. and *T*. *helenae* sp. n. based on shiny surface of the head dorsum. In exceptional cases, or if dust obstructs a clear view of the surface sculpture, nest samples of *T*. *lucidus* sp. n. can be separeted from *T*. *ariadnae* sp. n. by non-overlapping ranges of body ratios (NodL/CS and NOL/CS, see S4 Table). Simple ratios do not help to dinstguish *T*. *lucidus* sp. n. and *T*. *helenae* sp. n., hence a simplified discriminant function (D3 = -0.0807*POC +0.0896*SL -0.0578*SPTI -11.284) is recommended to separate nest samples of these specie. The same function yields 97.9% classification success rate between single individuals of these species.

D3 scores for single individuals:

*T*. *helenae* sp. n. (n 169) = -1.982 [-4.509, +0.876], [5–95% percentiles: -3.433, -0.296]

*T*. *lucidus* sp. n. (n 70) = +1.982 [-0.101, +3.944], [5–95% percentiles: +0.564, +3.559]

#### Geographic distribution

This species is known from South and Central Anatolia, Turkey, and Crete (Fig 8).

### *Temnothorax similis* sp. n.

urn:lsid:zoobank.org:act:3BED5669-17A6-479B-9236-DA4FA22E2180

#### Etymology

Species epithet (Latin: similis *m*, *f* = similar) refers to the superficial similarity of this species to *T*. *schoedli*.

#### Type material investigated

**Holotype worker labelled:** TUR:512 Turkey, Nur Dağlari, 22 km NW. Antakya, 36.3050 N, 36.0098 E, 1600-1800mH, 07.11.2011, leg. A. Schulz, (HNHM, CASENT0914691), [**TUR:Antakya-22NW-20111107-512**];

**Paratypes:** TUR:512 Turkey, Nur Dağlari, 22 km NW. Antakya, 36.3050 N, 36.0098 E, 1600-1800mH, 07.11.2011, leg. A. Schulz, (2## HNHM), [**TUR:Antakya-22NW-20111107-512**]; TUR:515 Turkey, Nur Dağlari, 22 km NW. Antakya, 36.3050 N, 36.0098 E, 1600-1800mH, 07.11.2011, leg. A. Schulz, (4## HNHM), [**TUR:Antakya-22NW-20111107-515**]; TUR:520 Turkey, Nur Dağlari, 22 km NW. Antakya, 36.3050 N, 36.0098 E, 1600-1800mH, 07.11.2011, leg. A. Schulz, (3## HNHM, 1# CAS), [**TUR:Antakya-22NW-20111107-520**]; TUR:547 Turkey, Nur Dağlari, 10 km NW. Hassa, 36.8459 N, 36.4330 E, 1500mH, 08.11.2011, leg. A. Schulz, (4## HNHM), [**TUR:Hassa-10NW-20111108-547**]; TUR:552 Turkey, Nur Dağlari, 10 km NW. Hassa, 36.8459 N, 36.4330 E, 1500mH, 08.11.2011, leg. A. Schulz, (3## HNHM, 2## CAS), [**TUR:Hassa-10NW-20111108-552**];

The list of 23 non-type individuals belonging to 8 nest samples of other material investigated is given in S1 Table.

#### Worker (Fig 11A–11C, S1 Table, S4 Table)

Body color: yellow; brown. Body color pattern: mesosoma, antenna and legs, waist and anterior region of 1st gastral tergite lighter than head dorsum and posterior region of gaster. Antenna color pattern: clava concolorous funicle. Absolute cephalic size: 505–635 μm (m = 571, nlot = 13). Cephalic length vs. Maximum width of head capsule (CL/CWb): 1.145–1.233 (m = 1.183). Postocular distance vs. cephalic length (PoOc/CL): 0.371–0.398 (m = 0.386). Postocular sides of cranium contour frontal view orientation: converging posteriorly. Postocular sides of cranium contour frontal view shape: convex. Vertex contour line in frontal view shape: straight. Vertex sculpture: main sculpture dispersed forked costate, ground sculpture inconspicuous areolate. Genae contour from anterior view orientation: converging. Gena contour line in frontal view shape: feebly convex. Gena sculpture: rugoso-reticulate with feeble areolate ground sculpture. Median region of antennal rim vs. frontal carina in frontal view structure: not fully overlapped by frontal carina. Concentric carinae laterally surrounding antennal foramen count: present. Eye length vs. absolute cephalic size (EL/CS): 0.245–0.270 (m = 0.263). Frontal carina distance vs. absolute cephalic size (FRS/CS): 0.352–0.369 (m = 0.361). Longitudinal carinae on median region of frons count: present; absent. Longitudinal carinae on medial region of frons shape: forked. Smooth median region on frons count: present. Antennomere count: 12. Scape length vs. absolute cephalic size (SL/CS): 0.775–0.830 (m = 0.802). Facial area of the scape absolute setal angle: 0–15°. External area of the scape absolute setal angle: 10–15°; 35–40°. Ground sculpture of submedian area of clypeus: smooth. Median carina of clypeus count: present. Lateral carinae of clypeus count: present. Median anatomical line of propodeal spine angle value to Weber length in lateral view: 47–52°. Spine length vs. absolute cephalic size (SPST/CS): 0.220–0.267 (m = 0.245). Minimum spine distance vs. absolute cephalic size (SPBA/CS): 0.261–0.290 (m = 0.277). Maximum spine distance vs. absolute cephalic size (SPWI/CS): 0.299–0.347 (m = 0.321). Apical spine distance vs. absolute cephalic size (SPTI/CS): 0.291–0.326 (m = 0.321). Maximum mesosoma width vs. absolute cephalic size (MW/CS): 0.606–0.641 (m = 0.624). Metanotal depression count: present. Metanotal depression shape: deep. Dorsal region of mesosoma sculpture: fine areolate ground sculpture, superimposed by dispersed rugae. Lateral region of pronotum sculpture: inconspicuous areolate ground sculpture, main sculpture dispersed costate. Mesopleuron sculpture: fine areolate ground sculpture, superimposed by dispersed rugulae. Metapleuron sculpture: fine areolate ground sculpture, superimposed by dispersed rugulae. Frontal profile of petiolar node contour line in lateral view shape: concave. Dorsal profile of petiolar node contour line angle value to frontal profile of petiole contour line in lateral view: 95–105°. Anterodorsal rim of petiole count: absent medially. Dorsal region of petiole sculpture: ground sculpture areolate, main sculpture dispersed rugose; ground sculpture areolate, main sculpture absent. Dorso-caudal petiolar profile contour line in lateral view shape: straight; concave. Dorsal region of postpetiole sculpture: ground sculpture areolate, main sculpture dispersed rugose; ground sculpture areolate, main sculpture absent.

**Fig 11:**
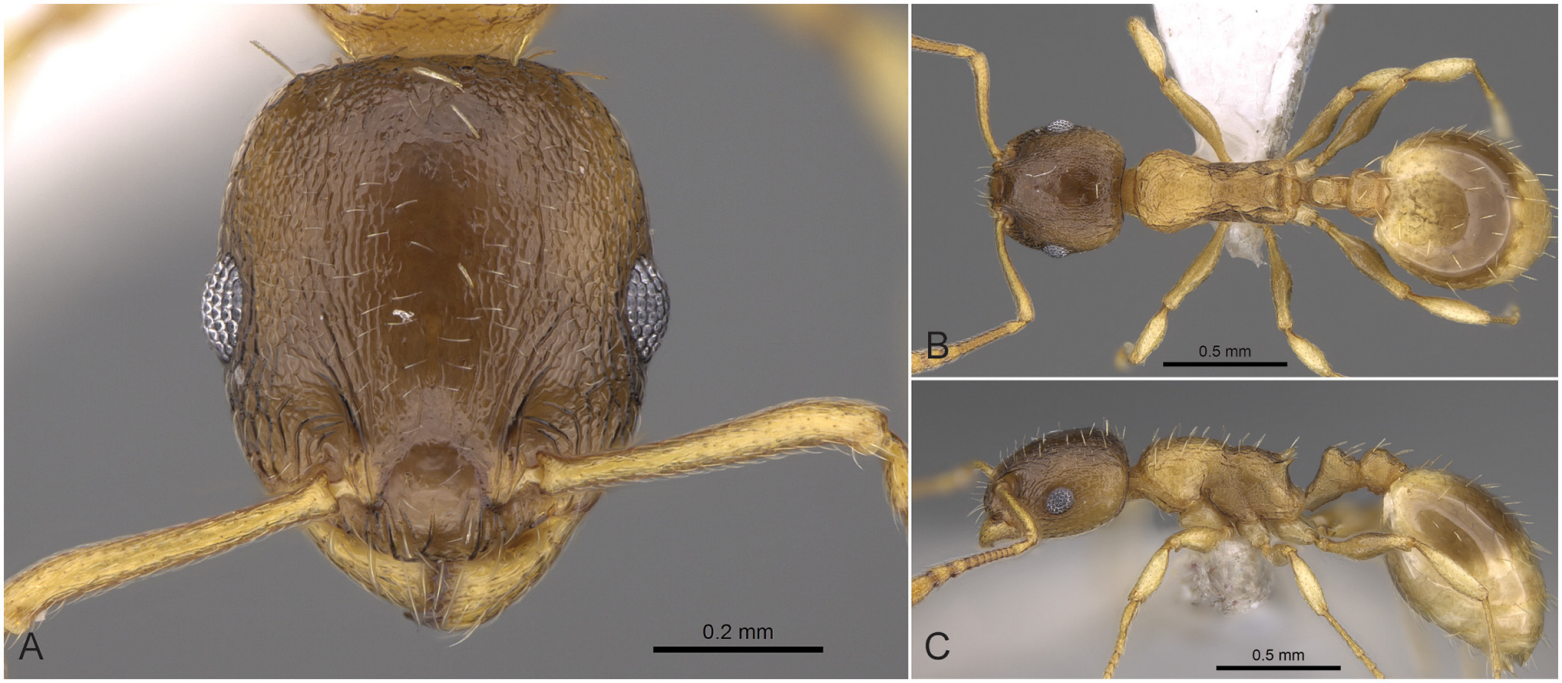
*Temnothorax similis* sp.n. holotype worker. Head of the holotype worker (CASENT0914691) in full face view (A), dorsal view of the body (B), lateral view of the body (C).

#### Differential diagnosis

This species can be distinguished from other species belonging to different species complexes by its inconspicuously sculptured or smooth head and/or its shorter propodeal spines (SPST/CS). *Temnothorax similis* sp. n. differs from the superficially similar *T*. *schoedli* by non-overlapping ranges of PEH/CS and NOH/CS ratios on the level of nest sample means.

Though workers of *T*. *similis* sp. n. differ from those of other species in this complex by having a wider frons (FRS/CS) than *T*. *angustifrons* sp. n., a lower petiole (NOH/CS) than *T*. *lucidus* sp. n., and longer propodeal spines (SPST/CS) than *T*. *subtilis* sp. n. (see S4 Table), nest sample means of these characters slightly overlap between the latter two species and *T*. *similis* sp. n. In order to separate *T*. *similis* sp. n. from *T*. *angustifrons* sp. n., *T*. *lucidus* sp. n., and *T*. *subtilis* sp. n. a discriminant function with reduced character number (D4 = 0.0492*SPST -0.1037*PEH +0.0547*ML -0.0417*CL +2.2655) is provided, that yields 98.6% classification success rate for single individuals.

D4 scores for single individuals:

*T*. *angustifrons* sp.n. (n 67) = -0.732 [-2.383, +1.128], [5–95% percentiles: -2.013, +0.587]

*T*. *lucidus* sp. n. (n 70) = -1.442[-4.654, +0.492], [5–95% percentiles: +-3.701, +0.117]

*T*. *subtilis* sp. n. (n 176) = -1.658 [-5.202, +0.278], [5–95% percentiles: -3.463, -0.105]

*T*. *similis* sp. n. (n 38) = +1.597 [-0.198, +2.966], [5–95% percentiles: +0.240, +2.600]

#### Geographic distribution

This species is known from South and Central Anatolia, Turkey (Fig 8). According to the material available in this study, this species seems to occur in disjunct areas with a 600 km wide gap between the two known finding sites (S1 Table). Due to the fact that morphometric analyses have not revealed convincing morphological separation between the two populations we hold that the described disjunct distribution is more likely a result of sampling error rather than representing areas of two reproductively separated species.

### *Temnothorax subtilis* sp. n.

urn:lsid:zoobank.org:act:20A78840-7999-41A0-A525-CE36346A5E14

#### Etymology

The species epithet „subtilis” (Eng.: fine, thin, slender) refers to the fine, tiny appearance of this species.

#### Type material investigated

**Holotype worker labelled:** TUR:431 Turkey, Taurus Mt., 5 km SW. Akseki, 37,0257 N, 31,7518 E, 950 mH, 02.11.2011, leg. A. Schulz, (HNHM, CASENT0914635), [**TUR:Akseki-5SW-20111102-431**];

**Paratypes:** TUR:431 Turkey, Taurus Mt., 5 km SW. Akseki, 37,0257 N, 31,7518 E, 950 mH, 02.11.2011, leg. A. Schulz, (1# HNHM), [**TUR:Akseki-5SW-20111102-431**]; TUR:430 Turkey, Taurus Mt., 5 km SW. Akseki, 37,0257 N, 31,7518 E, 950mH, 02.11.2011, leg. A. Schulz, (3## HNHM), [**TUR:Akseki-5SW-20111102-430**]; TUR:438 Turkey, Taurus Mt., 5 km SW. Akseki, 37,0257 N, 31,7518 E, 950 mH, 02.11.2011, leg. A. Schulz, (2## HNHM), [**TUR:Akseki-5SW-20111102-438**]; TUR:441 Turkey, Taurus Mt., 5 km SW. Akseki, 37,0257 N, 31,7518 E, 950 mH, 02.11.2011, leg. A. Schulz, (2## HNHM), [**TUR:Akseki-5SW-20111102-441**]; Turkey_08 Antalya, 2 km N. Imrasan Geçidi, 12 km N. Akseki, 37,0924 N, 31,803 E, 1400mH, 03.05.1997. leg. A. Schulz, K. Vock, M. Sanetra, (8## HNHM, 2## CAS CASENT0906012), [**TUR:Imrasan-Geçidi-2N-19970503-117**];

The list of 158 non-type individuals belonging to 50 nest samples of other material investigated is given in S1 Table.

#### Worker (Fig 12A–12C, S1 Table, S4 Table)

Body color: yellow. Body color pattern: mesosoma, antenna and legs, waist and anterior region of 1st gastral tergite lighter than head dorsum and posterior region of gaster. Antenna color pattern: clava concolorous funicle. Absolute cephalic size: 499–628 μm (mean = 556, n = 55). Cephalic length vs. Maximum width of head capsule (CL/CWb): 1.135–1.238 (mean = 1.189). Postocular distance vs. cephalic length (PoOc/CL): 0.374–0.408 (mean = 0.388). Postocular sides of cranium contour frontal view orientation: converging posteriorly. Postocular sides of cranium contour frontal view shape: convex. Vertex contour line in frontal view shape: straight. Vertex sculpture: main sculpture dispersed forked costate, ground sculpture inconspicuous areolate. Genae contour from anterior view orientation: converging. Gena contour line in frontal view shape: feebly convex. Gena sculpture: rugoso-reticulate with feeble areolate ground sculpture. Median region of antennal rim vs. frontal carina in frontal view structure: not fully overlapped by frontal carina. Concentric carinae laterally surrounding antennal foramen count: present. Eye length vs. absolute cephalic size (EL/CS): 0.228–0.268 (mean = 0.249). Frontal carina distance vs. absolute cephalic size (FRS/CS): 0.335–0.375 (mean = 0.360). Longitudinal carinae on median region of frons count: present; absent. Smooth median region on frons count: present. Antennomere count: 12. Scape length vs. absolute cephalic size (SL/CS): 0.735–0.810 (mean = 0.782). Facial area of the scape absolute setal angle: 0–15°. External area of the scape absolute setal angle: 30°. Ground sculpture of submedian area of clypeus: smooth. Median carina of clypeus count: present. Lateral carinae of clypeus count: present. Median anatomical line of propodeal spine angle value to Weber length in lateral view: 47–52°. Spine length vs. absolute cephalic size (SPST/CS): 0.159–0.230 (mean = 0.192). Minimum spine distance vs. absolute cephalic size (SPBA/CS): 0.247–0.300 (mean = 0.272). Maximum spine distance vs. absolute cephalic size (SPWI/CS): 0.266–0.336 (mean = 0.282). Apical spine distance vs. absolute cephalic size (SPTI/CS): 0.256–0.322 (mean = 0.282). Maximum mesosoma width vs. absolute cephalic size (MW/CS): 0.592–0.648 (mean = 0.623). Metanotal depression count: present. Metanotal depression shape: deep. Dorsal region of mesosoma sculpture: fine areolate ground sculpture, superimposed by dispersed rugae. Lateral region of pronotum sculpture: inconspicuous areolate ground sculpture, main sculpture dispersed costate. Mesopleuron sculpture: fine areolate ground sculpture, superimposed by dispersed rugulae. Metapleuron sculpture: fine areolate ground sculpture, superimposed by dispersed rugulae. Frontal profile of petiolar node contour line in lateral view shape: concave. Dorsal profile of petiolar node contour line angle value to frontal profile of petiole contour line in lateral view: 95–105°. Anterodorsal rim of petiole count: absent medially. Dorsal region of petiole sculpture: ground sculpture areolate, main sculpture dispersed rugose; ground sculpture areolate, main sculpture absent. Dorso-caudal petiolar profile contour line in lateral view shape: straight; concave. Dorsal region of postpetiole sculpture: ground sculpture areolate, main sculpture dispersed rugose; ground sculpture areolate, main sculpture absent.

**Fig 12:**
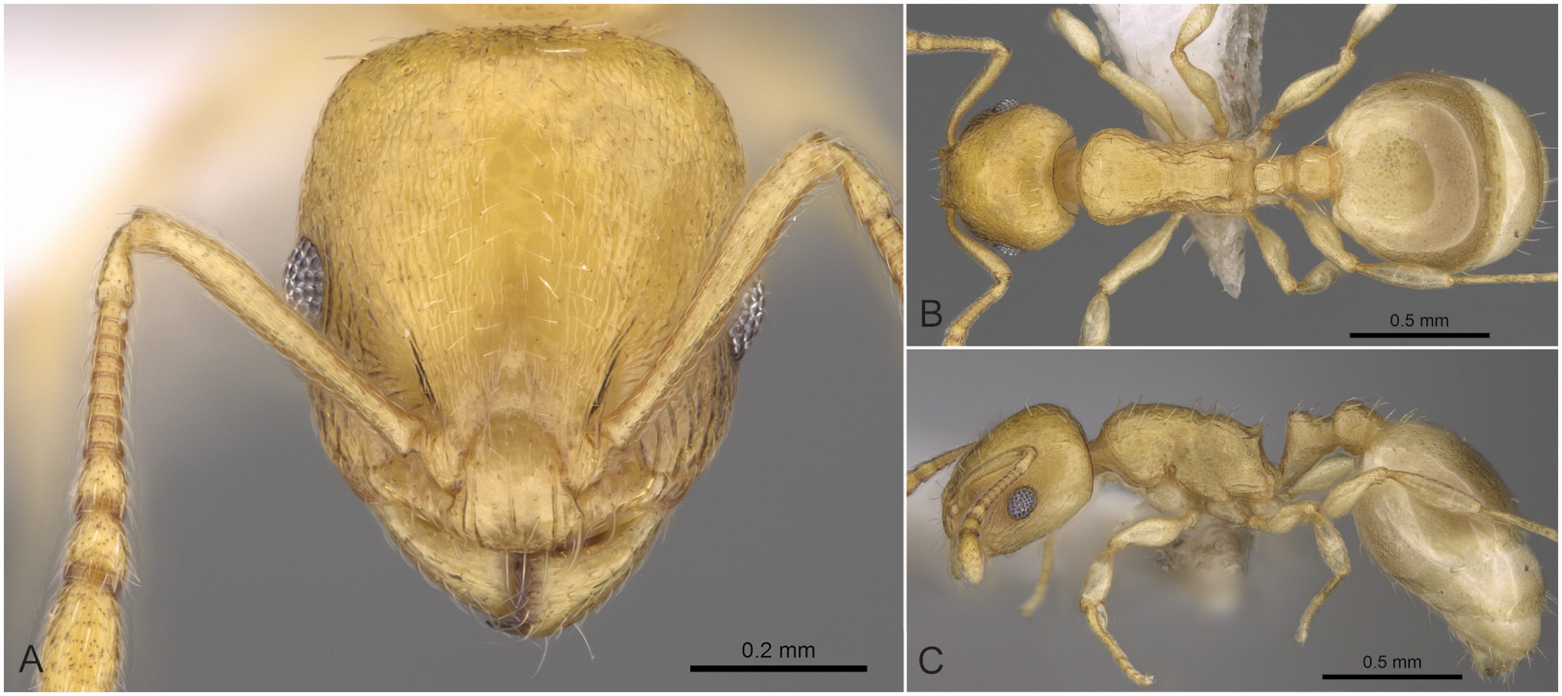
*Temnothorax subtilis* sp.n. holotype worker. Head of the holotype worker (CASENT0914635) in full face view (A), dorsal view of the body (B), lateral view of the body (C).

#### Differential diagnosis

This species has the shortest propodeal spines of all species treated in this revision, and therefore can be separated from other species complexes by the non-overlapping SPST/CS ratio and its smooth and shiny head. Spine length ratio slightly overlaps with that of other species belonging to *T*. *angustifrons* complex yielding 94.5% success in distinguishing nest samples (S5 Table) from *T*. *angustifrons* sp. n., *T*. *lucidus* sp. n., and *T*. *similis* sp. n. Simple FRS/SL ratios help separating this species from *T*. *angustifrons* sp. n. (see differential diagnosis under the latter).

A discriminant function with reduced character number (D4) arrives at 98.6% classification success between single individuals and complete success for nest sample means of *T*. *subtilis* sp. n. and *T*. *similis* sp. n. (see differential diagnosis under *T*. *similis* sp. n.).

Though the SPST/CS ratio (see S4 Table) provides a fairly good quick key to separate nest samples of *T*. *subtilis* sp. n. from those of *T*. *lucidus* sp. n., this character may broadly overlap in single individuals of these two species. In order to determine single workers with high success, a discriminant function with reduced character number (D4 = +0.0717*EL +0.0778*NOH +0.0404*SPST -0.0824*SPBA -10.321) yielding 98.6% classification success rate can be used.

D4 scores for single individuals:

*T*. *subtilis* sp. n. (n 176) = -2.056 [-4.256, +0.259], [5–95% percentiles: -3.620, -0.403]

*T*. *lucidus* sp. n. (n 70) = +2.098[-0.146, +6.298], [5–95% percentiles: +0.576, +3.700]

*Temnothorax subtilis* sp. n. can be easily separated from two additional species of the *T*. *parvulus* complex that occur in Crete, *T*. *ariadnae* sp. n. and *T*. *helenae* sp. n., based on the shiny surface of the head dorsum. In exceptional cases or if dust cover obstructs a clear view of the surface sculpture, several ratios help to separate *T*. *subtilis* sp. n. from *T*. *ariadnae* sp. n.: it has a longer head (CL/CWb), larger eyes (EL/CS) and longer propodeal spines (SPST/CS), and a discriminant function with two characters (D2 = -0.0928*SPST +0.0215*ML -2.811) separates workers of *T*. *subtilis* sp. n. from *T*. *helenae* sp. n. if surface characteristics are not sufficient.

D2 scores for single individuals:

*T*. *helenae* sp. n. (n 169) = -1.760 [-4.946, +2.088], [5–95% percentiles: -3.664, -0.038]

*T*. *subtilis* sp. n. (n 176) = 1.690 [-0.628, +4.455], [5–95% percentiles: +0.044, +3.148]

#### Geographic distribution

This species is known from South Anatolia, Turkey, and Crete (Fig 8).

### Diagnosis of *Temnothorax flavicornis* species-complex

Workers of the *Temnothorax flavicornis* species-complex can be distinguished from those of other complexes treated in this revision by the combination of the following salient features: antennae 11 segmented, yellow to light brown color, head rectangular, significantly longer than broad head (CL/CWb [1.226, 1.299]), sculpture of head dorsum dull: with smooth, or inconspicuously areolate ground sculpture combined with longitudinally rugulose or reticulate main sculpture; long to very long propodeal spines (SPST/CS [0.303, 0.420]), deviating from longitudinal axis of mesosoma by 40–45°; petiolar node in lateral view with a concave frontal profile meeting conspicuously developed truncate dorsum in an obtuse angle (105–115°) with a narrowly rounded transition, without a conspicuous sharp fronto-dorsal ridge on the petiolar node. This peculiar species-complex consists of a single species, *Temnothorax flavicornis* Emery, 1870. Separation of this species is revealed by NC-clustering corroborated by LDA (Fig 2, S5 Table).

### *Temnothorax flavicornis* (Emery, 1870)

*Leptothorax flavicornis* Emery, 1870: 197 (w.q.) ITALY.

Combination in *Temnothorax*: Bolton, 2003: 271.

#### Type material investigated

**Lectotype worker:** „Leptothorax flavicornis Em.”, „Portrei” [‑] Lectotype *Leptothorax flavicornis* Emery, 1870 „Top specimen”, det. A. Schulz & M. Verhaagh 1999 (CASENT0904761) (MSNG);

The list of 44 non-type individuals belonging to 12 nest samples of other material investigated is given in S1 Table.

#### Worker (Fig 13A–13C, S1 Table, S4 Table)

Body color: yellow. Body color pattern: head, mesosoma, antenna and legs excluding femora, waist and anterior region of 1st gastral tergite lighter than femora and posterior region of gaster. Antenna color pattern: clava concolorous funicle. Absolute cephalic size: 465–516 μm (mean = 496, n = 12). Cephalic length vs. Maximum width of head capsule (CL/CWb): 1.226–1.299 (mean = 1.266). Postocular distance vs. cephalic length (PoOc/CL): 0.370–0.394 (mean = 0.384). Postocular sides of cranium contour frontal view orientation: parallel. Postocular sides of cranium contour frontal view shape: straight. Vertex contour line in frontal view shape: straight. Vertex sculpture: main sculpture homogenously forked costate, ground sculpture areolate. Genae contour from anterior view orientation: converging. Gena contour line in frontal view shape: feebly convex. Gena sculpture: rugoso-reticulate with areolate ground sculpture; rugoso-reticulate with feeble areolate ground sculpture. Median region of antennal rim vs. frontal carina in frontal view structure: not fully overlapped by frontal carina. Concentric carinae laterally surrounding antennal foramen count: present. Eye length vs. absolute cephalic size (EL/CS): 0.244–0.271 (mean = 0.262). Frontal carina distance vs. absolute cephalic size (FRS/CS): 0.351–0.373 (mean = 0.364). Longitudinal carinae on median region of frons count: present. Longitudinal carinae on medial region of frons shape: forked. Smooth median region on frons count: absent. Antennomere count: 11. Scape length vs. absolute cephalic size (SL/CS): 0.784–0.817 (mean = 0.803). Facial area of the scape absolute setal angle: 0–15°. External area of the scape absolute setal angle: 30°. Ground sculpture of submedian area of clypeus: smooth. Median carina of clypeus count: present. Lateral carinae of clypeus count: present. Median anatomical line of propodeal spine angle value to Weber length in lateral view: 40–45°. Spine length vs. absolute cephalic size (SPST/CS): 0.303–0.420 (mean = 0.358). Minimum spine distance vs. absolute cephalic size (SPBA/CS): 0.322–0.358 (mean = 0.338). Maximum spine distance vs. absolute cephalic size (SPWI/CS): 0.402–0.506 (mean = 0.458). Apical spine distance vs. absolute cephalic size (SPTI/CS): 0.373–0.482 (mean = 0.432). Maximum mesosoma width vs. absolute cephalic size (MW/CS): 0.622–0.653 (mean = 0.637). Metanotal depression count: present. Metanotal depression shape: shallow. Dorsal region of mesosoma sculpture: rugulose with areolate ground sculpture. Lateral region of pronotum sculpture: areolate ground sculpture, main sculpture forked costate. Mesopleuron sculpture: areolate ground sculpture superimposed by dispersed rugulae. Metapleuron sculpture: areolate ground sculpture superimposed by dispersed rugulae. Frontal profile of petiolar node contour line in lateral view shape: concave. Dorsal profile of petiolar node contour line angle value to frontal profile of petiole contour line in lateral view: 105–115°. Anterodorsal rim of petiole count: absent medially. Dorsal profile of petiolar node contour line in lateral view shape: concave. Dorsal region of petiole sculpture: ground sculpture areolate, main sculpture dispersed rugose. Dorso-caudal petiolar profile contour line in lateral view shape: straight; concave. Dorsal region of postpetiole sculpture: ground sculpture areolate, main sculpture dispersed rugose.

**Fig 13:**
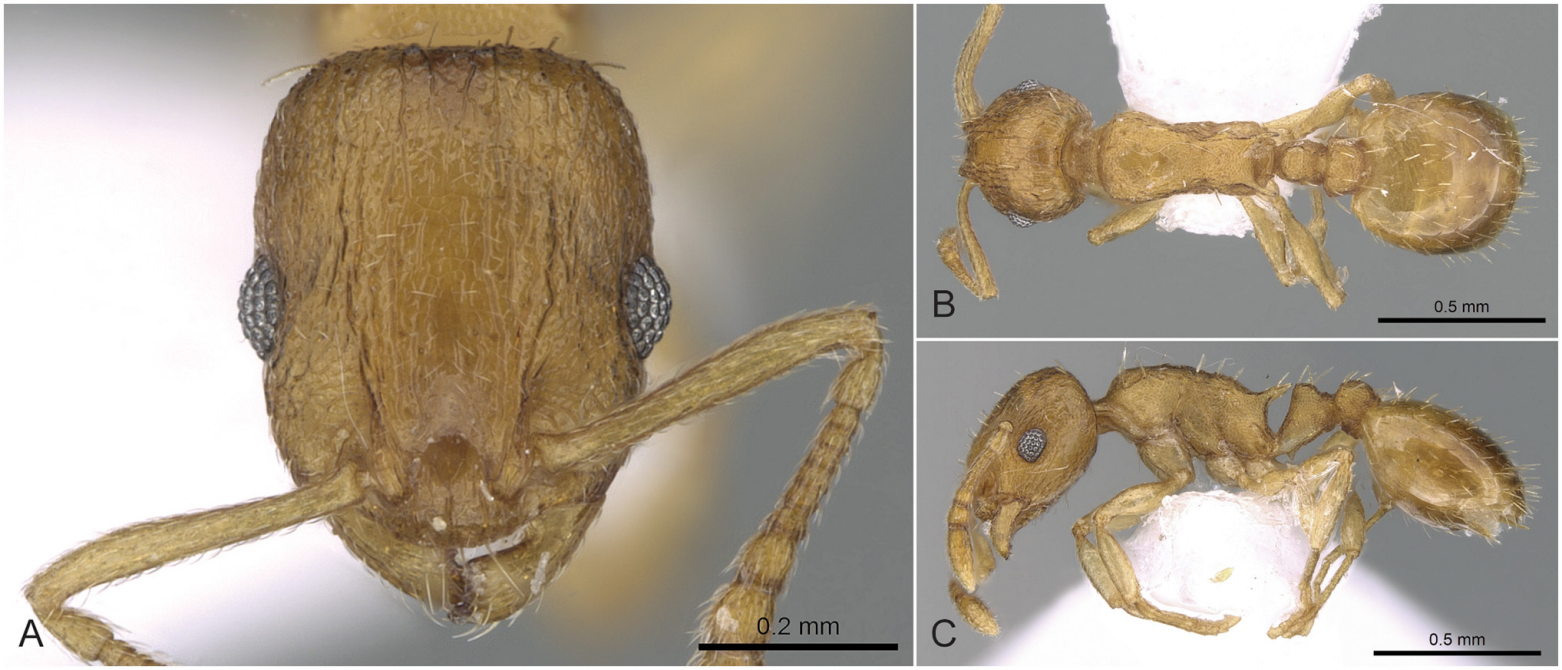
*Temnothorax flavicornis* non-type worker. Head of the holotype worker (CASENT0281556) in full face view (A), dorsal view of the body (B), lateral view of the body (C).

#### Differential diagnosis

Workers of *T*. *flavicornis* might be confused with other long-spined species, i.e., *T*. *laconicus*, *T*. *lichtensteini* and *T*. *parvulus*, but the coarse rugulose or rugulo-reticulate main sculpture on the head dorsum combined with a shiny ground sculpture help to distinguish *T*. *flavicornis* from related species by simple visual inspection. In case specimens are covered by dust, a simple ratio (SPBA/CWb) provides perfect separation of *T*. *flavicornis* and similar species (*T*. *laconicus*, *lichtensteini* and *parvulus*) at the level of nest sample means (see Fig 4). Additional morphometric data of nest sample means for *T*. *flavicornis* and related species (see S4 Table) provide further opportunities for safe separation.

#### Geographic distribution

This species is widely distributed in the Balkans and Italy.

### Diagnosis of *Temnothorax lichtensteini* species-complex

Workers of the *Temnothorax lichtensteini* species-complex can be distinguished from those of other complexes treated in this revision by the combination of the following salient features: dirty yellow to brown color; longer than broad head (CL/CWb [1.181, 1.267]), sculpture of head dorsum dull: with areolate ground sculpture combined with longitudinally rugulose or ruguloso-reticulate (occasionally less conspicuous) main sculpture; long to very long propodeal spines (SPST/CS [0.324, 0.429]), deviating from longitudinal axis of mesosoma by 20–25°; petiolar node in lateral view with a concave frontal profile meeting truncate dorsum in a right angle to an obtuse angle (110–120°) with a narrowly rounded transition, without a conspicuous sharp fronto-dorsal ridge on the petiolar node.

**Fig 14:**
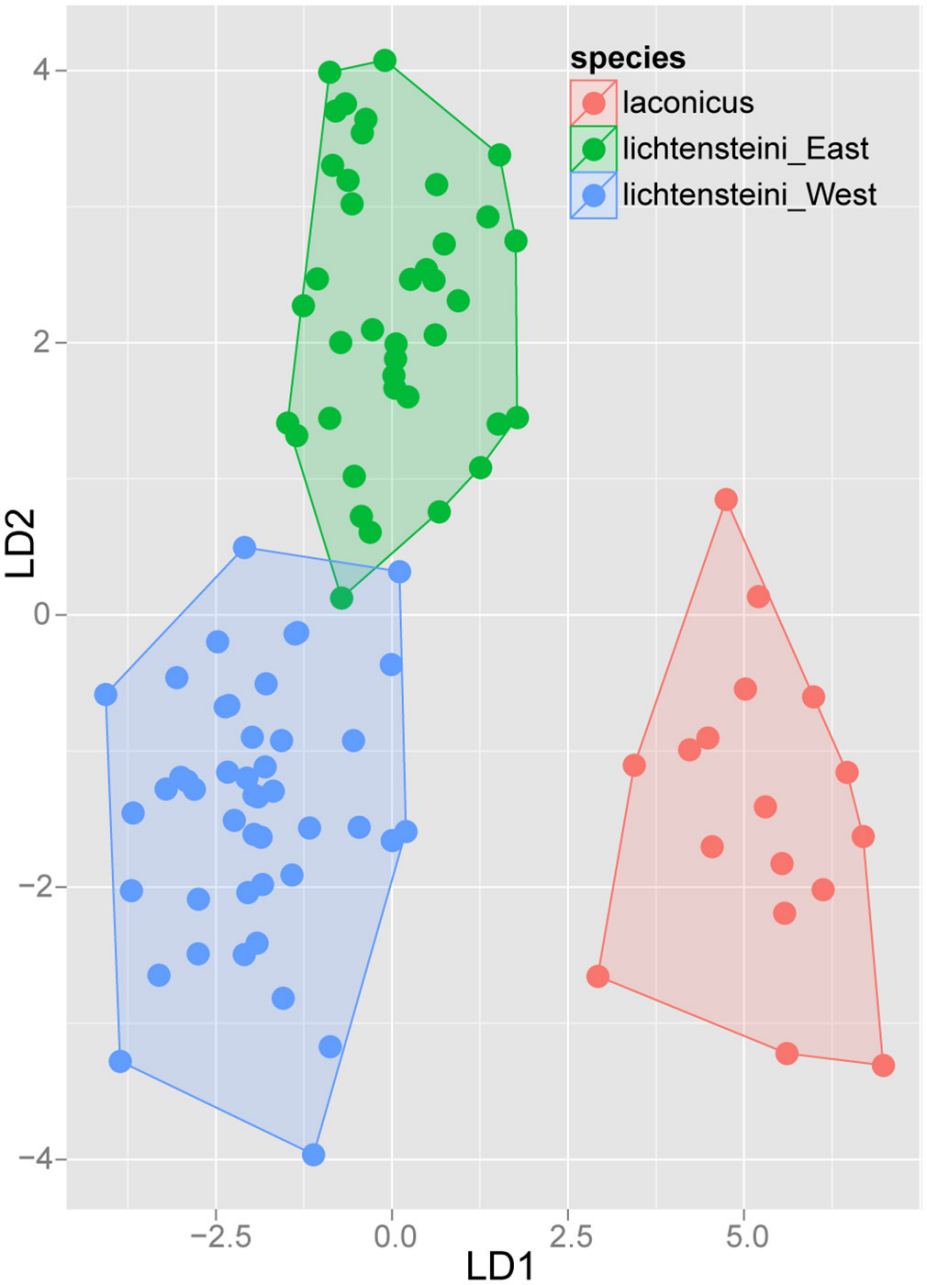
Scatterplot of discriminant scores for *Temnothorax lichtensteini* species-complex is illustrated on the 1^st^ and 2^nd^ axis. Color codes: *T*. *laconicus* (red), *T*. *lichtensteini “Eastern cluster”* (green), *T*. *lichtensteini “Western cluster”* (blue). Convex hulls visualize the range for each group.

**Fig 15:**
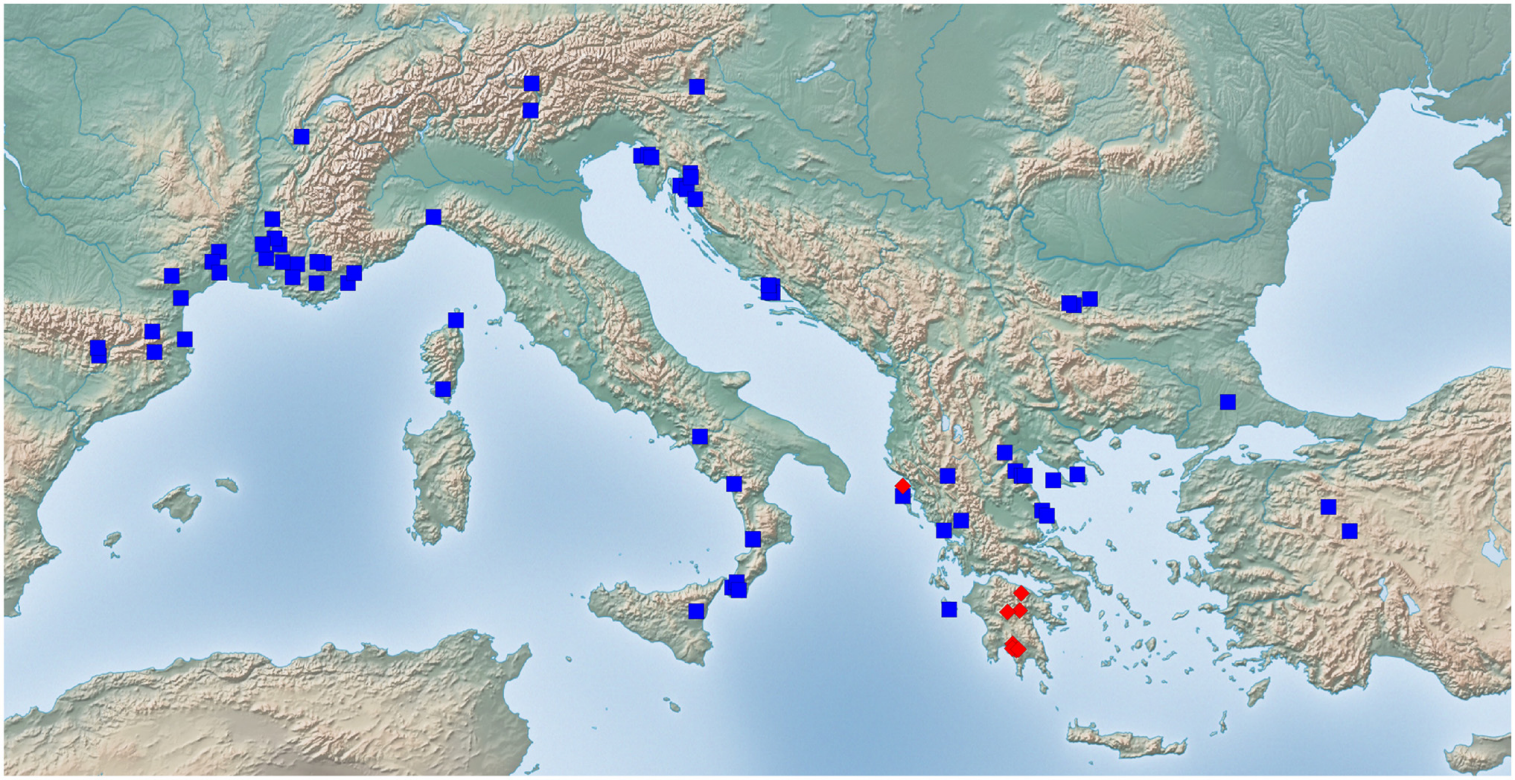
Sampling sites of *Temnothorax lichtensteini* species-complex. Color codes: *T*. *laconicus* (red diamonds), *T*. *lichtensteini* (blue rectangles).

Exploratory NC-clustering corroborated by LDA revealed the existence of two species (*Temnothorax laconicus* Csősz & al., 2014 and *T*. *lichtensteini* Bondroit, 1918) within this complex (S5 Table, Fig 14). Colonies of this species-complex occur in Southern Europe from Spain to Bulgaria and in Turkey (Fig 15).

### *Temnothorax laconicus* Csősz & al., 2014

*Temnothorax laconicus* Csősz & al., 2014: 75 (w.) GREECE.

#### Type material investigated

**Holotype worker:** Taygetos Oros, Street to Profitis Ilias „no. GRE:S_342”, *36*.*968* N, *22*.*404* E, *800* mH, 01.05.2011, leg. A. Schulz (HNHM), [**GRE:Profitis-Ilias-20110501-342**];

**Paratype workers:** Taygetos Oros, Street to Profitis Ilias „no. GRE:S_342”, *36*.*968* N, *22*.*404* E, *800* mH, 01.05.2011, leg. A. Schulz (2## / CAS, CASENT0906682, 3## HNHM), [**GRE:Profitis-Ilias-20110501-342**]; W Taygetos Oros, Pigadia Canyon „no. GRE:S_358”, *36*.*984* N, *22*.*262* E, *700–800* mH, 01.05.2011, leg. A. Schulz (2## HNHM), [**GRE:Pigadia-Canyon-20110501-358**]; W Taygetos Oros, Pigadia Canyon „no. GRE:2011:0356” *36*.*984* N, *22*.*262* E, *700–800* mH, 01.05.2011, leg. A. Schulz (3## HNHM), [**GRE:Pigadia-Canyon-20110501-356**]; Taygetos Oros, Street to Profitis Ilias „no. GRE:2011:0336”, *36*.*968* N, *22*.*404* E, *800* mH, 01.05.2011, leg. A. Schulz (4## HNHM, CASENT0906050), [**GRE:Profitis-Ilias-20110501-336**]; Taygetos Oros, Street to Profitis Ilias „no. GRE:2011:0345” *36*.*968* N, *22*.*404* E, *800* mH, 01.05.2011, leg. A. Schulz (4## HNHM, CASENT0914696), [**GRE:Profitis-Ilias-20110501-345**];

The list of 41 non-type individuals belonging to 10 nest samples of other material investigated is given in S1 Table.

#### Worker (Fig 16A–16C, S1 Table, S4 Table)

Body color: brown. Body color pattern: mesosoma, antenna and legs, waist and anterior region of 1st gastral tergite lighter than head and posterior region of gaster. Antenna color pattern: clava concolorous funicle. Absolute cephalic size: 500–590 μm (mean = 546, n = 16). Cephalic length vs. Maximum width of head capsule (CL/CWb): 1.199–1.258 (mean = 1.228). Postocular distance vs. cephalic length (PoOc/CL): 0.383–0.403 (mean = 0.396). Postocular sides of cranium contour frontal view orientation: converging posteriorly. Postocular sides of cranium contour frontal view shape: strongly convex. Vertex contour line in frontal view shape: straight. Vertex sculpture: main sculpture homogenously forked costate, ground sculpture areolate; main sculpture absent, ground sculpture areolate. Genae contour from anterior view orientation: converging. Gena contour line in frontal view shape: feebly convex. Gena sculpture: rugoso-reticulate with areolate ground sculpture. Median region of antennal rim vs. frontal carina in frontal view structure: not fully overlapped by frontal carina. Concentric carinae laterally surrounding antennal foramen count: present. Eye length vs. absolute cephalic size (EL/CS): 0.229–0.265 (mean = 0.244). Frontal carina distance vs. absolute cephalic size (FRS/CS): 0.329–0.360 (mean = 0.342). Longitudinal carinae on median region of frons count: present. Longitudinal carinae on medial region of frons shape: forked. Smooth median region on frons count: absent. Antennomere count: 12. Scape length vs. absolute cephalic size (SL/CS): 0.766–0.804 (mean = 0.785). Facial area of the scape absolute setal angle: 0–15°. External area of the scape absolute setal angle: 30°; 35–45°. Ground sculpture of submedian area of clypeus: smooth. Median carina of clypeus count: present. Lateral carinae of clypeus count: present. Median anatomical line of propodeal spine angle value to Weber length in lateral view: 20–25°. Spine length vs. absolute cephalic size (SPST/CS): 0.391–0.429 (mean = 0.411). Minimum spine distance vs. absolute cephalic size (SPBA/CS): 0.257–0.311 (mean = 0.283). Maximum spine distance vs. absolute cephalic size (SPWI/CS): 0.401–0.485 (mean = 0.438). Apical spine distance vs. absolute cephalic size (SPTI/CS): 0.381–0.462 (mean = 0.413). Maximum mesosoma width vs. absolute cephalic size (MW/CS): 0.587–0.629 (mean = 0.610). Metanotal depression count: present. Metanotal depression shape: shallow. Dorsal region of mesosoma sculpture: rugulose with areolate ground sculpture. Lateral region of pronotum sculpture: areolate ground sculpture, main sculpture forked costate. Mesopleuron sculpture: areolate ground sculpture superimposed by dispersed rugulae. Metapleuron sculpture: areolate ground sculpture superimposed by dispersed rugulae. Frontal profile of petiolar node contour line in lateral view shape: concave. Dorsal profile of petiolar node contour line angle value to frontal profile of petiole contour line in lateral view: 110–120°. Anterodorsal rim of petiole count: absent medially. Dorsal region of petiole sculpture: ground sculpture areolate, main sculpture dispersed rugose; ground sculpture areolate, main sculpture absent. Dorso-caudal petiolar profile contour line in lateral view shape: straight; concave. Dorsal region of postpetiole sculpture: ground sculpture areolate, main sculpture dispersed rugose; ground sculpture areolate, main sculpture absent.

**Fig 16:**
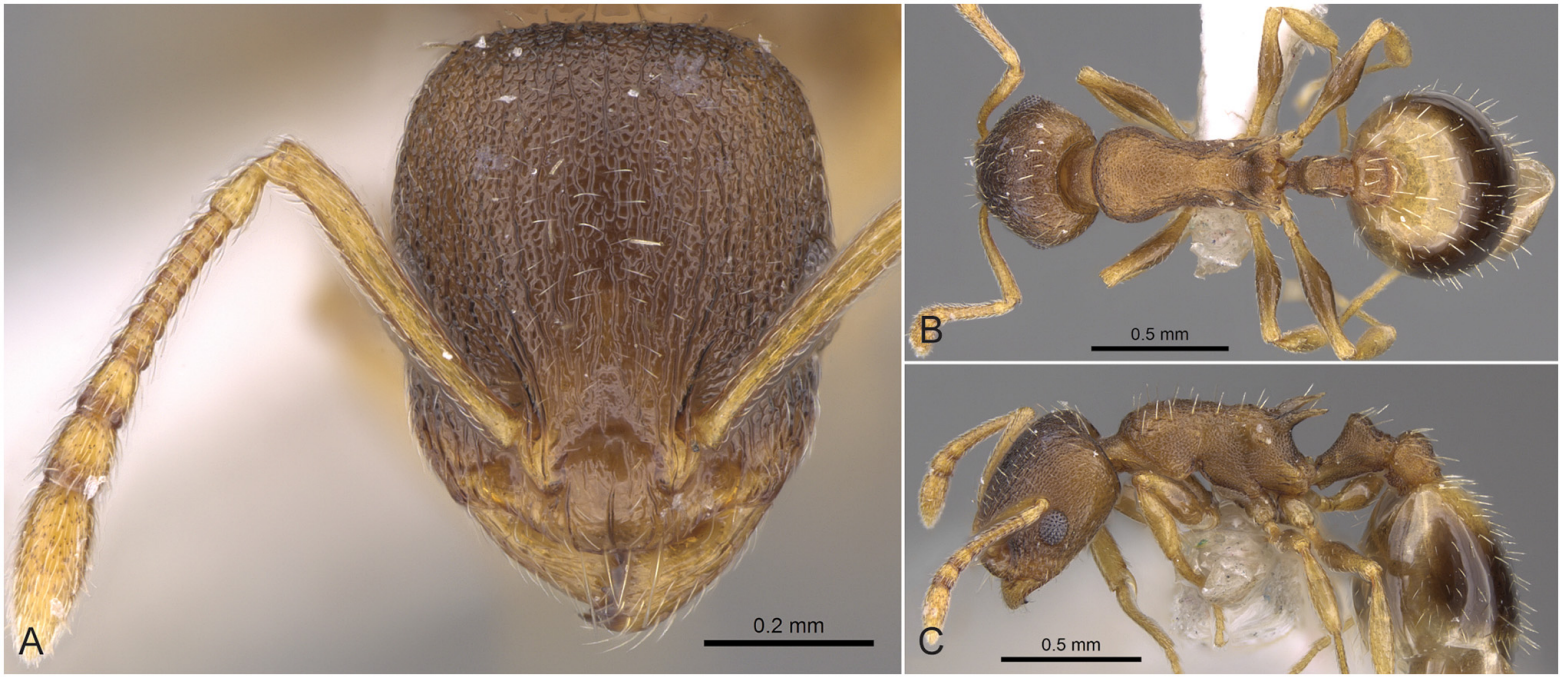
*Temnothorax laconicus* paratype worker. Head of paratype worker (CASENT0914696) in full face view (A), dorsal view of the body (B), lateral view of the body (C).

#### Differential diagnosis

*Temnothorax laconicus* can be distinguished easily from other species by its very long propodeal spines (having the longest propodeal spines of all taxa surveyed within this revision) and its low deviation (20–25°) from the mesosomal axis.

This species is most similar to *T*. *lichtensteini*. The simple ratio SPST/CS does not overlap between the two species at the level of nest sample means (see S4 Table) and is therefore available for separation. For single individuals a simple discriminant (D3 = -0.0498*PoOc -0.0541*FRS +0.0975*SPST +3.3108) function provides a safe determination with very high classification success (98.6%).

D3 scores for single individuals:

*T*. *lichtensteini* (n 295): -0.656 [-4.078, +1.928], [5–95% percentiles:-2.413, +1.121]

*T*. *laconicus* sp.n. (n 64) +3.085 [+0.866, +4.950], [5–95% percentiles:+1.676, +4.471]

#### Geographic distribution

This species is known to occur in the Peloponnese peninsula and Kerkira (Fig 15).

### *Temnothorax lichtensteini* (Bondroit, 1918)

*Leptothorax lichtensteini* Bondroit, 1918: 123 (w.q.m.) FRANCE.

Combination in *Temnothorax*: Bolton, 2003: 271.

#### Type material investigated

**Lectotype and paralectotypes:** 4 workers labeled "Montpellier Jean Lichtenstein", "Leptoth. lichtensteini Type Bondr." and "Lectotype *Leptothorax lichtensteini* Bondroit, 1918 Top specimen det. A.Schulz & M.Verhaagh 1999"; IRSNB Bruxelles; lectotype with CS 546.6. 4 workers labeled "Menton de Dalmas" and "Leptoth. lichtensteini Type Bondr."; IRSNB Bruxelles. (4## IRSNB), [**FRA:lichtensteini-type:montpellier]**;

The list of 298 non-type individuals belonging to 84 nest samples of other material investigated is given in S1 Table.

#### Worker (Fig 17A–17C, S1 Table, S4 Table)

Body color: brown. Body color pattern: mesosoma, antenna and legs, waist and anterior region of 1st gastral tergite lighter than head and posterior region of gaster. Antenna color pattern: clava concolorous funicle. Absolute cephalic size: 474–585 μm (mean = 535, n = 85). Cephalic length vs. Maximum width of head capsule (CL/CWb): 1.181–1.261 (mean = 1.225). Postocular distance vs. cephalic length (PoOc/CL): 0.386–0.418 (mean = 0.401). Postocular sides of cranium contour frontal view orientation: converging posteriorly. Postocular sides of cranium contour frontal view shape: strongly convex. Vertex contour line in frontal view shape: straight. Vertex sculpture: main sculpture homogenously forked costate, ground sculpture areolate; main sculpture absent, ground sculpture areolate. Genae contour from anterior view orientation: converging. Gena contour line in frontal view shape: feebly convex. Gena sculpture: rugoso-reticulate with areolate ground sculpture. Median region of antennal rim vs. frontal carina in frontal view structure: not fully overlapped by frontal carina. Concentric carinae laterally surrounding antennal foramen count: present. Eye length vs. absolute cephalic size (EL/CS): 0.232–0.270 (mean = 0.248). Frontal carina distance vs. absolute cephalic size (FRS/CS): 0.336–0.380 (mean = 0.356). Longitudinal carinae on median region of frons count: present. Longitudinal carinae on medial region of frons shape: forked. Smooth median region on frons count: absent. Antennomere count: 12. Scape length vs. absolute cephalic size (SL/CS): 0.763–0.809 (mean = 0.787). Facial area of the scape absolute setal angle: 0–15°. External area of the scape absolute setal angle: 35–45°; 30°. Ground sculpture of submedian area of clypeus: smooth. Median carina of clypeus count: present. Lateral carinae of clypeus count: present. Median anatomical line of propodeal spine angle value to Weber length in lateral view: 20–25°. Spine length vs. absolute cephalic size (SPST/CS): 0.324–0377 (mean = 0.346). Minimum spine distance vs. absolute cephalic size (SPBA/CS): 0.250–0.302 (mean = 0.272). Maximum spine distance vs. absolute cephalic size (SPWI/CS): 0.335–0.428 (mean = 0.391). Apical spine distance vs. absolute cephalic size (SPTI/CS): 0.318–0.411 (mean = 0.371). Maximum mesosoma width vs. absolute cephalic size (MW/CS): 0.570–0.631 (mean = 0.608). Metanotal depression count: present. Metanotal depression shape: shallow. Dorsal region of mesosoma sculpture: rugulose with areolate ground sculpture. Lateral region of pronotum sculpture: areolate ground sculpture, main sculpture forked costate. Mesopleuron sculpture: areolate ground sculpture superimposed by dispersed rugulae. Metapleuron sculpture: areolate ground sculpture superimposed by dispersed rugulae. Frontal profile of petiolar node contour line in lateral view shape: concave. Dorsal profile of petiolar node contour line angle value to frontal profile of petiole contour line in lateral view: 110–120°. Anterodorsal rim of petiole count: absent medially. Dorsal profile of petiolar node contour line in lateral view shape: concave. Dorsal region of petiole sculpture: ground sculpture areolate, main sculpture dispersed rugose; ground sculpture areolate, main sculpture absent. Dorso-caudal petiolar profile contour line in lateral view shape: straight; concave. Dorsal region of postpetiole sculpture: ground sculpture areolate, main sculpture dispersed rugose; ground sculpture areolate, main sculpture absent.

**Fig 17:**
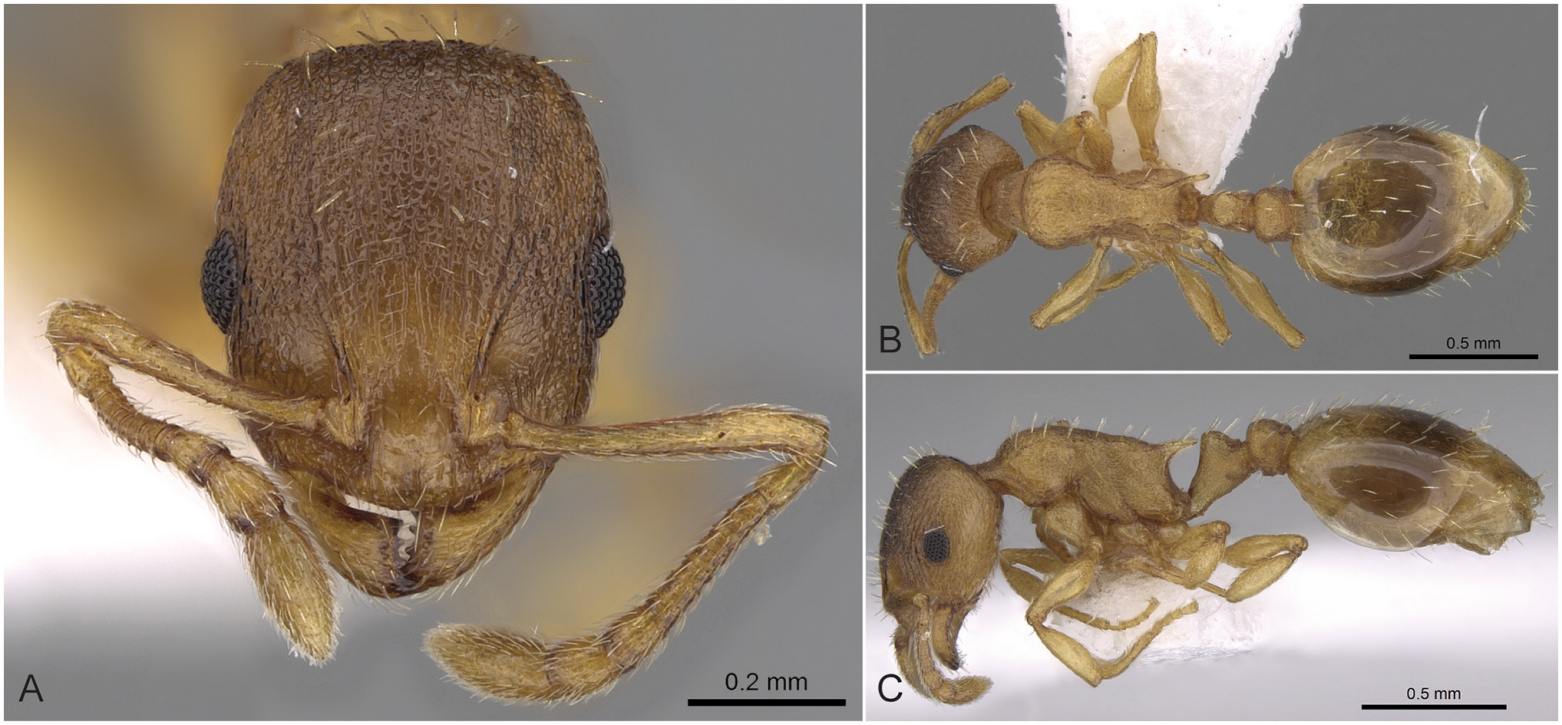
*Temnothorax lichtensteini* non-type worker. Head of the holotype worker (CASENT0906005) in full face view (A), dorsal view of the body (B), lateral view of the body (C).

#### Differential diagnosis

Members of the *Temnothorax lichtensteini* complex (*T*. *lichtensteini* and *T*. *laconicus*) can be easily distinguished from other species treated in this revision by the very long propodeal spines and their low deviation (20–25°) from the mesosomal axis. Other species with long spines have more erect propodeal spines deviating from the mesosomal axis by >35°. How *T*. *lichtensteini* and *T*. *laconicus* can be separated is described under the latter (see above).

#### Geographic distribution

*Temnothorax lichtensteini* is distributed throughout the Northern coast of the Mediterranean basin from Spain to Turkey (Fig 15). Morphological ad molecular analyses suggested the existence of two distinct parapatric metapopulations (Fig 14), “*East Mediterranean cluster*” (Austria, Bulgaria, Croatia, Greece, N-Italy, Turkey) and “*West Mediterranean cluster*” (France, Italy, and Spain) [33], which, however are quite similar in general appearence. Though the”*Eastern*” lineage appears to be more robust than the “*Western*” lineage in some traits (MW/CS, PEW/CS, PPW/CS, SPBA/CS, SPWI/CS, SPTI/CS) [33], these characters broadly overlap and do not provide a safe separation. Due to this similarity and their parapatric occurrence the two lineages have not been raised to species or subspecies rank.

### Diagnosis of *Temnothorax nylanderi* species-complex

Workers of the *Temnothorax nylanderi* species-complex can be distinguished from workers of other complexes treated in this revision by the combination of the following salient features: yellow to light brown color (Peloponnese populations occasionally dark brown to black); slightly longer than broad head (CL/CWb [1.100, 1.196]), sculpture of head dorsum dull: with areolate ground sculpture combined with parallel costulate main sculpture; moderately long to long propodeal spines (SPST/CS [0.253, 0.356]), deviating from longitudinal axis of mesosoma by 32–42°; petiolar node in lateral view with a concave frontal profile meeting truncate dorsum in a right angle to an obtuse angle (88–115°) with a narrowly rounded transition, without a conspicuous sharp fronto-dorsal ridge on the petiolar node.

**Fig 18:**
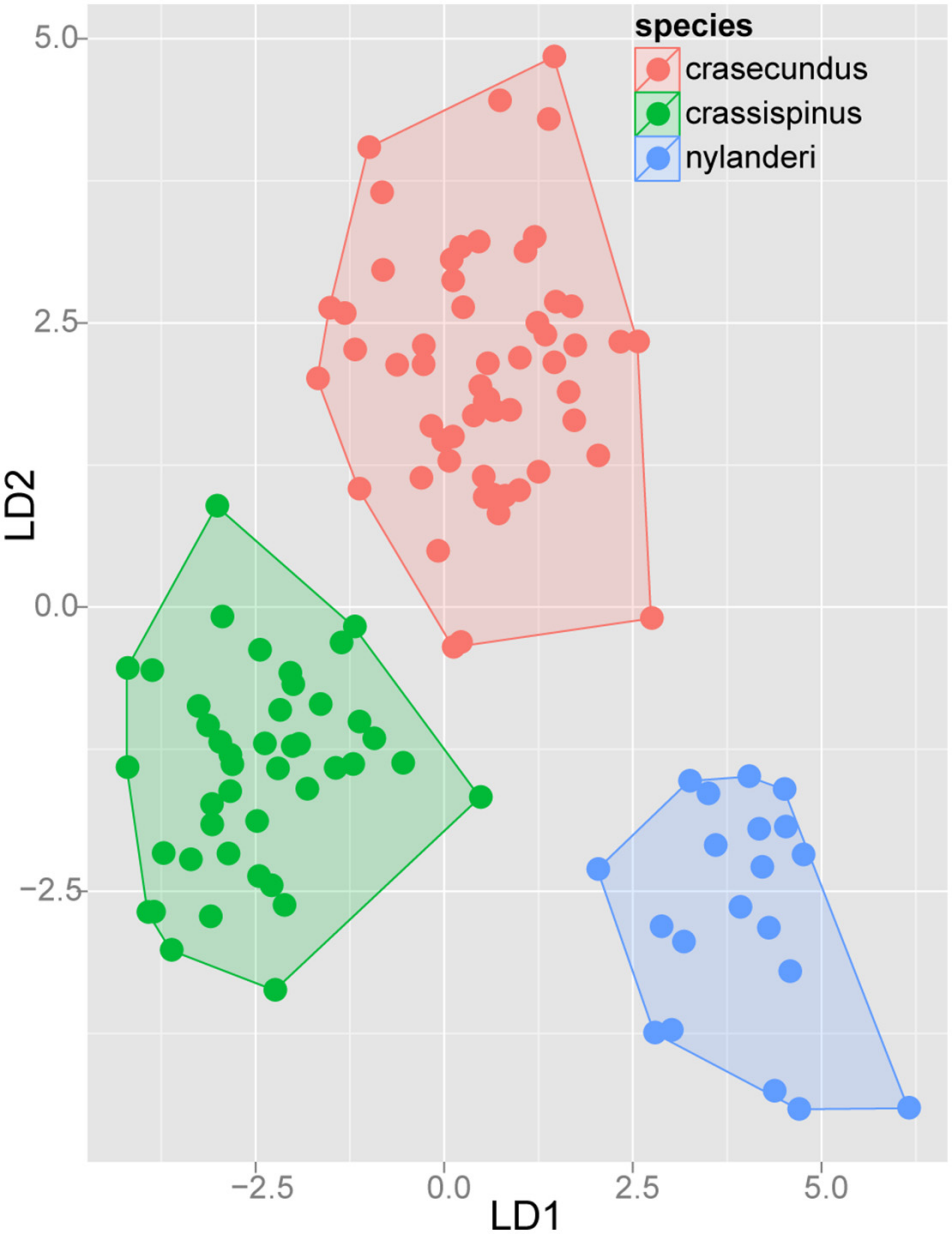
Scatterplot of discriminant scores for *Temnothorax nylanderi* species-complex is illustrated on the 1^st^ and 2^nd^ axis. Color codes: *T*. *crasecundus* (red), *T*. *crassispinus* (green), *T*. *nylanderi* (blue). Convex hulls visualize the range for each group.

**Fig 19:**
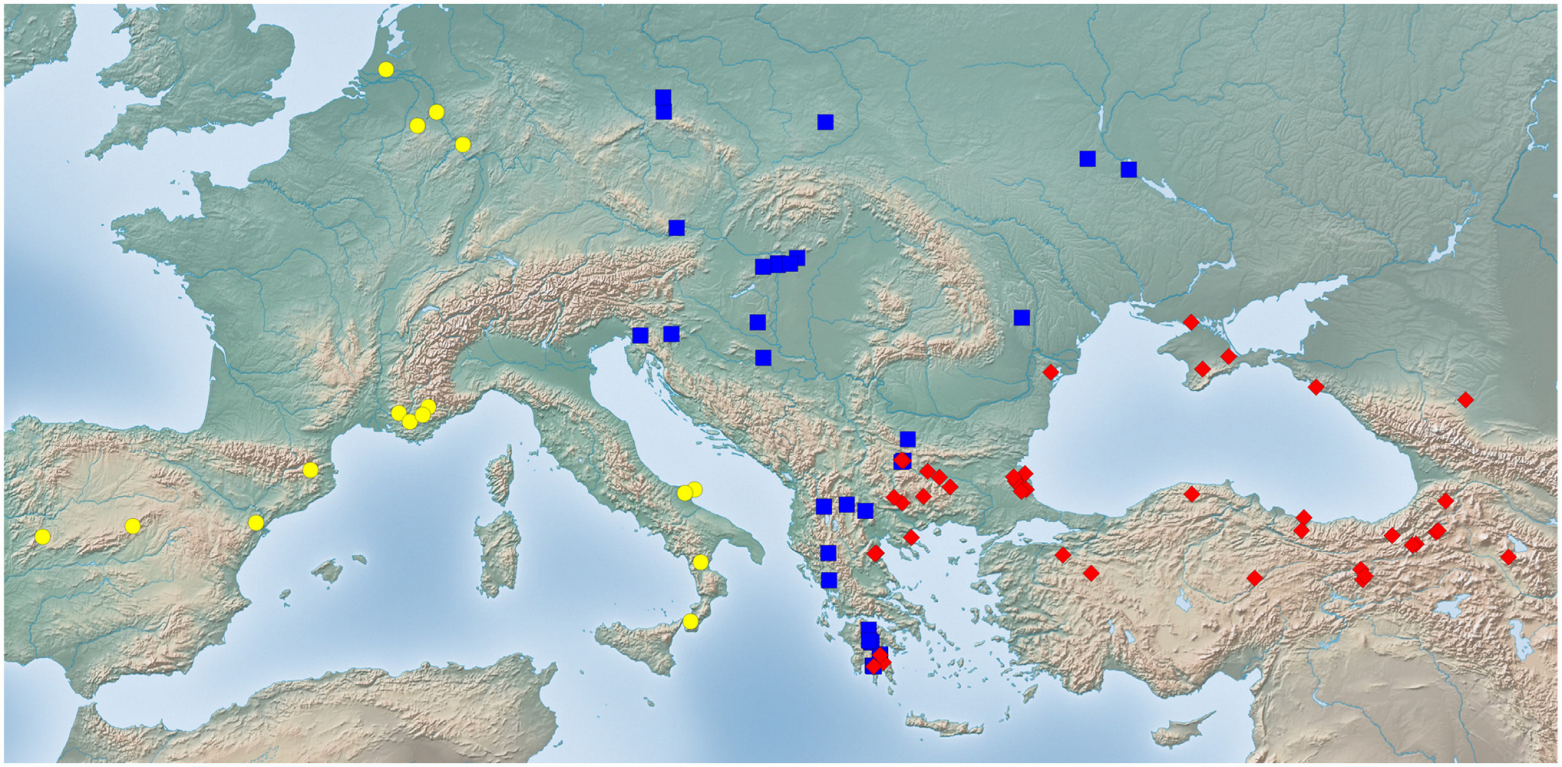
Sampling sites of *Temnothorax nylanderi* species-complex. Color codes: *T*. *crasecundus* (red diamonds), *T*. *crassispinus* (blue rectangles), *T*. *nylanderi* (yellow circles).

According to exploratory NC-clustering and confirmatory analyses (S5 Table, Fig 18) this complex comprises three species: *Temnothorax crasecundus* Seifert & Csősz 2014, *T*. *crassispinus* (Karavaiev, 1926) and *T*. *nylanderi* (Foerster, 1850). This species-complex is widely distributed in Europe and Turkey (Fig 19).

### *Temnothorax crasecundus* Seifert & Csősz 2014

*Temnothorax crasecundus* Seifert & Csősz, 2015: 43 (w.) BULGARIA.

#### Type material investigated

**Paratypes:** BUL:1299 Bulgaria, East Rhodopes, 2 km SE. Novakovo 25 km SE. Asenovgrad 41°53'12"N, 25°5'55"E, 400mH, 12.06.2009, leg. A Schulz, (3 ##, HNHM CASENT0906046), [**BUL:Novakovo-2SE-20090612-1299**];

The list of 167 non-type individuals belonging to 56 nest samples of other material investigated is given in S1 Table.

#### Worker (Fig 20A–20C, S1 Table, S4 Table)

Body color: yellow; brown. Body color pattern: mesosoma, antenna and legs, waist and anterior region of 1st gastral tergite lighter than head dorsum and posterior region of gaster. Antenna color pattern: clava concolorous funicle. Absolute cephalic size: 539–719 μm (mean = 614, n = 57). Cephalic length vs. Maximum width of head capsule (CL/CWb): 1.121–1.196 (mean = 1.155). Postocular distance vs. cephalic length (PoOc/CL): 0.378–0.405 (mean = 0.374). Postocular sides of cranium contour frontal view orientation: converging posteriorly. Postocular sides of cranium contour frontal view shape: strongly convex. Vertex contour line in frontal view shape: straight. Vertex sculpture: main sculpture dispersed forked costate sculpture, ground sculpture areolate; main sculpture parallel costate, ground sculpture areolate. Genae contour from anterior view orientation: converging. Gena contour line in frontal view shape: feebly convex; convex. Gena sculpture: rugoso-reticulate with areolate ground sculpture. Median region of antennal rim vs. frontal carina in frontal view structure: not fully overlapped by frontal carina. Concentric carinae laterally surrounding antennal foramen count: present. Eye length vs. absolute cephalic size (EL/CS): 0.236–0.280 (mean = 0.255). Frontal carina distance vs. absolute cephalic size (FRS/CS): 0.359–0.397 (mean = 0.374). Longitudinal carinae on median region of frons count: present. Longitudinal carinae on medial region of frons shape: not forked. Smooth median region on frons count: absent. Antennomere count: 12. Scape length vs. absolute cephalic size (SL/CS): 0.763–0.836 (mean = 0.791). Facial area of the scape absolute setal angle: 0–15°. External area of the scape absolute setal angle: 30°. Ground sculpture of submedian area of clypeus: smooth. Median carina of clypeus count: present. Lateral carinae of clypeus count: present. Median anatomical line of propodeal spine angle value to Weber length in lateral view: 32–35°. Spine length vs. absolute cephalic size (SPST/CS): 0.253–0.322 (mean = 0.289). Minimum spine distance vs. absolute cephalic size (SPBA/CS): 0.279–0.321 (mean = 0.298). Maximum spine distance vs. absolute cephalic size (SPWI/CS): 0.311–0.392 (mean = 0.350). Apical spine distance vs. absolute cephalic size (SPTI/CS): 0.300–0.369 (mean = 0.331). Maximum mesosoma width vs. absolute cephalic size (MW/CS): 0.612–0.662 (MEAN = 0.631). Metanotal depression count: present. Metanotal depression shape: shallow. Dorsal region of mesosoma sculpture: rugulose with areolate ground sculpture. Lateral region of pronotum sculpture: areolate ground sculpture, main sculpture forked costate. Mesopleuron sculpture: areolate ground sculpture superimposed by dispersed rugulae. Metapleuron sculpture: areolate ground sculpture superimposed by dispersed rugulae. Frontal profile of petiolar node contour line in lateral view shape: concave. Dorsal profile of petiolar node contour line angle value to frontal profile of petiole contour line in lateral view: 88–104°. Anterodorsal rim of petiole count: absent medially. Dorsal profile of petiolar node contour line in lateral view shape: slightly convex. Dorsal region of petiole sculpture: ground sculpture areolate, main sculpture dispersed rugose; ground sculpture areolate, main sculpture absent. Dorso-caudal petiolar profile contour line in lateral view shape: straight; concave. Dorsal region of postpetiole sculpture: ground sculpture areolate, main sculpture dispersed rugose; ground sculpture areolate, main sculpture absent.

**Fig 20:**
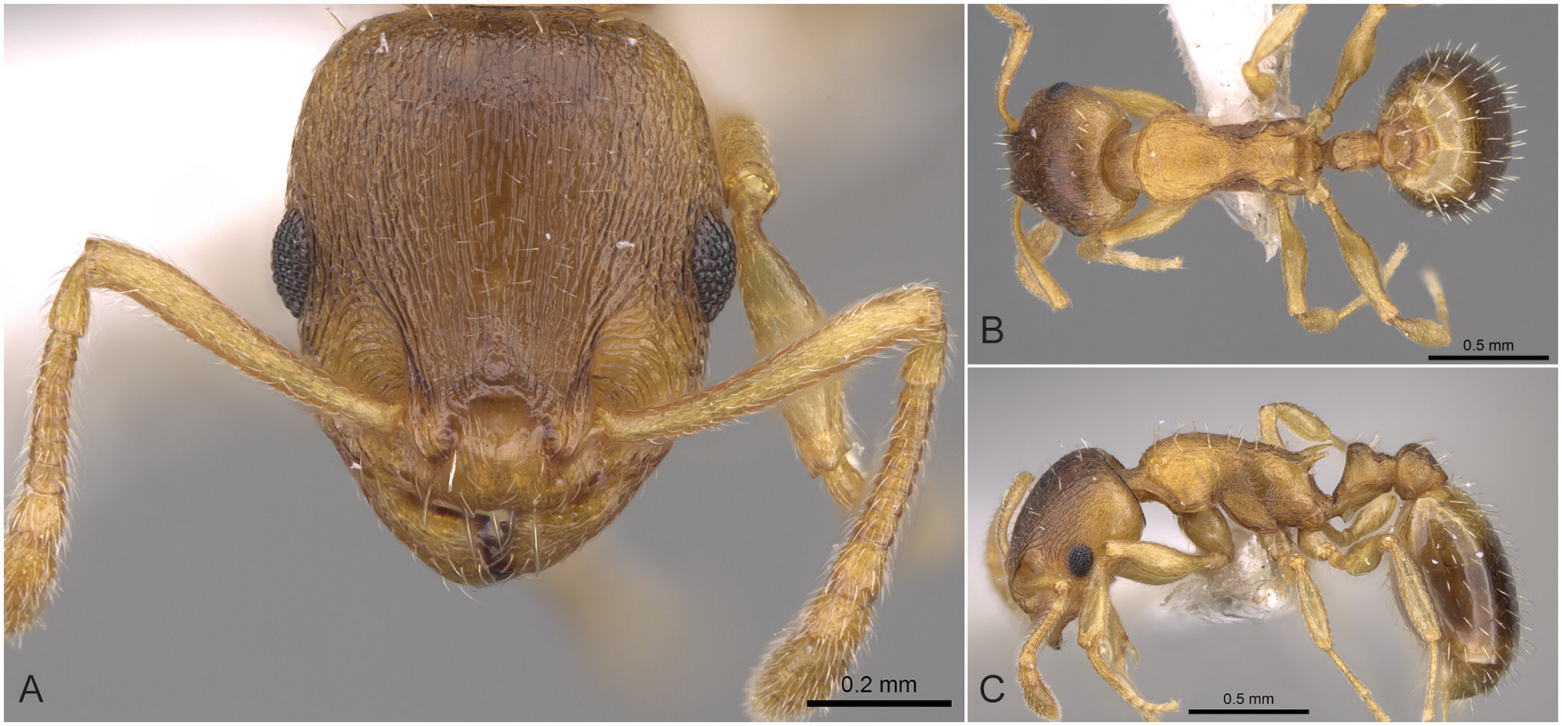
*Temnothorax crasecundus* non-type worker. Head of the holotype worker (CASENT0914699) in full face view (A), dorsal view of the body (B), lateral view of the body (C).

#### Differential diagnosis

This species is easily confused with its parapatric relative, *T*. *crassispinus*. The D4 function that separates these two species is given under the latter.

*Temnothorax crasecundus* shares most of its surface sculpturing and general shape characteristics with *T*. *helenae* sp. n., despite the fact that they belong to different species complexes based on molecular phylogeny and morphometrics. In addition, the distribution of these species broadly overlaps in Bulgaria, Greece, and Turkey. A simple ratio (PoOC/NOH) yields a rather reliable discrimination with minor overlap between them (Fig 4). Other characters may also help in correct determination: *T*. *crasecundus* is larger (CS), has a wider frons (FRS/CS), higher petiolar node (NOH/CS), and longer propodeal spines (SPST/SC) than *T*. *helenae* sp. n. (see S4 Table).

#### Geographic distribution

This species is known to occur in the Balkans, Eastern Europe, and Turkey (Fig 19). Specimens collected as host of the slave-making ant *Myrmoxenus tamarae* in Daba, Georgia also appear to belong to this species [55].

### *Temnothorax crassispinus* (Karavaiev, 1926)

*Leptothorax nylanderi* var. *crassispina* Karavaiev, 1926: 69 (w.q.) UKRAINE.

Combination in *Temnothorax*: Bolton, 2003: 271.

Senior synonym of *Temnothorax slavonicus*: Radchenko, 2000: 44.

#### Type material investigated

**Syntype workers of *Leptothorax nylanderi* var. *crassispina* Karavajev, 1926:** Golossev near Kiev, Leg. Karawajew, "No. 3057 Col. Karawajew" (7## SIZK, CASENT0914703) [**UKR:Golossev-crassispinus-TYPE**];

**Paratype workers of *Leptothorax nylanderi slavonicus* Seifert, 1995:** Germany, Kr. Görlitz, Hutberg Schönau-Berzdorf, 19.03.1993. Seifert (4## SMNG) **[GER:Hutberg-slavonicus-TYPE];**

The list of 119 non-type individuals belonging to 40 nest samples of other material investigated is given in S1 Table.

#### Worker (Fig 21A–21C, S1 Table, S4 Table)

Body color: brown; yellow. Body color pattern: mesosoma, antenna and legs, waist and anterior region of 1st gastral tergite lighter than head and posterior region of gaster. Antenna color pattern: clava concolorous funicle. Absolute cephalic size: 544–688 μm (mean = 623, n = 42). Cephalic length vs. Maximum width of head capsule (CL/CWb): 1.100–1.180 (mean = 1.140). Postocular distance vs. cephalic length (PoOc/CL): 0.379–0.403 (mean = 0.390). Postocular sides of cranium contour frontal view orientation: converging posteriorly. Postocular sides of cranium contour frontal view shape: strongly convex. Vertex contour line in frontal view shape: straight. Vertex sculpture: main sculpture parallel costate, ground sculpture areolate. Genae contour from anterior view orientation: converging. Gena contour line in frontal view shape: convex. Gena sculpture: rugoso-reticulate with areolate ground sculpture. Median region of antennal rim vs. frontal carina in frontal view structure: not fully overlapped by frontal carina. Concentric carinae laterally surrounding antennal foramen count: present. Eye length vs. absolute cephalic size (EL/CS): 0.247–0.268 (mean = 0.256). Frontal carina distance vs. absolute cephalic size (FRS/CS): 0.362–0.399 (mean = 0.377). Longitudinal carinae on median region of frons count: present. Longitudinal carinae on medial region of frons shape: not forked. Smooth median region on frons count: absent. Antennomere count: 12. Scape length vs. absolute cephalic size (SL/CS): 0.756–0.811 (mean = 0.784). Facial area of the scape absolute setal angle: 0–15°. External area of the scape absolute setal angle: 30°. Ground sculpture of submedian area of clypeus: smooth. Median carina of clypeus count: present. Lateral carinae of clypeus count: present. Median anatomical line of propodeal spine angle value to Weber length in lateral view: 32–42°. Spine length vs. absolute cephalic size (SPST/CS): 0.288–0.356 (mean = 0.329). Minimum spine distance vs. absolute cephalic size (SPBA/CS): 0.282–0.339 (mean = 0.312). Maximum spine distance vs. absolute cephalic size (SPWI/CS): 0.362–0.421 (mean = 0.389). Apical spine distance vs. absolute cephalic size (SPTI/CS): 0.342–0.397 (mean = 0.366). Maximum mesosoma width vs. absolute cephalic size (MW/CS): 0.509–0.662 (mean = 0.626). Metanotal depression count: present. Metanotal depression shape: shallow. Dorsal region of mesosoma sculpture: rugulose with areolate ground sculpture. Lateral region of pronotum sculpture: areolate ground sculpture, main sculpture forked costate. Mesopleuron sculpture: areolate ground sculpture superimposed by dispersed rugulae. Metapleuron sculpture: areolate ground sculpture superimposed by dispersed rugulae. Frontal profile of petiolar node contour line in lateral view shape: concave. Dorsal profile of petiolar node contour line angle value to frontal profile of petiole contour line in lateral view: 100–115°. Anterodorsal rim of petiole count: absent medially. Dorsal profile of petiolar node contour line in lateral view shape: slightly convex. Dorsal region of petiole sculpture: ground sculpture areolate, main sculpture dispersed rugose; ground sculpture areolate, main sculpture absent. Dorso-caudal petiolar profile contour line in lateral view shape: straight; concave. Dorsal region of postpetiole sculpture: ground sculpture areolate, main sculpture dispersed rugose; ground sculpture areolate, main sculpture absent.

**Fig 21:**
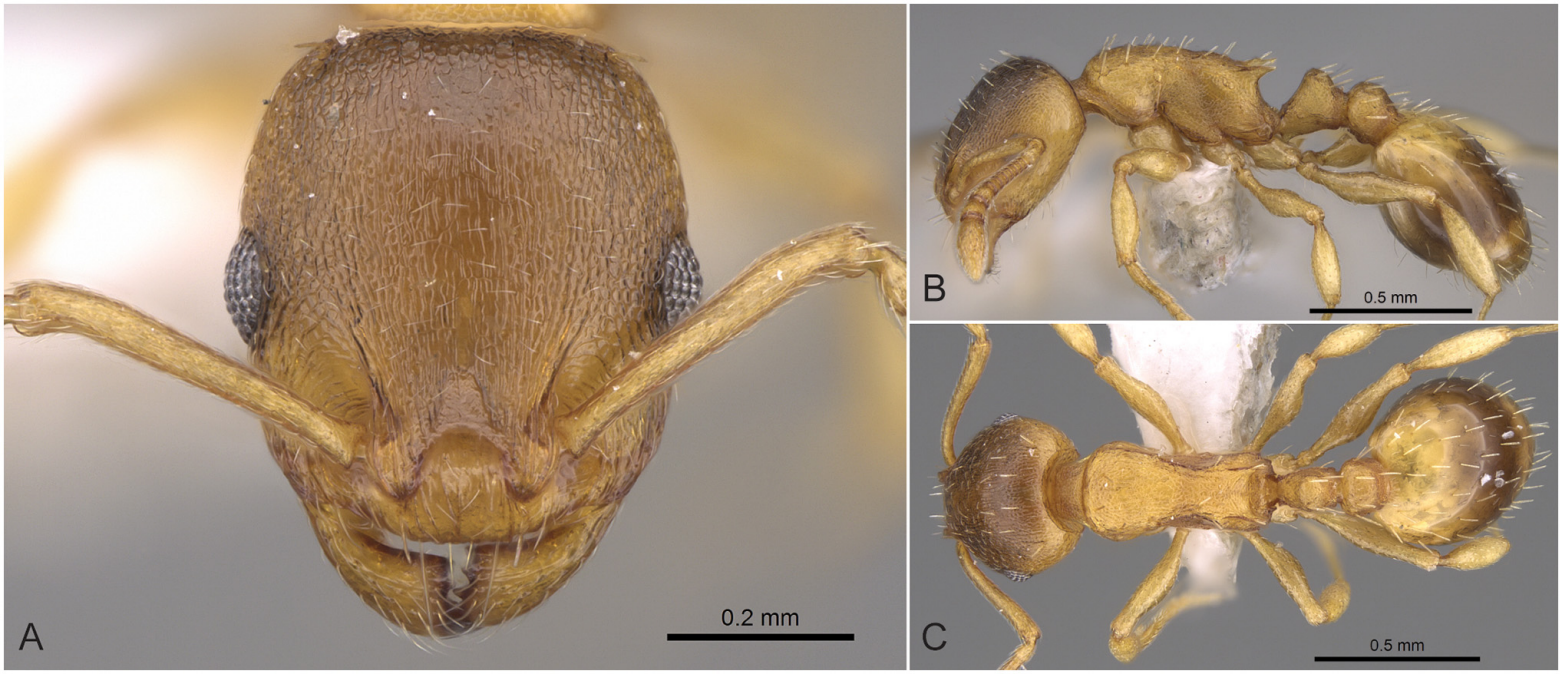
*Temnothorax crassispinus* non-type worker. Head of the holotype worker (CASENT0914700) in full face view (A), dorsal view of the body (B), lateral view of the body (C).

#### Differential diagnosis

*Temnothorax crassispinus* may be confused with other long-spined species treated in this revision: *T*. *angulinodis* sp.n., *T*. *laconicus*, *T*. *lichtensteini* and *T*. *parvulus*. *Temnothorax angulinodis* sp.n. clearly differs from *T*. *crassispinus* by its sharply angulate petiolar node in lateral view (72–82°). In *T*. *crassispinus*, the frontal profile and the truncate dorsum of the petiole meet in an obtuse angle (100–115°). The deviation of the propodeal spines from longitudinal mesosomal axis (in lateral view) helps to separate *T*. *crassispinus* (32–42°) from *T*. *laconicus* and *T*. *lichtensteini* (20–25°). *Temnothorax parvulus* differs from *T*. *crassispinus* in the surface sculpturing on the head dorsum. If the samples are dust-covered, other measures can also help: *T*. *crassispinus* is considerably larger (CS), has a higher petiolar node (SPST/CS), and a wider head (CL/CWb) than *T*. *parvulus* (S4 Table).

*Temnothorax crassispinus* shares most of its main characteristics, shape and surface sculpturing, with its siblings, *T*. *crasecundus* and *T*. *nylanderi*. The simple propodeal spine length ratio (SPST/CS) helps to separate nest samples of *T*. *crassispinus* from those of *T*. *nylanderi* without error, but the same character overlaps between nest sample means of *T*. *crassispinus* and *T*. *crasecundus*. The shortest discriminant formula (D4) that separates *T*. *crassispinus* from *T*. *crasecundus* with a classification success rate 95% in single individuals and 97% in nest sample means is D4 = +0.0392*SL -0.0746*SPST +0.0933*SPL -0.0295*SPWI -7.2179.

D4 scores for single individuals:

*T*. *crassispinus* (n 139) = -1.458 [-4.225, +0.521], [5–95% percentiles: -3.064, -0.075]

*T*. *crasecundus* (n 161) = +1.458 [-2,386, +3.947], [5–95% percentiles:-0.055, +3.387]

D4 scores for nest sample means:

*T*. *crassispinus* (n 45) = -1.440 [-2.789, -0.166], [5–95% percentiles:-2.511, -0.353]

*T*. *crasecundus* (n 54) = +1.479 [-0.381, +3.360], [5–95% percentiles:-0.035, +2.849]

#### Geographic distribution

This species is distributed from the Balkans to Central Europe (Fig 19). The distributional area of *Temnothorax crassispinus* lays between the ranges of its two parapatric relatives, *T*. *nylanderi* in the West and *T*. *crasecundus* in the East.

### *Temnothorax nylanderi* (Foerster, 1850)

*Myrmica nylanderi* Foerster, 1850: 53 (m.) GERMANY.

Combination in *Temnothorax*: Bolton, 2003: 271.

#### Type material investigated

**Type material of *Leptothorax nylanderi* Foerster, 1852** is not available and most probably lost. Altogether 6 workers belonging to two nest series from the type locality „Aachen” were investigated: Germany, vic. Aachen-Brand, 5km SE Aachen, 50.7506 N, 6.1202 E, 200 mH, 21.06.1999. leg. A. Schulz, (3 ##, HNHM), [**GER:Aachen-5SE-19990621-1**]; and (3 ##, HNHM), [**GER:Aachen-5SE-19990621-2**] with the same label data;

The list of 60 non-type individuals belonging to 20 nest samples of other material investigated is given in S1 Table.

#### Worker (Fig 22A–22C, S1 Table, S4 Table)

Body color: brown; yellow. Body color pattern: mesosoma, antenna and legs, waist and anterior region of 1st gastral tergite lighter than head dorsum and posterior region of gaster. Antenna color pattern: clava concolorous funicle. Absolute cephalic size: 587–678 μm (mean = 625, n = 20). Cephalic length vs. Maximum width of head capsule (CL/CWb): 1.121–1.160 (mean = 1.140). Postocular distance vs. cephalic length (PoOc/CL): 0.379–0.402 (mean = 0.391). Postocular sides of cranium contour frontal view orientation: converging posteriorly. Postocular sides of cranium contour frontal view shape: strongly convex. Vertex contour line in frontal view shape: straight. Vertex sculpture: main sculpture parallel costate, ground sculpture areolate; main sculpture absent, ground sculpture areolate. Genae contour from anterior view orientation: converging. Gena contour line in frontal view shape: convex. Gena sculpture: rugoso-reticulate with areolate ground sculpture. Median region of antennal rim vs. frontal carina in frontal view structure: not fully overlapped by frontal carina. Concentric carinae laterally surrounding antennal foramen count: present. Eye length vs. absolute cephalic size (EL/CS): 0.245–0.268 (mean = 0.254). Frontal carina distance vs. absolute cephalic size (FRS/CS): 0.354–0.383 (mean = 0.373). Longitudinal carinae on median region of frons count: present. Longitudinal carinae on medial region of frons shape: not forked. Smooth median region on frons count: absent. Antennomere count: 12. Scape length vs. absolute cephalic size (SL/CS): 0.757–0.798 (mean = 0.777). Facial area of the scape absolute setal angle: 0–15°. External area of the scape absolute setal angle: 30°. Ground sculpture of submedian area of clypeus: smooth. Median carina of clypeus count: present. Lateral carinae of clypeus count: present. Median anatomical line of propodeal spine angle value to Weber length in lateral view: 35–42°. Spine length vs. absolute cephalic size (SPST/CS): 0.265–0.297 (mean = 0.280). Minimum spine distance vs. absolute cephalic size (SPBA/CS): 0.263–0.294 (mean = 0.280). Maximum spine distance vs. absolute cephalic size (SPWI/CS): 0.321–0.362 (mean = 0.343). Apical spine distance vs. absolute cephalic size (SPTI/CS): 0.298–0.339 (mean = 0.320). Maximum mesosoma width vs. absolute cephalic size (MW/CS): 0.610–0.646 (mean = 0.624). Metanotal depression count: present. Metanotal depression shape: shallow. Dorsal region of mesosoma sculpture: rugulose with areolate ground sculpture. Lateral region of pronotum sculpture: areolate ground sculpture, main sculpture forked costate. Mesopleuron sculpture: areolate ground sculpture superimposed by dispersed rugulae. Metapleuron sculpture: areolate ground sculpture superimposed by dispersed rugulae. Frontal profile of petiolar node contour line in lateral view shape: concave. Dorsal profile of petiolar node contour line angle value to frontal profile of petiole contour line in lateral view: 100–115°. Anterodorsal rim of petiole count: absent medially. Dorsal profile of petiolar node contour line in lateral view shape: slightly convex. Dorsal region of petiole sculpture: ground sculpture areolate, main sculpture dispersed rugose; ground sculpture areolate, main sculpture absent. Dorso-caudal petiolar profile contour line in lateral view shape: straight; concave. Dorsal region of postpetiole sculpture: ground sculpture areolate, main sculpture dispersed rugose; ground sculpture areolate, main sculpture absent.

**Fig 22:**
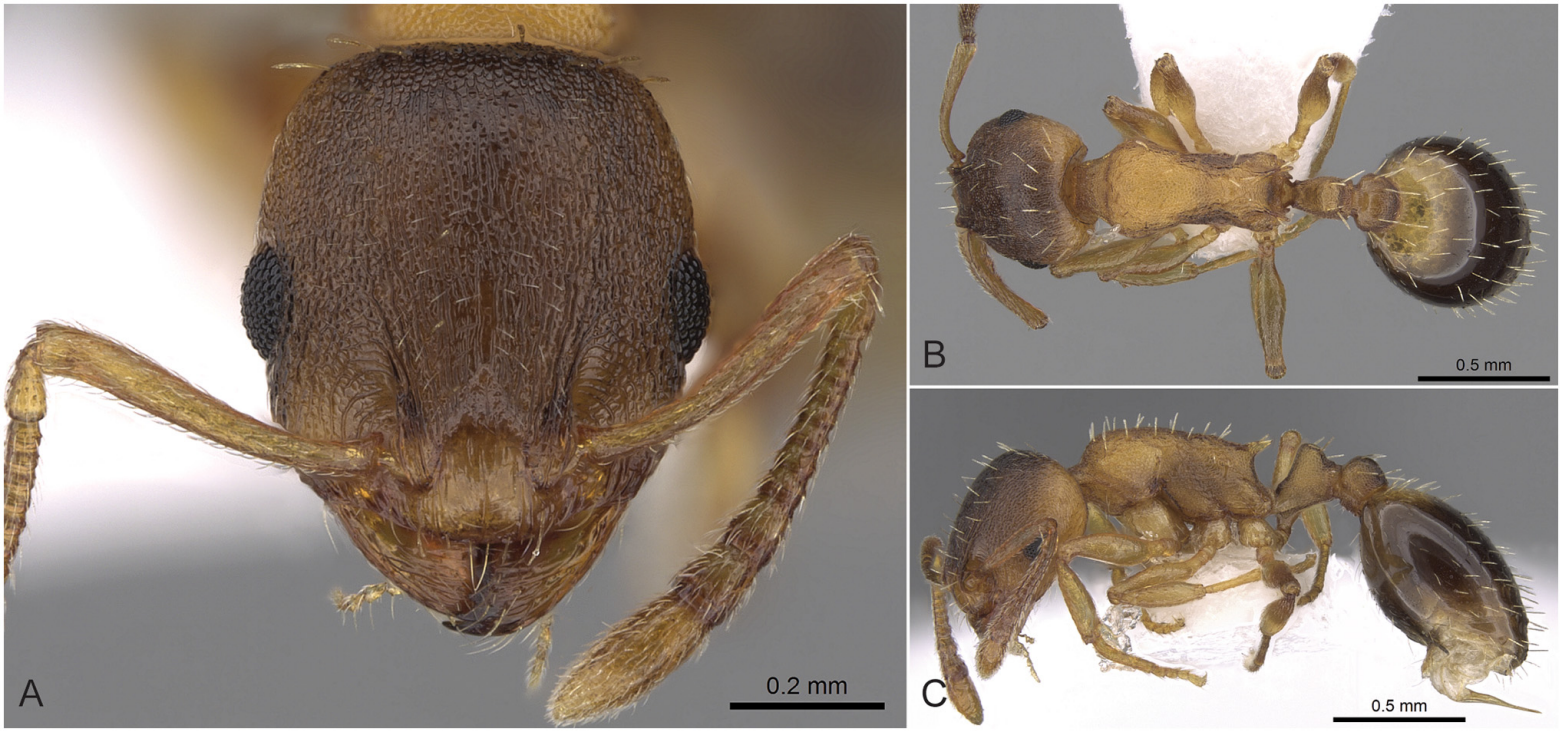
*Temnothorax nylanderi* non-type worker. Head of the holotype worker (CASENT0906039) in full face view (A), dorsal view of the body (B), lateral view of the body (C).

#### Differential diagnosis

*Temnothorax nylanderi* has moderately long spines (SPST/CS) and therefore cannot be confused with long-spined *T*. *lichtensteini*. Non-overlapping SPBA/CS ratios help to distinguish it from *T*. *flavicornis* (S4 Table). Based on salient features, *T*. *nylanderi* might be misidentified as *T*. *parvulus*, but a simple ratio (SPTI/CS) reliably separate these species on the level of nest sample means (S4 Table). *Temnothorax nylanderi* can also be safely separated from weakly sculptured, lightly colored *T*. *tergestinus* using the ratios PoOC/CL and SPWI/CS (S4 Table).

#### Geographic distribution

This species occurs in West Europe (Fig 19).

### Diagnosis of *Temnothorax parvulus* species-complex

Workers of the *Temnothorax parvulus* species-complex can be distinguished from those of other complexes treated in this revision by the combination of the following salient features: dirty yellowish to brownish color; slightly longer than broad head (CL/CWb [1.147, 1.242]), sculpture of head dorsum dull: with uniformly areolate ground sculpture combined with inconspicuous (or the lack of) main sculpture; short to long propodeal spines (SPST/CS [0.205, 0.331]), deviating from longitudinal axis of mesosoma by 38–42°; petiolar node in lateral view with a concave frontal profile meeting truncate dorsum in a right angle to an obtuse angle (100–110°) with a narrowly rounded transition, without a conspicuous sharp fronto-dorsal ridge on the petiolar node.

**Fig 23:**
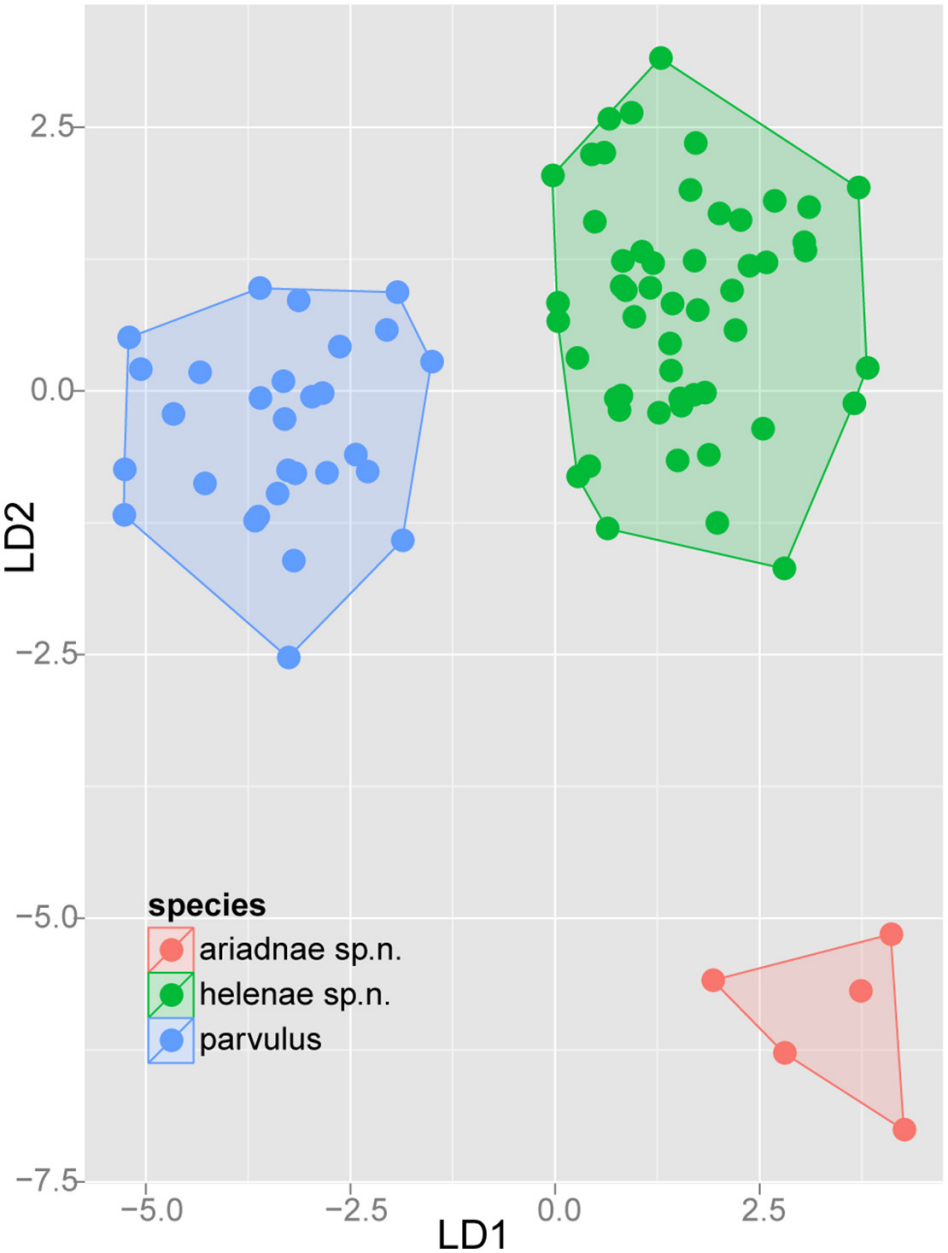
Scatterplot of discriminant scores for *Temnothorax parvulus* species-complex is illustrated on the 1^st^ and 2^nd^ axis. Color codes: *T*. *ariadnae* sp.n. (red), *T*. *helenae* sp.n. (green), *T*. *parvulus* (blue). Convex hulls visualize the range for each group.

**Fig 24:**
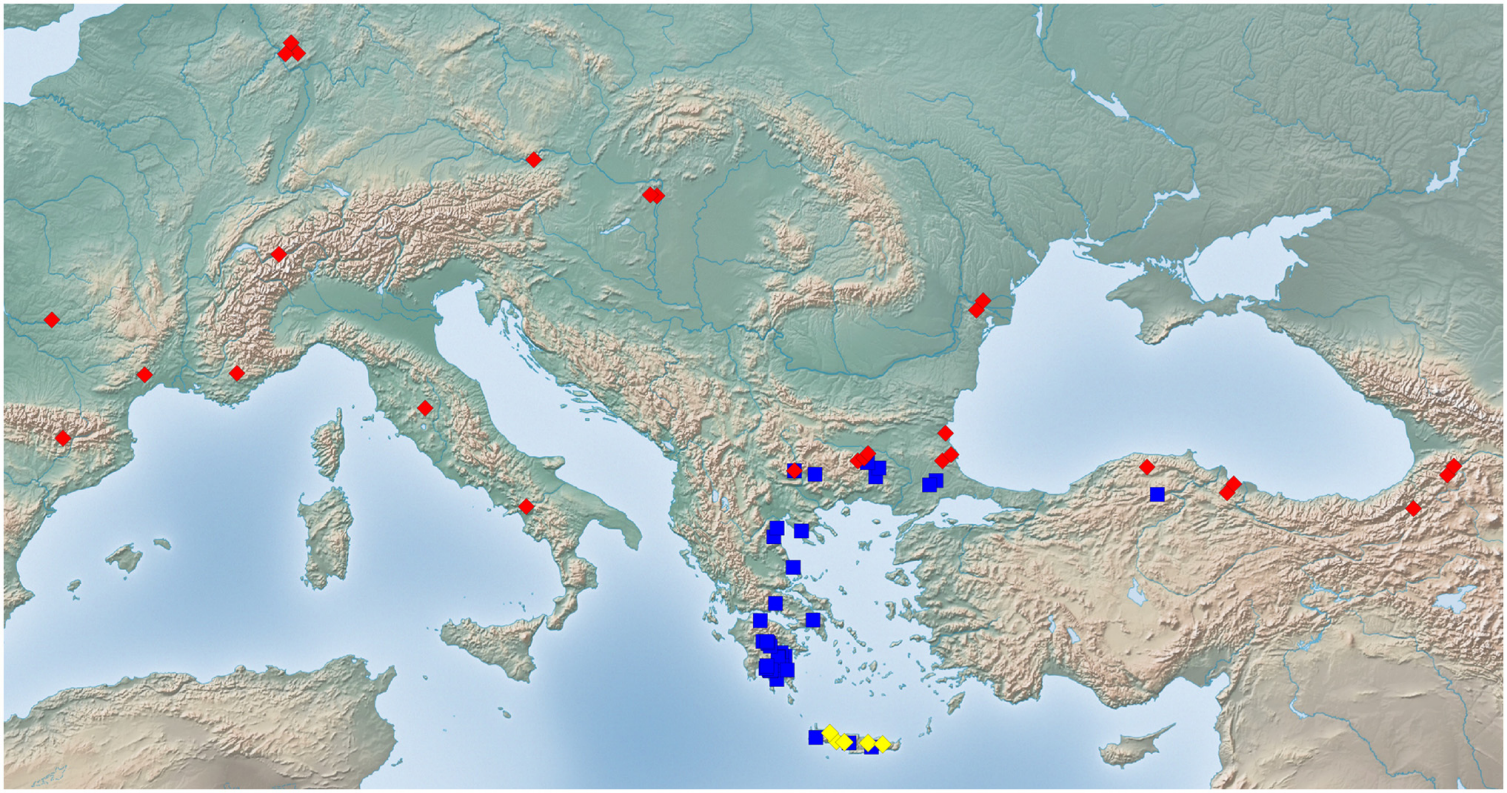
Sampling sites of *Temnothorax parvulus* species-complex. Color codes: *T*. *ariadnae* sp.n. (yellow diamonds), *T*. *helenae* sp.n. (blue rectangles), *T*. *parvulus* (red diamonds).

Exploratory NC-clustering revealed the existence of three species, *Temnothorax ariadnae* sp. n., *T*. *helenae* sp. n., and *T*. *parvulus* (Schenck, 1852), which was corroborated by confirmatory analyses (S5 Table, Fig 23). Members of this species-complex are known to occur in Europe, the Caucasus, and Turkey (Fig 24).

### *Temnothorax ariadnae* sp. n.

urn:lsid:zoobank.org:act:47CD579F-EF1A-40CF-BBFD-C67224733C2D

#### Etymology

The name of this Cretan endemic species is dedicated to Ariadne, the daughter of king of Crete, Minos. In Greek mythology, her figure is closely connected with Minotaur’s labyrinth in Crete.

#### Type material investigated

**Holotype worker labelled:** GER:024 Greece, Crete, 5 km N Ano Vianos, Vic. Katofigi, 35.0922 N, 25.4165 E, 60 mH, 17.04.2011, leg. A. Schulz, (HNHM), [**GRE:Crete-Ano-Vianos-5N-20110417-024**];

**Paratypes:** GER:024 Greece, Crete, 5 km N Ano Vianos, Vic. Katofigi, 35.0922 N, 25.4165 E, 60 mH, 17.04.2011, leg. A. Schulz, (5## HNHM, 2## CAS, CASENT0914694), **[GRE:Crete-Ano-Vianos-5N-20110417-024]**; GRE:092 Greece, Crete, 3 km E Ag. Vasilios, 25 km S Rethimnon, 35.2408 N, 24.4652 E, 300mH, 24.04.2011, leg. A. Schulz, (5## HNHM), **[GRE:Crete-Ag-Vasilios-3E-20110424-092]**; GRE:093 Greece, Crete, 3 km E Ag. Vasilios, 25 km S Rethimnon, 35.2408 N, 24.4652 E, 300mH, 24.04.2011, leg. A. Schulz, (3## HNHM, 2## CAS CASENT0906020, CASENT0906678), **[GRE:Crete-Ag-Vasilios-3E-20110424-093]**; GRE:037 Greece, Crete, Lassithi Plateau, 16 km S Malia, 35.1623 N, 25.4560 E, 1000mH, 18.04.2011, leg.A. Schulz, (3## HNHM, 1# CAS), **[GRE:Crete-Malia16S-20110418-037]**;

Locality data of the single non-type sample with 3 individuals investigated is given in S1 Table.

#### Worker (Fig 25A–25C, S1 Table, S4 Table)

Body color: brown; yellow. Body color pattern: mesosoma, antenna and legs, waist and anterior region of 1st gastral tergite lighter than head dorsum and posterior region of gaster. Antenna color pattern: clava concolorous funicle. Absolute cephalic size: 533–557 μm (mean = 543, n = 5). Cephalic length vs. Maximum width of head capsule (CL/CWb): 1.196–1.293 (mean = 1.223). Postocular distance vs. cephalic length (PoOc/CL): 0.373–0.393 (mean = 0.386). Postocular sides of cranium contour frontal view orientation: converging posteriorly. Postocular sides of cranium contour frontal view shape: strongly convex. Vertex contour line in frontal view shape: straight. Vertex sculpture: main sculpture absent, ground sculpture areolate. Genae contour from anterior view orientation: converging. Gena contour line in frontal view shape: convex. Gena sculpture: rugoso-reticulate with areolate ground sculpture. Median region of antennal rim vs. frontal carina in frontal view structure: not fully overlapped by frontal carina. Concentric carinae laterally surrounding antennal foramen count: present. Eye length vs. absolute cephalic size (EL/CS): 0.260–0.266 (mean = 0.263). Frontal carina distance vs. absolute cephalic size (FRS/CS): 0.346–0.369 (mean = 0.356). Longitudinal carinae on median region of frons count: absent. Smooth median region on frons count: absent. Antennomere count: 12. Scape length vs. absolute cephalic size (SL/CS): 0.758–0.795 (mean = 0.775). Facial area of the scape absolute setal angle: 0–15°. External area of the scape absolute setal angle: 30°; 35–45°. Ground sculpture of submedian area of clypeus: smooth. Median carina of clypeus count: present. Lateral carinae of clypeus count: present. Median anatomical line of propodeal spine angle value to Weber length in lateral view: 45–50°. Spine length vs. absolute cephalic size (SPST/CS): 0.220–0.249 (mean = 0.237). Minimum spine distance vs. absolute cephalic size (SPBA/CS): 0.248–0.287 (mean = 0.278). Maximum spine distance vs. absolute cephalic size (SPWI/CS): 0.303–0.336 (mean = 0.314). Apical spine distance vs. absolute cephalic size (SPTI/CS): 0.289–0.322 (mean = 0.300). Maximum mesosoma width vs. absolute cephalic size (MW/CS): 0.602–0.618 (mean = 0.611). Metanotal depression count: present. Metanotal depression shape: shallow. Dorsal region of mesosoma sculpture: rugulose with areolate ground sculpture. Lateral region of pronotum sculpture: areolate ground sculpture, main sculpture forked costate. Mesopleuron sculpture: areolate ground sculpture superimposed by dispersed rugulae. Metapleuron sculpture: areolate ground sculpture superimposed by dispersed rugulae. Frontal profile of petiolar node contour line in lateral view shape: concave. Anterodorsal rim of petiole count: absent medially. Dorsal profile of petiolar node contour line in lateral view shape: strongly convex. Dorsal region of petiole sculpture: ground sculpture areolate, main sculpture dispersed rugose; ground sculpture areolate, main sculpture absent. Dorso-caudal petiolar profile contour line in lateral view shape: straight; concave. Dorsal region of postpetiole sculpture: ground sculpture areolate, main sculpture dispersed rugose; ground sculpture areolate, main sculpture absent.

**Fig 25:**
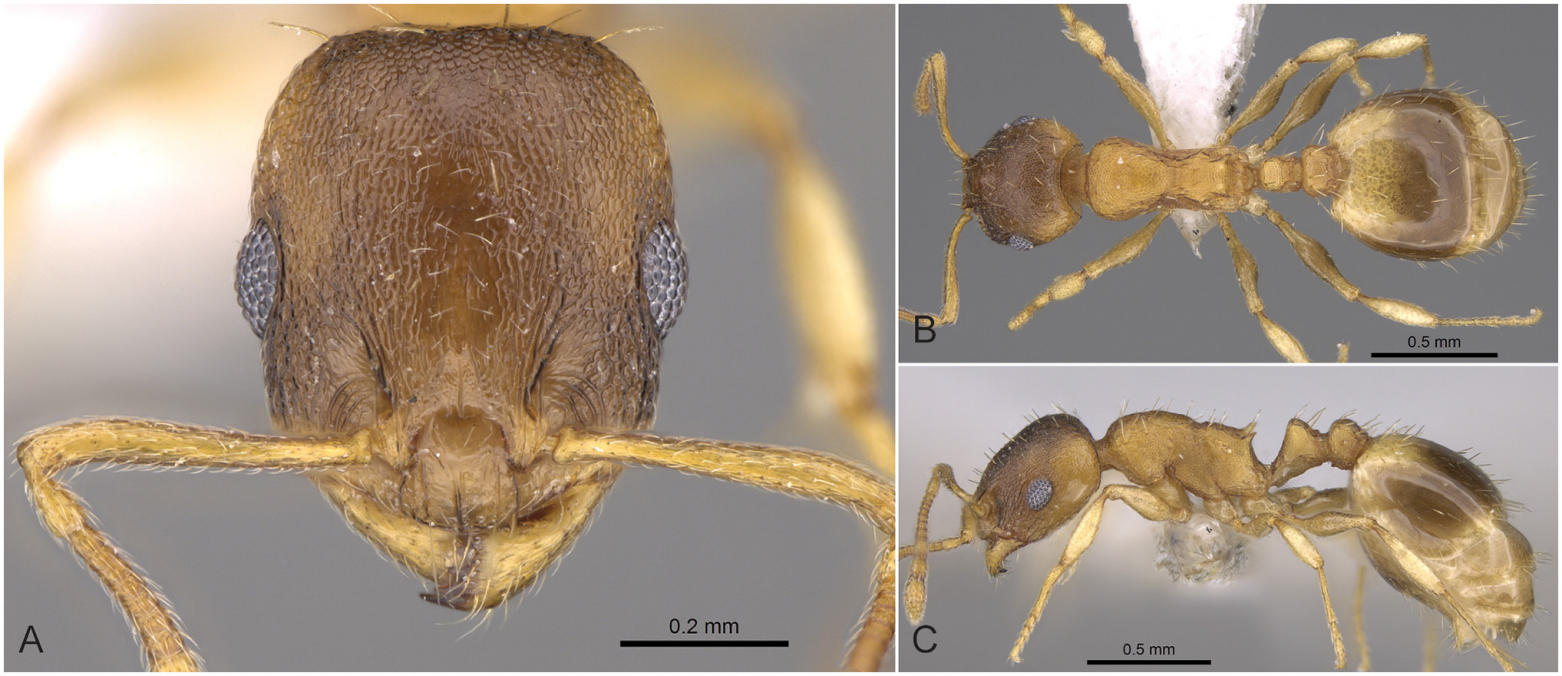
*Temnothorax ariadnae* sp.n. paratype worker. Head of paratype worker (CASENT0914694) in full face view (A), dorsal view of the body (B), lateral view of the body (C).

#### Differential diagnosis

This species shares most characteristics of its surface sculpture and shape with *T*. *helenae* sp. n. and *T*. *parvulus*. Values of the propodeal spine length ratio (SPST/CL) for nest sample means perfectly separate *T*. *parvulus* (mean: 0.282 [min: 0.259, max: 0.303]) and *T*. *ariadnae* sp. n. (mean: 0.216 [min: 0199, max: 0.227]). The geographical range of *T*. *ariadnae* sp. n. and *T*. *parvulus* (Fig 24) does not overlap and more complicated means of separating single individuals are therefore not needed.

The separation of *T*. *ariadnae* sp. n. and *T*. *helenae* sp. n. can be more difficult, because both taxa co-occur in Crete and various body ratios overlap. A discriminant (D4 = -0.0932*PPL -0.0767*POC +0.1203*PL -0.0384*SPWI +9.827) function is the shortest formula that yields reliable separation for single individuals (99.4%) and nest sample means (100%) of *T*. *ariadnae* sp. n. and *T*. *helenae* sp. n.

D4 scores for single individuals:

*T*. *ariadnae* sp. n. (n 15) = +1.607 [+0.042, 2.571], [5–95% percentiles: +0.042, +2,571]

*T*. *helenae* sp. n. (n 169) = -1.607 [-4.748, +0746], [5–95% percentiles: -3.333, -0.061]

D4 scores for nest sample means:

*T*. *ariadnae* sp.n. (n 5) = +1.607 [+1.230, +1.960]

*T*. *helenae* sp.n. (n 53) = -1.678 [-3.899, -0.119]

*Temnothorax ariadnae* sp. n. can be easily separated from two additional species whose distributional range expands to Crete, *T*. *lucidus* sp. n. and *T*. *subtilis* sp. n., based on the shiny surface of the head dorsum of the two latter. In exceptional cases, or if dust cover obstructs a clear view of the surface sculpture, body ratios help to distinguish *T*. *ariadnae* sp. n. from *T*. *subtilis* sp. n. by the longer head (CL/CWb), the larger eyes (EL) and the longer propodeal spines (SPST/CS) and from *T*. *lucidus* sp. n. by the non-overlapping NodL/CS, lower NOL/CS ratio and the longer head (S4 Table).

#### Geographic distribution

This species is endemic to Crete (Fig 24).

### *Temnothorax helenae* sp. n.

urn:lsid:zoobank.org:act:547C85B9-F6C0-424B-96C9-0A813EC152AA

#### Etymology

This species is named after Helena, a figure of the Greek mythology.

#### Type material investigated

**Holotype worker labelled:** GRE:344 Greece, Peloponnesus, Taygetos Oros, Street to Profitis Elias, 36.968 N, 22.404 E, 800mH, 01.05.2011, leg. A. Schulz, (HNHM, CASENT0914697), [**GRE:Taigetos-Oros-20110501-344]**;

**Paratypes:** GRE:344 Greece, Peloponnesus, Taygetos Oros, Street to Profitis Elias, 36.968 N, 22.404 E, 800mH, 01.05.2011, leg. A. Schulz, (6## HNHM), [**GRE:Taigetos-Oros-20110501-344**]; GRE:267 Greece, Peloponnesus, Taygetos Oros, Trail to Profitis Elias, 36.960 N, 22.396 E, 1000-1200mH, 30.04.2011, leg. A. Schulz, (3## HNHM, 2## CAS), [**GRE:Taigetos-Oros-20110430-267**]; GRE:280 Greece, Peloponnesus, Taygetos Oros, Trail to Profitis Elias, 36.960 N, 22.396 E, 1000-1200mH, 30.04.2011, leg. A. Schulz, (5## HNHM), [**GRE:Taigetos-Oros-20110430-280**]; GRE:287 Greece, Peloponnesus, Taygetos Oros, Trail to Profitis Elias, 36.948 N, 22.377 E, 1400-1600mH, 30.04.2011, leg. A. Schulz, (2## HNHM, 2## CAS), [**GRE:Taigetos-Oros-20110430-287**]; GRE:348 Greece, Peloponnesus, Taygetos Oros, Street to Profitis Elias, 36.968 N, 22.404 E, 800mH, 01.05.2011, leg. A. Schulz, (5## HNHM, 2## CAS), [**GRE:Taigetos-Oros-20110501-348**]; GRE:350 Greece, Peloponnesus, Taygetos Oros, Street to Profitis Elias, 36.968 N, 22.404 E, 800mH, 01.05.2011, leg. A. Schulz, (5## HNHM), [**GRE:Taigetos-Oros-20110501-350**];

The list of 154 individuals belonging to 43 nest samples of other material investigated is given in S1 Table.

#### Worker (Fig 26A–26C, S1 Table, S4 Table)

Body color: brown; yellow. Body color pattern: mesosoma, antenna and legs, waist and anterior region of 1st gastral tergite lighter than head dorsum and posterior region of gaster. Antenna color pattern: clava concolorous funicle. Absolute cephalic size: 510–627 μm (mean = 566, n = 53). Cephalic length vs. Maximum width of head capsule (CL/CWb): 1.152–1.242 (mean = 1.197). Postocular distance vs. cephalic length (PoOc/CL): 0.384–0.424 (mean = 0.400). Postocular sides of cranium contour frontal view orientation: converging posteriorly. Postocular sides of cranium contour frontal view shape: feebly convex. Vertex contour line in frontal view shape: straight. Vertex sculpture: main sculpture absent, ground sculpture areolate; main sculpture dispersed forked costate sculpture, ground sculpture areolate. Genae contour from anterior view orientation: converging. Gena contour line in frontal view shape: feebly convex. Gena sculpture: rugoso-reticulate with areolate ground sculpture. Median region of antennal rim vs. frontal carina in frontal view structure: not fully overlapped by frontal carina. Concentric carinae laterally surrounding antennal foramen count: present. Eye length vs. absolute cephalic size (EL/CS): 0.238–0.270 (mean = 0.251). Frontal carina distance vs. absolute cephalic size (FRS/CS): 0.335–0.373 (mean = 0.357). Longitudinal carinae on median region of frons count: absent; present. Longitudinal carinae on medial region of frons shape: forked. Smooth median region on frons count: present; absent. Antennomere count: 12. Scape length vs. absolute cephalic size (SL/CS): 0.758–0.808 (mean = 0.783). Facial area of the scape absolute setal angle: 0–15°. External area of the scape absolute setal angle: 30°. Ground sculpture of submedian area of clypeus: smooth. Median carina of clypeus count: present. Lateral carinae of clypeus count: present. Median anatomical line of propodeal spine angle value to Weber length in lateral view: 45–50°. Spine length vs. absolute cephalic size (SPST/CS): 0.205–0.299 (mean = 0.255). Minimum spine distance vs. absolute cephalic size (SPBA/CS): 0.255–0.319 (mean = 0.281). Maximum spine distance vs. absolute cephalic size (SPWI/CS): 0.290–0.387 (mean = 0.334). Apical spine distance vs. absolute cephalic size (SPTI/CS): 0.277–0.370 (mean = 0.320). Maximum mesosoma width vs. absolute cephalic size (MW/CS): 0.580–0.634 (mean = 0.611). Metanotal depression count: present. Metanotal depression shape: shallow. Dorsal region of mesosoma sculpture: rugulose with areolate ground sculpture. Lateral region of pronotum sculpture: areolate ground sculpture, main sculpture forked costate. Mesopleuron sculpture: areolate ground sculpture superimposed by dispersed rugulae. Metapleuron sculpture: areolate ground sculpture superimposed by dispersed rugulae. Frontal profile of petiolar node contour line in lateral view shape: concave. Dorsal profile of petiolar node contour line angle value to frontal profile of petiole contour line in lateral view: 100–110°. Anterodorsal rim of petiole count: absent medially. Dorsal profile of petiolar node contour line in lateral view shape: slightly convex to conspicuously rounded. Dorsal region of petiole sculpture: ground sculpture areolate, main sculpture dispersed rugose; ground sculpture areolate, main sculpture absent. Dorso-caudal petiolar profile contour line in lateral view shape: straight; concave. Dorsal region of postpetiole sculpture: ground sculpture areolate, main sculpture dispersed rugose; ground sculpture areolate, main sculpture absent.

**Fig 26:**
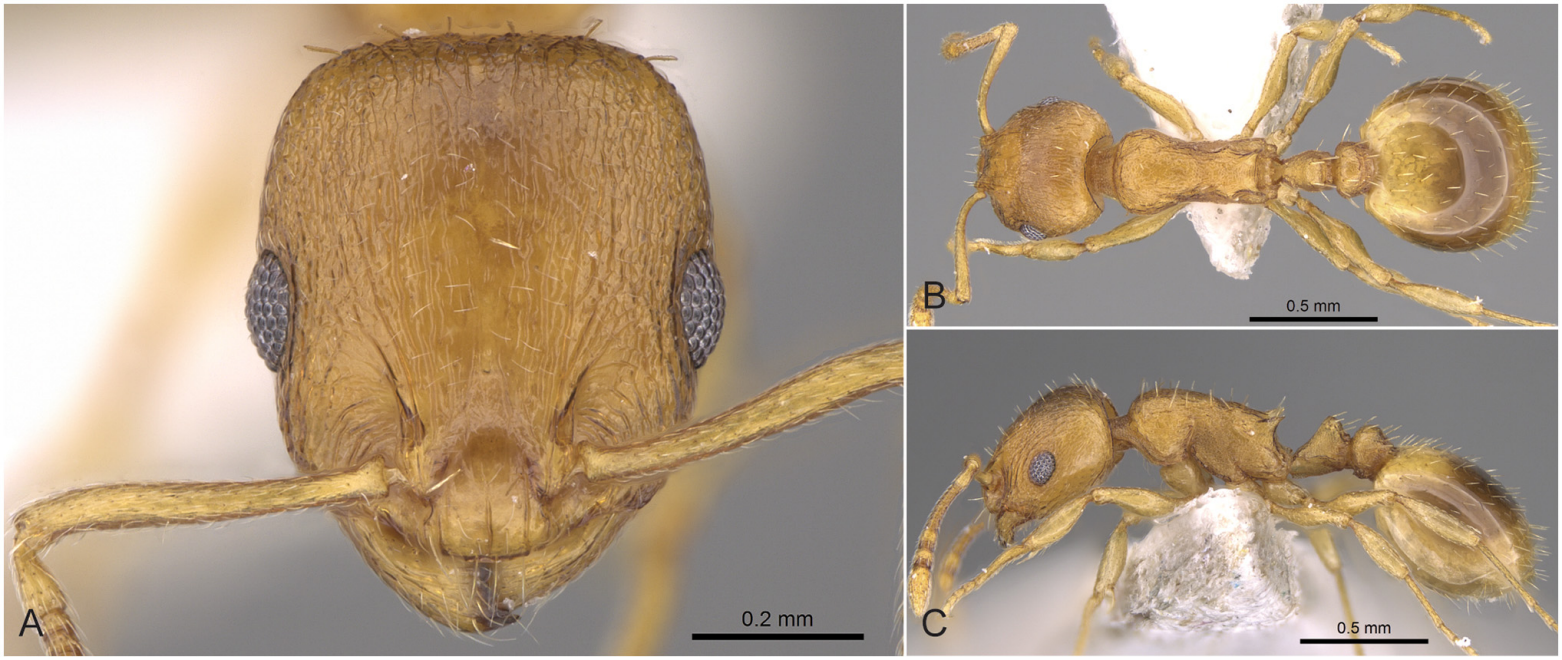
*Temnothorax helenae* sp.n. holotype worker. Head of the holotype worker (CASENT0914697) in full face view (A), dorsal view of the body (B), lateral view of the body (C).

#### Differential diagnosis

*Temnothorax helenae* sp. n. shares characters with *T*. *ariadnae* sp. n. and *T*. *parvulus*, differential diagnoses between these species are given under these taxa.

Separation of *T*. *helenae* sp. n. from *T*. *crasecundus* can be difficult if only sculpture and shape characteristics are considered, but a single ratio (PoOC/NOH) yields >95% classification success for nest sample means (see Fig 4). Several slightly overlapping morphometric traits (CL/CWb, ML/CS, NOH/CS, SL/CS see S4 Table) may provide further options for determination.

The geographical distribution of *T*. *helenae* sp. n. overlaps with those of *T*. *lucidus* sp. n. and *T*. *subtilis* sp. n. in a narrow zone in Greek mainland and in Crete. *Temnothorax helenae* sp. n. can be separated easily from the latter two species by the coarser sculpture on its head dorsum and the lack of a sharp antero-lateral ridge on its petiolar node of *T*. *helenae* sp. n. Both *T*. *lucidus* sp. n. and *T*. *subtilis* sp. n. have a shiny head dorsum and a sharp to moderately sharp antero-lateral ridge on the petiolar node. Due to the fact that head dorsum of *T*. *helenae* sp. n. is occasionally moderately shiny and the antero-lateral rim on the petiolar node may occasionally be inconspicuous in both *T*. *lucidus* sp. n. and *T*. *subtilis* sp., in a few cases determination might be doubtful. A discriminant (D5 = -0.0730*SPST +0.0255*SL -0.0634*POC +0.0405*ML -0.0613*SPBA -4.8074) function yields 97% classification success for individuals and 100% for nest sample means.

D5 scores for nest sample means:

*T*. *helenae* sp. n. (n = 53) = -2.209 [-4.220, -0.234]

*T*. *lucidus* sp. n. (n = 23) = +1.410 [+0.190, +2.314]

*T*. *subtilis* sp. n. (n = 55) = +1.579 [+0.218, +3.155]

#### Geographic distribution

This species mostly occurs in Greek mainland, but a few localities are also known in Southern Bulgaria, Western Turkey and Crete (Fig 24).

### *Temnothorax parvulus* (Schenck, 1852)

*Myrmica parvula* Schenck, 1852: 103 (w.) GERMANY. Palearctic

Combination in *Temnothorax*: Bolton, 2003: 271.

#### Type material investigated

**Lectotype:** "L. parvulus # Sch" and "Lectotype *Leptothorax parvulus* Schenck, 1852 det. A. Schulz & M. Verhaagh 1999" (1# / SMF), [**GER:parvulus-TYPE**];

The list of 103 individuals belonging to 27 nest samples of other material investigated is given in S1 Table.

#### Worker (Fig 27A–27C, S1 Table, S4 Table)

Body color: brown; yellow. Body color pattern: mesosoma, antenna and legs, waist and anterior region of 1st gastral tergite lighter than head dorsum and posterior region of gaster. Antenna color pattern: clava concolorous funicle. Absolute cephalic size: 488–586 μm (mean = 550, n = 29). Cephalic length vs. Maximum width of head capsule (CL/CWb): 1.147–1.214 (mean = 1.184). Postocular distance vs. cephalic length (PoOc/CL): 0.392–0.413 (mean = 0.405). Postocular sides of cranium contour frontal view orientation: converging posteriorly. Postocular sides of cranium contour frontal view shape: strongly convex. Vertex contour line in frontal view shape: straight. Vertex sculpture: main sculpture absent, ground sculpture areolate; main sculpture dispersed forked costate sculpture, ground sculpture areolate. Genae contour from anterior view orientation: converging. Gena contour line in frontal view shape: convex. Gena sculpture: rugoso-reticulate with areolate ground sculpture. Median region of antennal rim vs. frontal carina in frontal view structure: not fully overlapped by frontal carina. Concentric carinae laterally surrounding antennal foramen count: present. Eye length vs. absolute cephalic size (EL/CS): 0.237–0.262 (mean = 0.250). Frontal carina distance vs. absolute cephalic size (FRS/CS): 0.353–0.376 (mean = 0.361). Longitudinal carinae on median region of frons count: absent. Smooth median region on frons count: present. Antennomere count: 12. Scape length vs. absolute cephalic size (SL/CS): 0.763–0.796 (mean = 0.778). Facial area of the scape absolute setal angle: 0–15°. External area of the scape absolute setal angle: 30°. Ground sculpture of submedian area of clypeus: smooth. Median carina of clypeus count: present. Lateral carinae of clypeus count: present. Median anatomical line of propodeal spine angle value to Weber length in lateral view: 38–42°. Spine length vs. absolute cephalic size (SPST/CS): 0.278–0.331 (mean = 0.306). Minimum spine distance vs. absolute cephalic size (SPBA/CS): 0.273–0.312 (mean = 0.292). Maximum spine distance vs. absolute cephalic size (SPWI/CS): 0.353–0.415 (mean = 0.384). Apical spine distance vs. absolute cephalic size (SPTI/CS): 0.332–0.395 (mean = 0.364). Maximum mesosoma width vs. absolute cephalic size (MW/CS): 0.599–0.636 (mean = 0.618). Metanotal depression count: present. Metanotal depression shape: shallow. Dorsal region of mesosoma sculpture: rugulose with areolate ground sculpture. Lateral region of pronotum sculpture: areolate ground sculpture, main sculpture forked costate. Mesopleuron sculpture: areolate ground sculpture superimposed by dispersed rugulae. Metapleuron sculpture: areolate ground sculpture superimposed by dispersed rugulae. Frontal profile of petiolar node contour line in lateral view shape: concave. Dorsal profile of petiolar node contour line angle value to frontal profile of petiole contour line in lateral view: 105–110°. Anterodorsal rim of petiole count: absent medially. Dorsal profile of petiolar node contour line in lateral view shape: slightly convex. Dorsal region of petiole sculpture: ground sculpture areolate, main sculpture dispersed rugose; ground sculpture areolate, main sculpture absent. Dorso-caudal petiolar profile contour line in lateral view shape: straight; concave. Dorsal region of postpetiole sculpture: ground sculpture areolate, main sculpture dispersed rugose; ground sculpture areolate, main sculpture absent.

**Fig 27:**
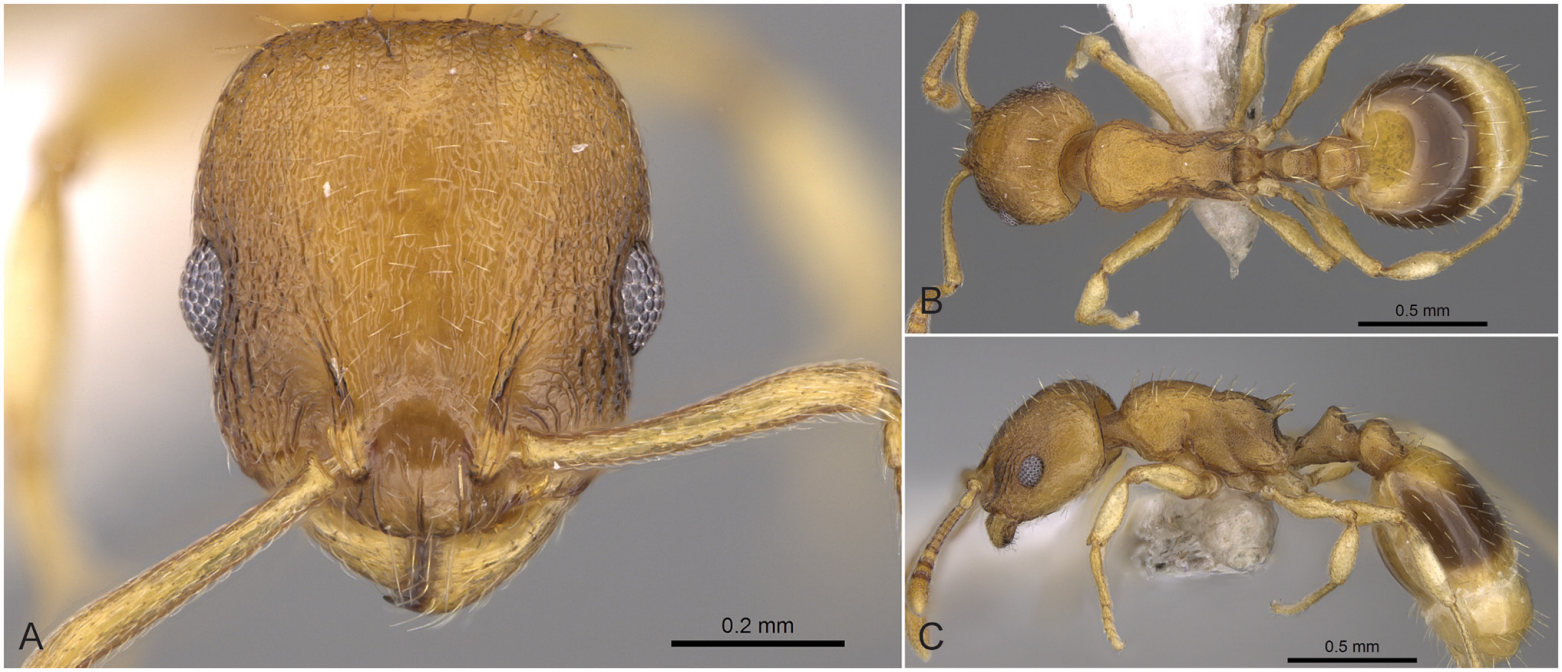
*Temnothorax parvulus* non-type worker. Head of the holotype worker (CASENT0914689) in full face view (A), dorsal view of the body (B), lateral view of the body (C).

#### Differential diagnosis

*Temnothorax parvulus* can be separated easily from members of other species-complexes by its homogenously areolate microsculpture on the head dorsum. The head sculpture of other species complexes may vary from smooth to coarsely rugulo-reticulate but is never homogenously areolate. In exceptional cases, long-spined species of the *T*. *lichtensteini* complex might exhibit a homogenous areolate sculpture on an extended area of the head dorsum, but *T*. *parvulus* is distinguished from *T*. *lichtensteini* and *T*. *laconicus* by its more erect propodeal spines (38–42° vs. ca. 20–25°). *Temnothorax parvulus* can also be safely separated from weakly sculptured, lightly colored *T*. *tergestinus* samples using slightly overlapping NOH/CS, SPTI/CS and SPWI/CS ratios (see S4 Table).

*Temnothorax parvulus* shares most its surface sculpture characters with other species belonging to this complex, *T*. *ariadnae* sp. n. and *T*. *helenae* sp. n. Spine length ratio (SPST/CL) helps distinguish *Temnothorax parvulus* from *T*. *ariadnae* sp. n. and *T*. *helenae* sp. n.

Non-overlapping values of SPST/CL for nest sample means of *T*. *parvulus* (mean: 0.282 [min: 0.259, max: 0.303]); and *T*. *ariadnae* sp. n. (mean: 0.216 [min: 0199, max: 0.227]) provide a prefect means of separation. As 5% of nest samples of *T*. *parvulus* and *T*. *helenae* sp. n. (mean: 0.234 [min: 0.189, max: 0.274] overlap in this ratio, the safest determination is provided by a discriminant (D5 = 0.0393*CL -0.0616*SPST +0.0892*PEW -0.0580*PPW -0.0338*SPWI -8.7356) formula, which separates 97.1% of single individuals and all nest samples of *T*. *parvulus* and *T*. *helenae* sp. n.

D5 scores for single individuals:

*T*. *parvulus* (n 107) = -1.624 [-3.809, +0.811]

*T*. *helenae* sp.n. (n 169) = +1.647 [-0.950, +5.013]

D5 scores for nest sample means:

*T*. *parvulus* (n 29) = -1.723 [-2.921, -0.104]

*T*. *helenae* sp.n. (n 53) = +1.684 [+0.018, +3.937]

#### Geographic distribution

The known distribution of this species ranges from Western Europe to the Black see coast and Turkey and from Italy and the Balkans to Central Europe (Fig 24).

### Diagnosis of *Temnothorax sordidulus* species-complex

Workers of the *Temnothorax sordidulus* species-complex can be distinguished from those of other complexes treated in this revision by the combination of the following salient features: brown to black color; slightly longer than broad head (CL/CWb [1.143, 1.278)], sculpture of head dorsum dull: with areolate ground sculpture combined with conspicuous parallel costulate or irregular reticulate main sculpture; moderately long to long propodeal spines (SPST/CS [0.220, 0.335]), deviating from longitudinal axis of mesosoma by 40–50°; petiolar node in lateral view with a concave frontal profile meeting occasionally indistinct truncate dorsum in an obtuse angle (110–120°) with a narrowly rounded transition, without a conspicuous sharp fronto-dorsal ridge on the petiolar node. Four species consist of this complex: *Temnothorax artvinensis* Seifert, 2006, *T*. *schoedli* Seifert, 2006, *T*. *sordidulus* (Müller, 1923) and *T*. *tergestinus* (Finzi, 1928) stat.n.

**Fig 28:**
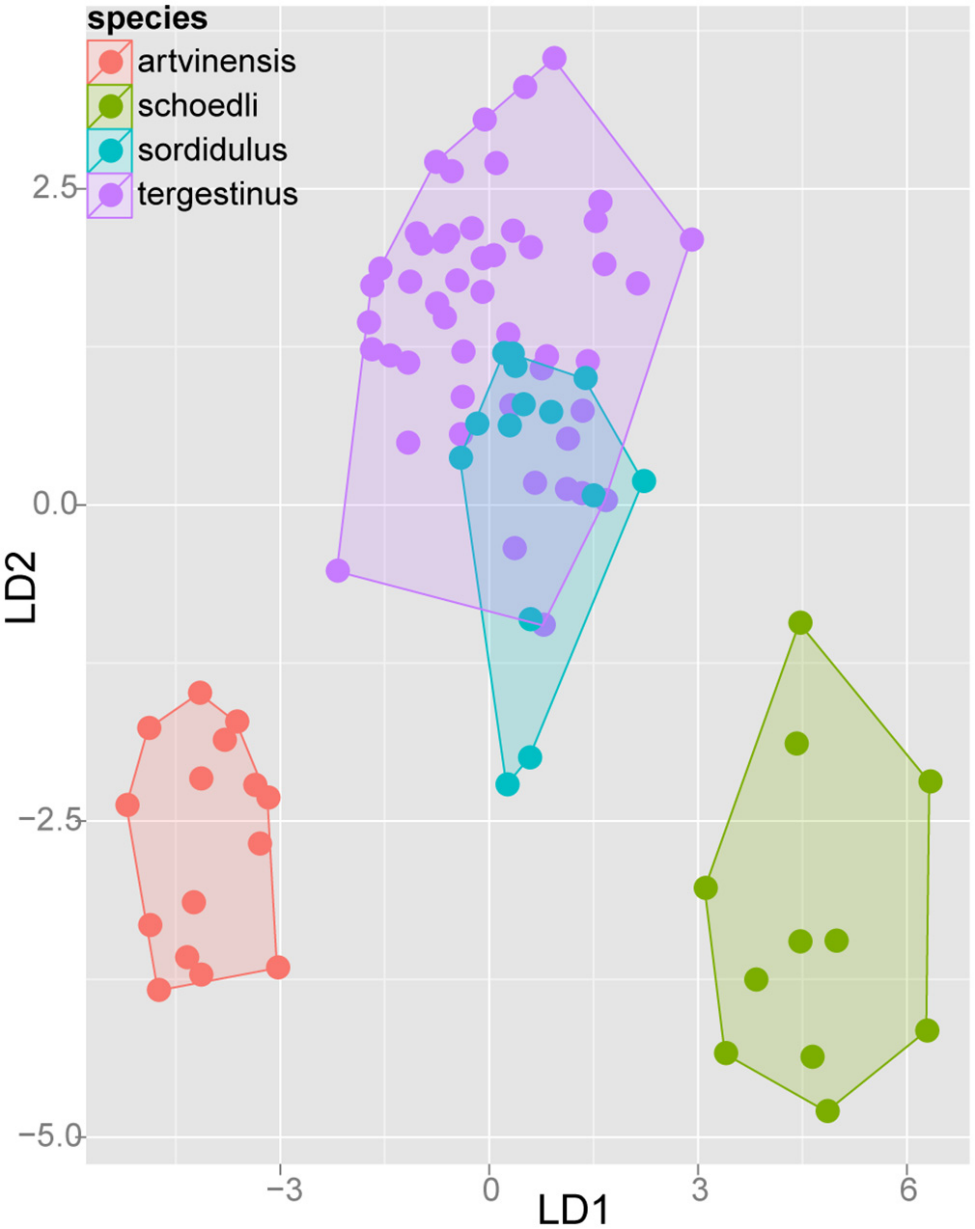
Scatterplot of discriminant scores for *Temnothorax sordidulus* species-complex is illustrated on the 1^st^ and 2^nd^ axis. Color codes: *T*. *artvinensis* (red), *T*. *schoedli* (green), *T*. *sordidulus* (blue) and *T*. *tergestinus* (lilla). Convex hulls visualize the range for each group.

**Fig 29:**
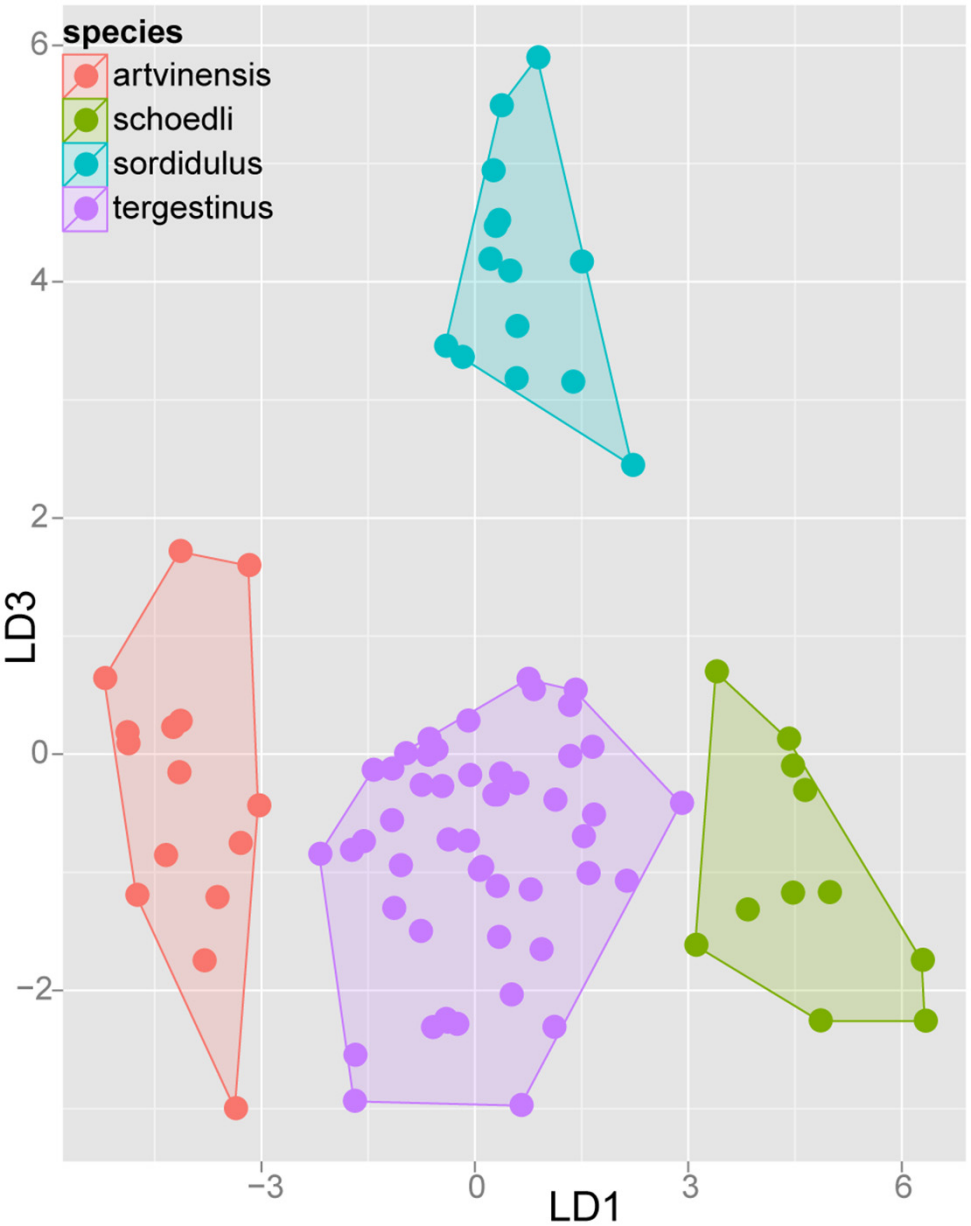
Scatterplot of discriminant scores for *Temnothorax sordidulus* species-complex is illustrated on the 1^st^ and 3^rd^ axis. Color codes: *T*. *artvinensis* (red), *T*. *schoedli* (green), *T*. *sordidulus* (blue) and *T*. *tergestinus* (lilla). Convex hulls visualize the range for each group.

**Fig 30:**
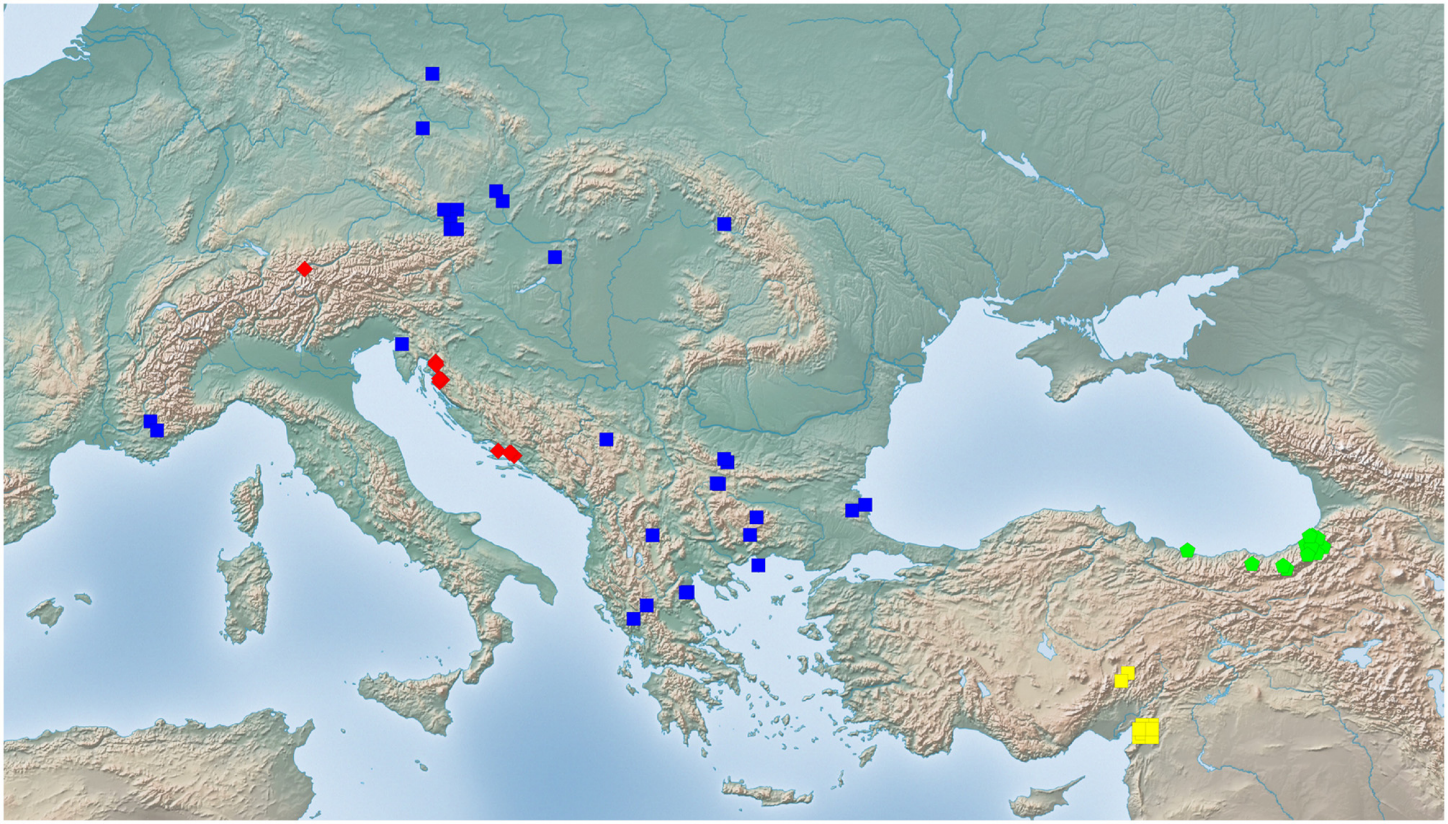
Sampling sites of *Temnothorax sordidulus* species-complex. Color codes: *T*. *artvinensis* (green pentagons), *T*. *schoedli* (yellow rectangles), *T*. *sordidulus* (red diamonds) and *T*. *tergestinus* (blue rectangles).

Exploratory NC-clustering revealed the existence of four species (*Temnothorax angustifrons* sp. n., *T*. *lucidus* sp. n., *T*. *similis* sp. n., *T*. *subtilis* sp. n.) within this complex, which was confirmed by LDA (Figs 28 and 29). Members of this species-complex are known to occur in Turkey and Crete. The sporadic occurrence of two samples in Greece may be ascribed to anthropochory (Fig 30).

### *Temnothorax artvinensis* Seifert, 2006

*Temnothorax artvinense* Seifert, 2006: 10 (w.q.) TURKEY.

#### Type material investigated

**Paratypes:** 9 paratype workers of 3 nest samples were investigated from the type locality: Turkey, Artvin, 5 km SW. Artvin, 41.1445 N, 41.8537 E, 1000 mH, 27.06.1993, leg. A. Schulz „no. 1162” (3## ASPC) [**TUR:Artvin-5SW-19930627-1162**]; „no. 1164” (3## ASPC) [**TUR:Artvin-5SW-19930627-1164**]; „no. 1165” (3## ASPC) [**TUR:Artvin-5SW-19930627-1165**];

The list of 39 individuals belonging to 12 nest samples of other material investigated is given in S1 Table.

#### Worker (Fig 31A–31C, S1 Table, S4 Table)

Body color: brown; black. Body color pattern: head, mesosoma, waist and anterior region of 1st gastral tergite lighter than antenna and legs and posterior region of gaster. Antenna color pattern: clava concolorous funicle. Absolute cephalic size: 540–634 μm (mean = 582, n = 15). Cephalic length vs. Maximum width of head capsule (CL/CWb): 1.206–1.263 (mean = 1.240). Postocular distance vs. cephalic length (PoOc/CL): 0.375–0.389 (0.384). Postocular sides of cranium contour frontal view orientation: parallel; converging posteriorly. Postocular sides of cranium contour frontal view shape: feebly convex. Vertex contour line in frontal view shape: straight. Vertex sculpture: main sculpture homogenously forked costate, ground sculpture areolate. Genae contour from anterior view orientation: converging. Gena contour line in frontal view shape: feebly convex. Gena sculpture: rugoso-reticulate with areolate ground sculpture. Median region of antennal rim vs. frontal carina in frontal view structure: not fully overlapped by frontal carina. Concentric carinae laterally surrounding antennal foramen count: present. Eye length vs. absolute cephalic size (EL/CS): 0.239–0.260 (mean = 0.247). Frontal carina distance vs. absolute cephalic size (FRS/CS): 0.357–0.375 (mean = 0.367). Longitudinal carinae on median region of frons count: present. Longitudinal carinae on medial region of frons shape: forked. Smooth median region on frons count: absent. Antennomere count: 12. Scape length vs. absolute cephalic size (SL/CS): 0.776–0.813 (mean = 0.794). Facial area of the scape absolute setal angle: 0–15°. External area of the scape absolute setal angle: 30°. Ground sculpture of submedian area of clypeus: carinate. Median carina of clypeus count: present. Lateral carinae of clypeus count: present. Median anatomical line of propodeal spine angle value to Weber length in lateral view: 42–48°. Spine length vs. absolute cephalic size (SPST/CS): 0.227–0.288 (mean = 0.265). Minimum spine distance vs. absolute cephalic size (SPBA/CS): 0.265–0.301 (mean = 0.278). Maximum spine distance vs. absolute cephalic size (SPWI/CS): 0.296–0.350 (mean = 0.331). Apical spine distance vs. absolute cephalic size (SPTI/CS): 0.280–0.337 (mean = 0.313). Maximum mesosoma width vs. absolute cephalic size (MW/CS): 0.598–0.626 (mean = 0.612). Metanotal depression count: present. Metanotal depression shape: deep. Dorsal region of mesosoma sculpture: rugulose with areolate ground sculpture. Lateral region of pronotum sculpture: areolate ground sculpture, main sculpture forked costate. Mesopleuron sculpture: areolate ground sculpture superimposed by dispersed rugulae. Metapleuron sculpture: areolate ground sculpture superimposed by dispersed rugulae. Frontal profile of petiolar node contour line in lateral view shape: concave. Dorsal profile of petiolar node contour line angle value to frontal profile of petiole contour line in lateral view: 110–120°. Anterodorsal rim of petiole count: absent medially. Dorsal profile of petiolar node contour line in lateral view shape: slightly convex. Dorsal region of petiole sculpture: ground sculpture areolate, main sculpture dispersed rugose; ground sculpture areolate, main sculpture absent. Dorso-caudal petiolar profile contour line in lateral view shape: concave; straight. Dorsal region of postpetiole sculpture: ground sculpture areolate, main sculpture dispersed rugose; ground sculpture areolate, main sculpture absent.

**Fig 31:**
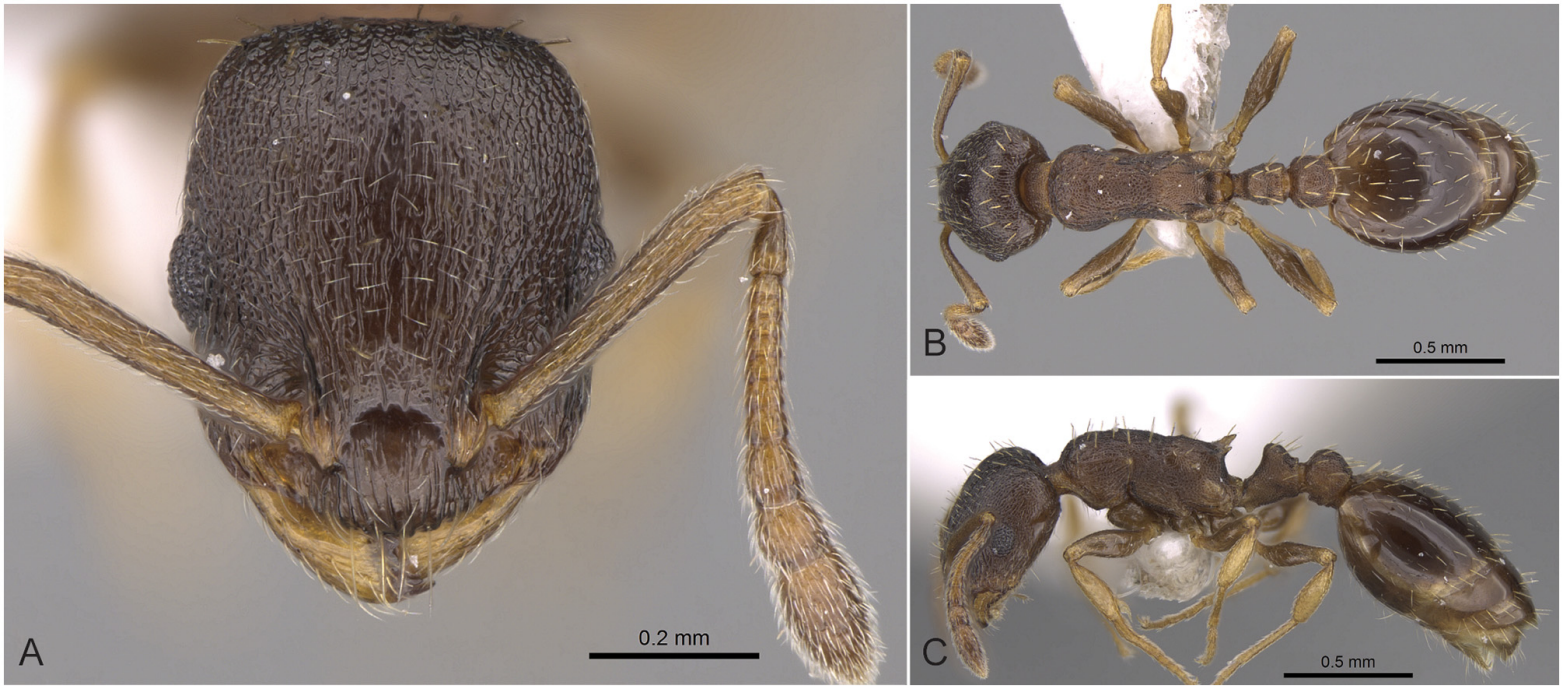
*Temnothorax artvinensis* non-type worker. Head of the holotype worker (CASENT0914702) in full face view (A), dorsal view of the body (B), lateral view of the body (C).

#### Differential diagnosis

The very dark color of *T*. *artvinensis* prevents confusion of this species with any other Turkish *Temnothorax* treated in this revision.

Due to the fact that *T*. *artvinensis* is geographically well isolated from other dark brown or black species of the *T*. *sordidulus* complex, it is unlikely that this this species is confused with its siblings. However, for doubtful cases, or if anthropochory is suspected, a discriminant function after character reduction (D4 = +0.114*EL -0.124*PEW +0.098*NOH -0.068*PPL +3.69209) separates 97.9% of *T*. *artvinensis* workers and each nest sample from that of Turkish *T*. *schoedli* and European brownish-black *T*. *sordidulus* and *T*. *tergestinus*.

D4 scores for nest sample means:

*T*. *artvinensis* (n 15) = -1.620 [-2.633, -0.877]

*T*. *schoedli* (*n 11*) = +2.044 [+1.296, +3.555]

*T*. *tergestinus* (*n 49*) = +1.575 [-0.377, +3.208]

*T*. *sordidulus* (*n 15*) = +2.218 [+0.620, +3.336]

#### Geographic distribution

This species occurs in a restricted area of North-East Turkish high mountains (Fig 30).

### *Temnothorax schoedli* Seifert, 2006

*Temnothorax schoedli* Seifert, 2006: 8 (w.q.) TURKEY.

#### Type material investigated

**Paratypes:** 20 paratype workers of 7 nest samples were investigated from the type locality: Turkey, Antakya, Nur Dağlari, 14 rkm W. Hassa 36.8414 N, 36.4309 E, 1600 mH, 11.05.1997, leg. A. Schulz, K. Vock, M. Sanetra: „no. 287” (2## SMNK) [**TUR:Hassa-14W-19970511-287**]; „no. 289” (3## SMNK) [**TUR:Hassa-14W-19970511-289**]; „no. 290” (3## SMNK) [**TUR:Hassa-14W-19970511-290**]; „no. 292” (3## ASPC) [**TUR:Hassa-14W-19970511-292**]; „no. 293” (3## ASPC) [**TUR:Hassa-14W-19970511-293**]; „no. 295” (3## ASPC) [**TUR:Hassa-14W-19970511-295**]; „no. 299” (3## ASPC) [**TUR:Hassa-14W-19970511-299**];

The list of 12 non-type individuals belonging to 4 nest samples of other material investigated is given in S1 Table.

#### Worker (Fig 32A–32C, S1 Table, S4 Table)

Body color: brown. Body color pattern: mesosoma, antenna and legs excluding femora, waist and anterior region of 1st gastral tergite lighter than head, femora and posterior region of gaster. Antenna color pattern: clava concolorous funicle. Absolute cephalic size: 572–696 μm (mean = 652, n = 11). Cephalic length vs. Maximum width of head capsule (CL/CWb): 1.143–1.196 (mean = 1.172). Postocular distance vs. cephalic length (PoOc/CL): 0.360–0.385 (mean = 0.368). Postocular sides of cranium contour frontal view orientation: converging posteriorly. Postocular sides of cranium contour frontal view shape: broadly convex. Vertex contour line in frontal view shape: straight. Vertex sculpture: main sculpture dispersed forked costate, ground sculpture inconspicuous areolate; main sculpture dispersed forked costate sculpture, ground sculpture areolate; main sculpture homogenously forked costate, ground sculpture areolate. Genae contour from anterior view orientation: converging. Gena contour line in frontal view shape: feebly convex. Gena sculpture: rugoso-reticulate with areolate ground sculpture. Median region of antennal rim vs. frontal carina in frontal view structure: not fully overlapped by frontal carina. Concentric carinae laterally surrounding antennal foramen count: present. Eye length vs. absolute cephalic size (EL/CS): 0.255–0.282 (mean = 0.268). Frontal carina distance vs. absolute cephalic size (FRS/CS): 0.363–0.379 (mean = 0.372). Longitudinal carinae on median region of frons count: present. Longitudinal carinae on medial region of frons shape: forked. Antennomere count: 12. Scape length vs. absolute cephalic size (SL/CS): 0.764–0.805 (mean = 0.784). Facial area of the scape absolute setal angle: 0–15°. External area of the scape absolute setal angle: 30°. Ground sculpture of submedian area of clypeus: smooth. Median carina of clypeus count: present. Lateral carinae of clypeus count: present. Median anatomical line of propodeal spine angle value to Weber length in lateral view: 40–45°. Spine length vs. absolute cephalic size (SPST/CS): 0.232–0.282 (mean = 0.261). Minimum spine distance vs. absolute cephalic size (SPBA/CS): 0.282–0.298 (mean = 0.288). Maximum spine distance vs. absolute cephalic size (SPWI/CS): 0.305–0.342 (mean = 0.327). Apical spine distance vs. absolute cephalic size (SPTI/CS): 0.293–0.328 (mean = 0.310). Maximum mesosoma width vs. absolute cephalic size (MW/CS): 0.613–0.650 (mean = 0.632). Metanotal depression count: present. Metanotal depression shape: shallow. Dorsal region of mesosoma sculpture: areolate ground sculpture, superimposed by dispersed rugae. Lateral region of pronotum sculpture: areolate ground sculpture, main sculpture dispersed costate. Mesopleuron sculpture: areolate ground sculpture superimposed by dispersed rugulae. Metapleuron sculpture: areolate ground sculpture superimposed by dispersed rugulae. Frontal profile of petiolar node contour line in lateral view shape: concave. Anterodorsal rim of petiole count: absent medially. Dorsal profile of petiolar node contour line in lateral view shape: widely rounded or slightly angulate area. Dorsal region of petiole sculpture: ground sculpture areolate, main sculpture dispersed rugose. Dorsal region of postpetiole sculpture: ground sculpture areolate, main sculpture dispersed rugose.

**Fig 32:**
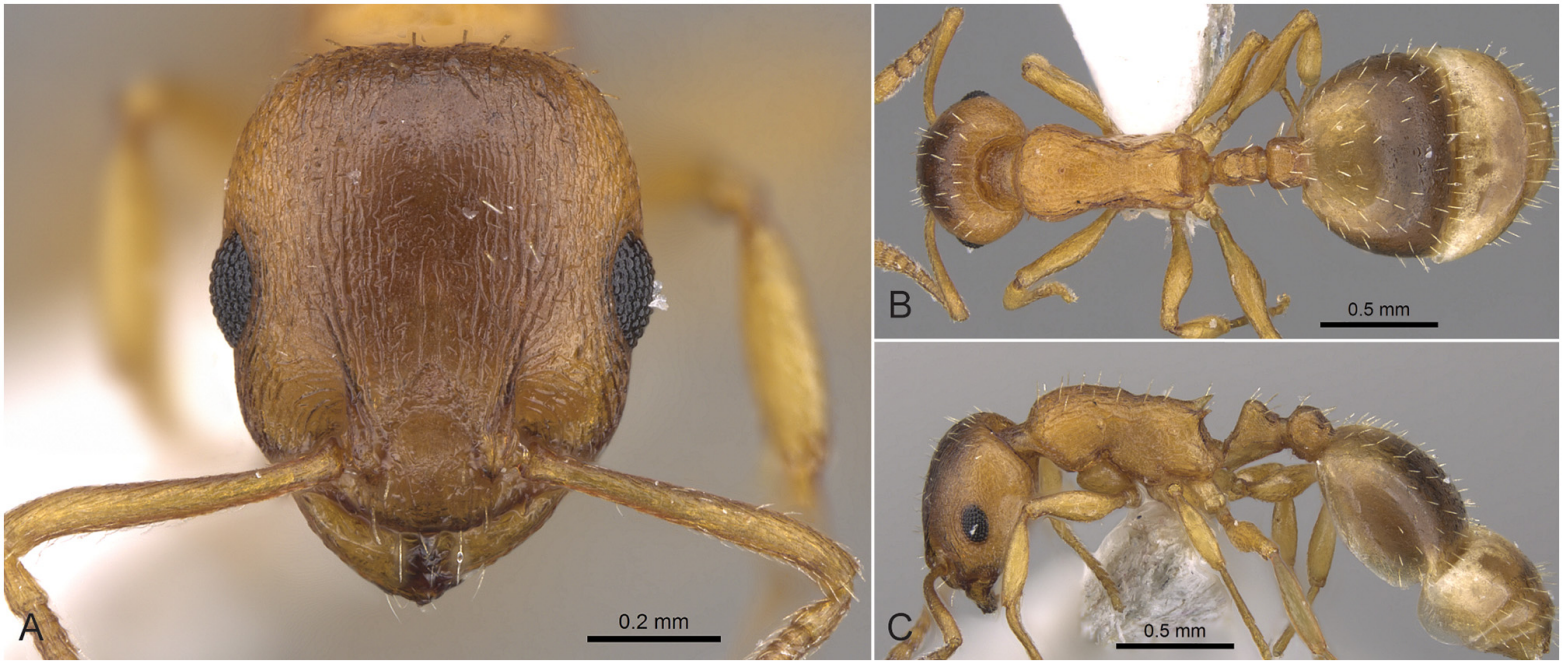
*Temnothorax schoedli* non-type worker. Head of the holotype worker (CASENT0914701) in full face view (A), dorsal view of the body (B), lateral view of the body (C).

#### Differential diagnosis

Among all species of the *T*. *sordidulus* species-complex, *Temnothorax schoedli* has the lightest color. In addition to color it can be separated safely from other members of the complex by its very restricted geographical occurrence in the southern ranges of Taurus Mountains in Turkey. *Temnothorax schoedli* can be separated from *T*. *artvinensis*, which in geographical terms is the most closely situated relative of this species, by the coarse dull head sculpture and the very dark brown to black color of the latter and its significantly shorter head. The non-overlapping CL/CWb ratio (see S4 Table) provides a perfect tool to separate of *T schoedli* from *T*. *artvinensis* at the level of nest sample means.

In its relatively lighter color and occasionally smooth head dorsum this species may superficially resemble species of *T*. *angustifrons* complex. The geographical range of *T*. *angustifrons* sp. n., *T*. *lucidus* sp. n., *T*. *similis* sp. n., and *T*. *subtilis* sp. n. broadly overlap with that of *T*. *schoedli*. However, nest samples of *T*. *schoedli* can be separated easily from those of these species by non-overlapping ratios (S4 Table): *T*. *schoedli* can be separated from *T*. *subtilis* sp. n. by the longer propodeal spines (SPST/CS); from *T*. *similis* sp. n. by PEH/CS and NOH/CS ratios; from *T*. *lucidus* sp. n. by SPBA/CS; and from *T*. *angustifrons* sp. n. by the FRS/CS ratio.

#### Geographic distribution

This species occurs in a restricted area of South-Eastern Turkish lowlands (Fig 30).

### *Temnothorax sordidulus* (Müller, 1923)

*Leptothorax sordidulus* Müller, 1923: 96 (w.) ITALY.

Combination in *Temnothorax*: Bolton, 2003: 271.

Senior synonym of *Temnothorax carinthiacus*: Schulz, 1991: 121.

#### Type material investigated

**Type material of *Leptothorax sordidulus* Müller, 1923:** not investigated, most probably lost, see also Seifert (2006).

**Lectotype and Paralectotype of *Leptothorax carinthiacus* Bernard, 1957:** Carinthia Viktring Hölzler leg. [on reverse side: „V/55”], Lectotype Leptothorax carinthiacus Bernard 1957 desig. B. Seifert 2006 (2# LMK), [**AUT:Viktring-carinthiacus-TYPE**] (CASENT0913637);

The list of 43 individuals belonging to 14 nest samples of other material investigated is given in S1 Table.

#### Worker (Fig 33A–33C, S1 Table, S4 Table)

Body color: brown; black. Body color pattern: head, mesosoma, waist and anterior region of 1st gastral tergite lighter than antenna and legs and posterior region of gaster. Antenna color pattern: clava concolorous funicle. Absolute cephalic size: 518–607μm (mean = 574, n = 15). Cephalic length vs. Maximum width of head capsule (CL/CWb): 1.192–1.278 (mean = 1.238). Postocular distance vs. cephalic length (PoOc/CL): 0.363–0.390 (mean = 0.374). Postocular sides of cranium contour frontal view orientation: parallel; converging posteriorly. Postocular sides of cranium contour frontal view shape: feebly convex. Vertex contour line in frontal view shape: straight. Vertex sculpture: main sculpture homogenously forked costate, ground sculpture areolate. Genae contour from anterior view orientation: converging. Gena contour line in frontal view shape: feebly convex. Gena sculpture: rugoso-reticulate with areolate ground sculpture. Median region of antennal rim vs. frontal carina in frontal view structure: not fully overlapped by frontal carina. Concentric carinae laterally surrounding antennal foramen count: present. Eye length vs. absolute cephalic size (EL/CS): 0.258–0.280 (mean = 0.266). Frontal carina distance vs. absolute cephalic size (FRS/CS): 0.353–0.378 (mean = 0.366). Longitudinal carinae on median region of frons count: present. Longitudinal carinae on medial region of frons shape: forked. Smooth median region on frons count: absent. Antennomere count: 12. Scape length vs. absolute cephalic size (SL/CS): 0.763–0.809 (mean = 0.785). Facial area of the scape absolute setal angle: 0–15°. External area of the scape absolute setal angle: 30°. Ground sculpture of submedian area of clypeus: smooth; carinate. Median carina of clypeus count: present. Lateral carinae of clypeus count: present. Median anatomical line of propodeal spine angle value to Weber length in lateral view: 42–48°. Spine length vs. absolute cephalic size (SPST/CS): 0.227–0.281 (mean = 0.258). Minimum spine distance vs. absolute cephalic size (SPBA/CS): 0.257–0.291 (mean = 0.273). Maximum spine distance vs. absolute cephalic size (SPWI/CS): 0.280–0.331 (mean = 0.306). Apical spine distance vs. absolute cephalic size (SPTI/CS): 0.266–0.312 (mean = 0.288). Maximum mesosoma width vs. absolute cephalic size (MW/CS): 0.599–0.640 (mean = 0.615). Metanotal depression count: present. Metanotal depression shape: deep. Dorsal region of mesosoma sculpture: rugulose with areolate ground sculpture. Lateral region of pronotum sculpture: areolate ground sculpture, main sculpture forked costate. Mesopleuron sculpture: areolate ground sculpture superimposed by dispersed rugulae. Metapleuron sculpture: areolate ground sculpture superimposed by dispersed rugulae. Frontal profile of petiolar node contour line in lateral view shape: concave. Dorsal profile of petiolar node contour line angle value to frontal profile of petiole contour line in lateral view: 110–120°. Anterodorsal rim of petiole count: absent medially. Dorsal profile of petiolar node contour line in lateral view shape: slightly convex. Dorsal region of petiole sculpture: ground sculpture areolate, main sculpture dispersed rugose; ground sculpture areolate, main sculpture absent. Dorso-caudal petiolar profile contour line in lateral view shape: straight; concave. Dorsal region of postpetiole sculpture: ground sculpture areolate, main sculpture dispersed rugose; ground sculpture areolate, main sculpture absent.

**Fig 33:**
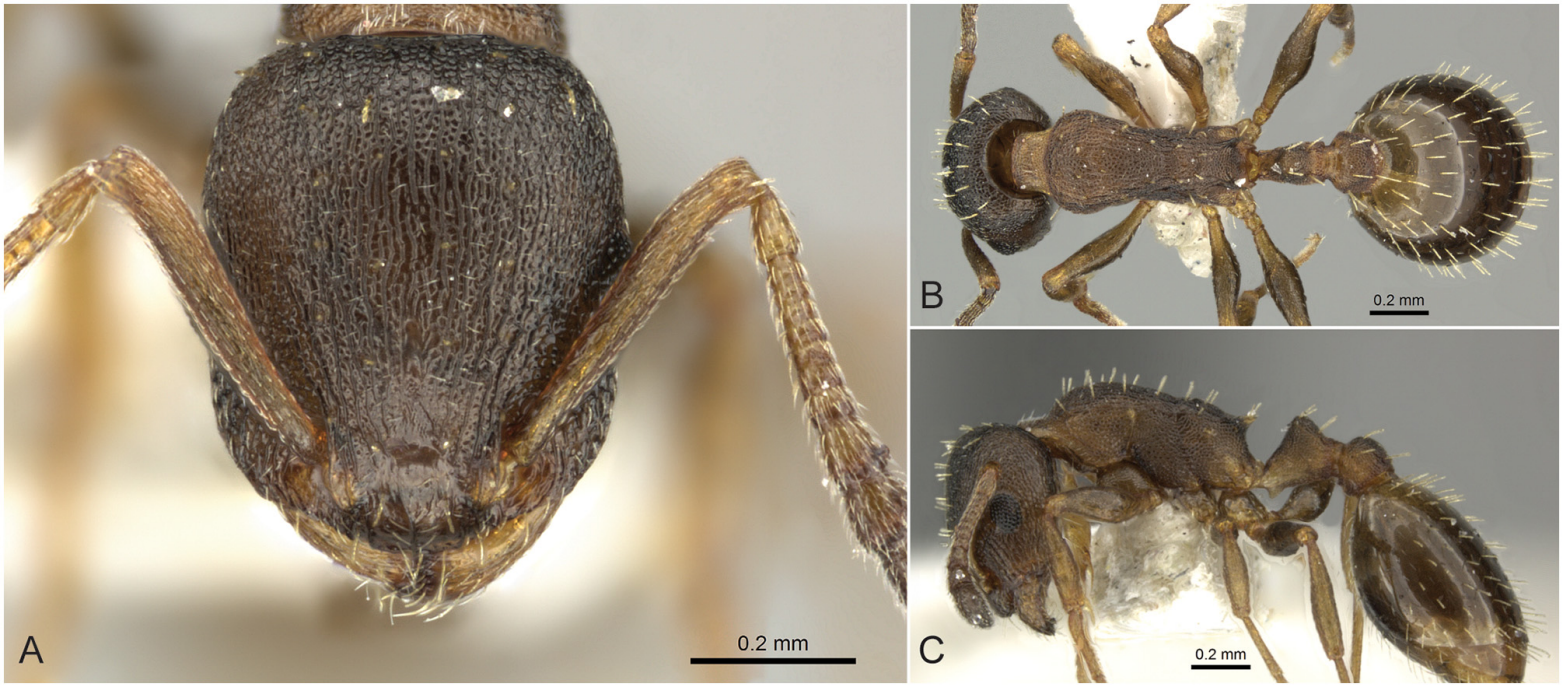
*Temnothorax sordidulus* non-type worker. Head of the holotype worker (CASENT0906712) in full face view (A), dorsal view of the body (B), lateral view of the body (C).

#### Differential diagnosis

Due to its dark brown to black color this species can only be confused with its European sibling, *T*. *tergestinus*. The other black species belonging to this complex, *T*. *artvinensis* is endemic to Turkey. Though *T*. *sordidulus* seems endemic to the Dinaran Alps, its distribution slightly overlaps with that of *T*. *tergestinus* in Slovenia and Austria. The darker, often black color of *T*. *sordidulus* in contrast to the brown color of Central European populations of *T*. *tergestinus*, its shorter spines (SPST/CS), shorter scapes (SL/CS), longer petioles (PL/CS) and petiolar nodes (NOL/CS) may help to separate *T*. *sordidulus* from Central European populations of *T*. *tergestinus*. If these ratios overlap and do not clearly separate between the two taxa, or if *T*. *sordidulus* shall be separated from Bulgarian or Greek populations of *T*. *tergestinus*, a discriminant function (D7 = +0.0323*SPTI -0.0594*NOL +0.0859*SL -0.0490*ML +0.0867*CWb -0.1167*EL -0.0414*CL -1.2164) yields 99% classification success rate in single individuals and 100% in nest sample means.

D7 scores for single individuals:

*T*. *sordidulus* (n 47) = -1.872 [-5.013, -0.191], [5–95% percentiles: -4.301, -0.500]

*T*. *tergestinus* (n 139) = +1.872 [-0.481, +4.314], [5–95% percentiles: +0.254, +3.275]

D7 scores for nest sample means:

*T*. *sordidulus* (n 16) = -1.885 [-4.154, -0.508]

*T*. *tergestinus* (n 49) = +1.868 [+0.443, +3.443]

In its restricted distributional area *T*. *sordidulus* cannot be confused with any other species treated in this revision.

#### Geographic distribution

This species has a relatively restricted distributional area and is occurs in the Dinaran Alps of Austria, Croatia and Slovenia (Fig 30).

### *Temnothorax tergestinus* (Finzi, 1928) stat. n.

*Leptothorax sordidulus* var. *tergestina* Finzi, 1928: 129 (w) ITALY.

Combination in *Temnothorax*: Bolton, 2003: 271.

Senior synonym of *Temnothorax saxonicus* (Seifert, 1995): [synonymy proposed hereby]

#### Type material investigated

**Syntype workers of *Leptothorax sordidulus* var. *tergestina* Finzi, 1928:** „S.Croze” Ven. Giulia [Trieste] B. Finzi „6.27”, FinziColl. purch 1950, M. C. Z. „co”type „28840”, „Syntypus Leptothorax sordidulus var tergestinus Finzi”, [on the reverse side: „SP Cover 98”], MCZ Museum of Comparative Zoology (4## MCZ), **[ITA:Ven-Giulia-tergestinus-TYPE]**;

**Nest sample of the holotype of *Leptothorax sordidulus saxonixus* Seifert, 1995:** GER: 51.0895 N, 14.6927 E Löbauer Berg 415m Basaltklippen mit *Quercus*, B. Seifert 1983.07.28–473 aus Holotypus-Nest (3## HNHM), **[GER:Lobauer-Berg-saxonicus-TYPE];**

The list of 132 non-type individuals belonging to 46 nest samples of other material investigated is given in S1 Table.

#### Worker (Fig 34A–34C, S1 Table, S4 Table)

Body color: brown. Body color pattern: head, mesosoma, waist and anterior region of 1st gastral tergite lighter antenna and legs except femora lighter than femora and posterior region of gaster. Antenna color pattern: clava concolorous funicle. Absolute cephalic size: 523–665 μm (mean = 592, n = 49). Cephalic length vs. Maximum width of head capsule (CL/CWb): 1.169–1.253 (mean = 1.206). Postocular distance vs. cephalic length (PoOc/CL): 0.373–0.400 (mean = 0.384). Postocular sides of cranium contour frontal view orientation: parallel; converging posteriorly. Postocular sides of cranium contour frontal view shape: broadly convex. Vertex contour line in frontal view shape: straight. Vertex sculpture: main sculpture homogenously forked costate, ground sculpture areolate. Genae contour from anterior view orientation: converging. Gena contour line in frontal view shape: feebly convex. Gena sculpture: rugoso-reticulate with areolate ground sculpture. Median region of antennal rim vs. frontal carina in frontal view structure: not fully overlapped by frontal carina. Concentric carinae laterally surrounding antennal foramen count: present. Eye length vs. absolute cephalic size (EL/CS): 0.237–0.280 (mean = 0.258). Frontal carina distance vs. absolute cephalic size (FRS/CS): 0.347–0.380 (mean = 0.361). Longitudinal carinae on median region of frons count: present. Longitudinal carinae on medial region of frons shape: forked. Smooth median region on frons count: absent. Antennomere count: 12. Scape length vs. absolute cephalic size (SL/CS): 0.781–0.824 (mean = 0.799). Facial area of the scape absolute setal angle: 0–15°. External area of the scape absolute setal angle: 30°; 35–45°. Ground sculpture of submedian area of clypeus: smooth. Median carina of clypeus count: present. Lateral carinae of clypeus count: present. Median anatomical line of propodeal spine angle value to Weber length in lateral view: 45–50°. Spine length vs. absolute cephalic size (SPST/CS): 0.220–0.335 (mean = 0.276). Minimum spine distance vs. absolute cephalic size (SPBA/CS): 0.248–0.295 (mean = 0.273). Maximum spine distance vs. absolute cephalic size (SPWI/CS): 0.283–0.372 (mean = 0.333). Apical spine distance vs. absolute cephalic size (SPTI/CS): 0.270–0.346 (mean = 0.312). Maximum mesosoma width vs. absolute cephalic size (MW/CS): 0.588–0.652 (mean = 0.620). Metanotal depression count: present. Metanotal depression shape: shallow. Dorsal region of mesosoma sculpture: areolate ground sculpture, superimposed by dispersed rugae. Lateral region of pronotum sculpture: areolate ground sculpture, main sculpture dispersed costate. Mesopleuron sculpture: areolate ground sculpture superimposed by dispersed rugulae. Metapleuron sculpture: areolate ground sculpture superimposed by dispersed rugulae. Frontal profile of petiolar node contour line in lateral view shape: concave. Dorsal profile of petiolar node contour line angle value to frontal profile of petiole contour line in lateral view: 110–120°. Anterodorsal rim of petiole count: absent medially. Dorsal profile of petiolar node contour line in lateral view shape: slightly convex. Dorsal region of petiole sculpture: ground sculpture areolate, main sculpture dispersed rugose; ground sculpture areolate, main sculpture absent. Dorso-caudal petiolar profile contour line in lateral view shape: straight; concave. Dorsal region of postpetiole sculpture: ground sculpture areolate, main sculpture dispersed rugose; ground sculpture areolate, main sculpture absent.

**Fig 34:**
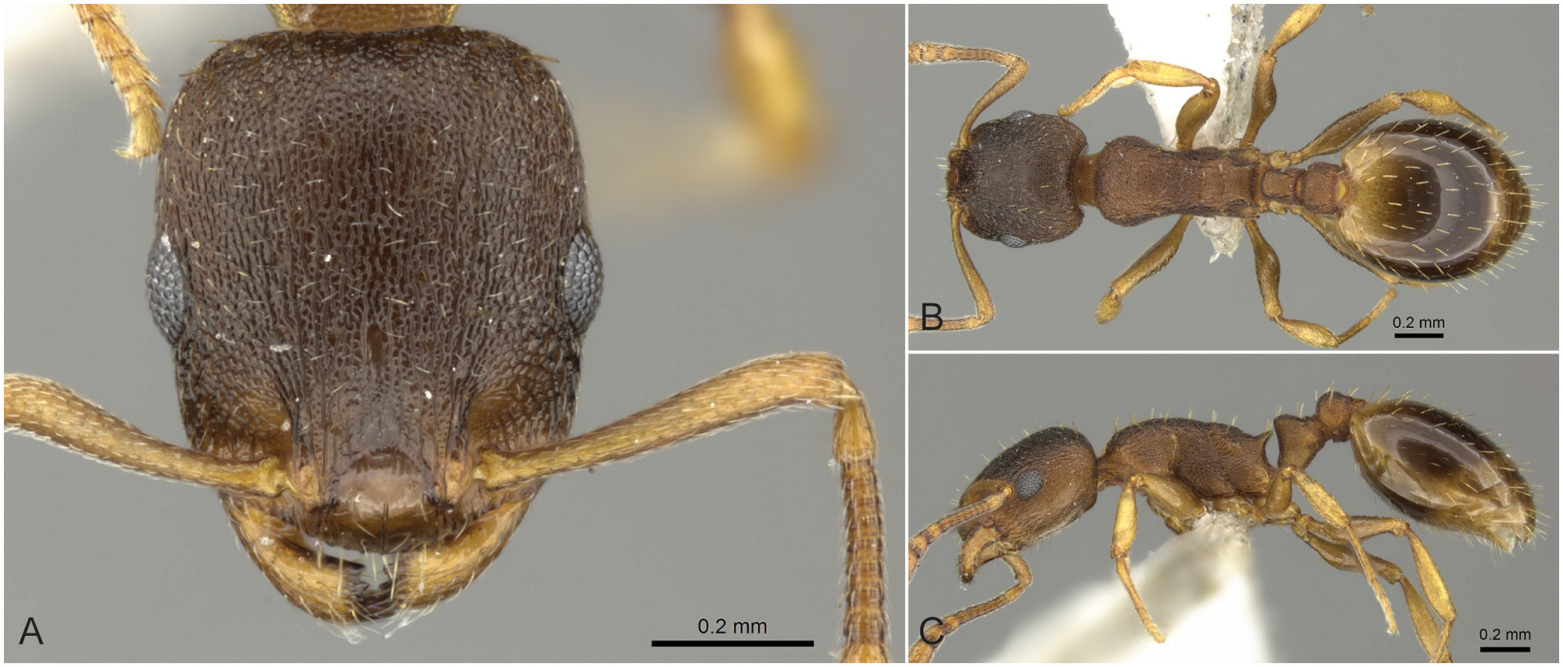
*Temnothorax tergestinus* non-type worker. Head of the holotype worker (CASENT0906708) in full face view (A), dorsal view of the body (B), lateral view of the body (C).

#### Differential diagnosis

This species can be separated from members of other species complexes by the ruguloreticulate main sculpture on head dorsum that turns irregular on the vertex and the sides of the head. In some Western and Central European populations, the surface sculpturing might be less conspicuous, which may lead to possible confusion with *T*. *parvulus* and *T*. *nylanderi*, particularly if the body surface is covered by diffuse dust.

Weakly sculptured, lightly colored specimens of *T*. *tergestinus* can be safely separated from *T*. *nylanderi* using PoOC/CL and non-overlapping SPWI/CS ratios (S4 Table). Slightly overlapping NOH/CS, SPTI/CS and SPWI/CS ratios (S4 Table) help to distinguish *T*. *tergestinus* and *T*. *parvulus* samples. This species is not supposed to be confused with other members of other species complexes by combination of various traits.

*Temnothorax tergestinus* shares most of its characters with *T*. *sordidulus* and *T artvinensis*. The latter is separated from *T*. *tergestinus* both by the broad gap in their distribution range (Fig 30) and by discriminant (D4) function (for details see differential diagnosis of *T artvinensis*). A discriminant function (D7) that helps separating *T*. *tergestinus* from *T*. *sordidulus* is given in differential diagnosis under the latter.

#### Nomenclatural issues

A multivariate analyses of 22 continuous characters in 186 individuals of 64 nest samples (Fig 2, S5 Table, Figs 28 and 29) confirmed the earlier hypothesis [31] that two separate species exist: *T*. *sordidulus* (Müller, 1923) and *T*. *saxonicus* (Seifert, 1995). Our results, however, differ from the earlier [31] view in two major points: a) the Bulgarian populations do not belong to *T*. *sordidulus*, but grouped in a cluster that was formerly referred to as *T*. *saxonicus* (sensu Seifert [31]); b) in this novel classification, the type series of *T*. *tergestinus* (Finzi, 1928) is nested in the *T*. *saxonicus* (sensu Seifert [31]) cluster with a posterior probability of p = 0.98. The type series of *T*. *saxonicus* (Seifert, 1995) fell in the same cluster with a posterior probability of p = 0.99. Therefore, we propose a new junior synonymy for *Temnothorax saxonicus* (Seifert, 1995) with *Temnothorax tergestinus* (Finzi, 1928) stat. n.

The newly outlined species boundaries allow a considerably easier separation of *T*. *tergestinus* and *T*. *sordidulus* (Figs 28 and 29), which underpins the increased robustness of the new classification. A combination of 7 traits yields a perfect discrimination (see also differential diagnosis in *T*. *sordidulus*), in contrast to the 18 characters that was used to require earlier [31].

#### Geographic distribution

According to our newly recognized pattern *T*. *tergestinus* spreads from France to Bulgaria and Greece (Fig 30).

## Supporting Information

S1 Table List of samples of ants *Temnothorax nylanderi* species-complex morphometrically investigated. All samples can be uniquely distinguished by sample-specific abbreviations (e.g., **GRE:Levidi-10S-20000427-123**; for details see main text).
(XLSX)

S2 Table GenBank accession numbers. GenBank accession number of CO I sequences of ants of *Temnothorax nylanderi* species-complex.
(FAS)

S3 Table Morphometric dataset. Morphometric characters of worker individuals of *Temnothorax nylanderi* species-complex. Data are given in μm.
(CSV)

S4 Table Basic statistics of morphometric data. Nest sample means of continuous morphometric data are calculated for the eighteen species of *Temnothorax nylanderi* species-complex treated in this revision. Mean of indices, ±SD are provided in the upper row, minimum and maximum values are given in parentheses in the lower row.
(XLSX)

S5 Table Classification matrix. Identification matrix calculated by Linear Discriminant Analysis and Leave One Out Cross Validation for 1693 workers of *Temnothorax nylanderi* species-complex investigated in this revisionary work is shown.
(XLSX)

S6 Table Semantic statements of natural language phenotypes. The list of natural language phenotypes used in the species descriptions of *Temnothorax nylanderi* species-group is provided with their semantic representations in Manchester Syntax format.
(XLSX)
